# Non-FDG PET/CT in Diagnostic Oncology: a pictorial review

**DOI:** 10.1186/s41824-019-0066-2

**Published:** 2019-11-29

**Authors:** Francesco Giammarile, Paolo Castellucci, Rudi Dierckx, Enrique Estrada Lobato, Mohsen Farsad, Roland Hustinx, Amirreza Jalilian, Olivier Pellet, Susana Rossi, Diana Paez

**Affiliations:** 1 Department of Nuclear Sciences and Applications, International Atomic Energy Agency, Vienna, Austria; 2 Department of Nuclear Medicine, Sant’Orsola-Malpighi Hospital, 40138 Bologna, Italy; 3 Medical Imaging Center, Department of Nuclear Medicine and Molecular Imaging, University Medical Center Groningen, Groningen, The Netherlands; 4 Department of Nuclear Medicine, Bolzano Hospital, Bolzano, Italy; 5 Department of Nuclear Medicine, CHU Liège, University of Liège, Liège, Belgium; 6 Centro Uruguayo de Imagenología Molecular (CUDIM), Montevideo, Uruguay

**Keywords:** PET/CT, Non-FDG, Pictorial

## Abstract

Positron emission tomography/computed tomography (PET/CT) is currently one of the main imaging modalities for cancer patients worldwide. Fluorodeoxyglucose (FDG) PET/CT has earned its global recognition in the modern management of cancer patients and is rapidly becoming an important imaging modality for patients with cardiac, neurological, and infectious/inflammatory conditions.

Despite its proven benefits, FDG has limitations in the assessment of several relevant tumours such as prostate cancer. Therefore, there has been a pressing need for the development and clinical application of different PET radiopharmaceuticals that could image these tumours more precisely. Accordingly, several non-FDG PET radiopharmaceuticals have been introduced into the clinical arena for management of cancer. This trend will undoubtedly continue to spread internationally. The use of PET/CT with different PET radiopharmaceuticals specific to tumour type and biological process being assessed is part of the personalised precision medicine approach.

The objective of this publication is to provide a case-based method of understanding normal biodistribution, variants, and pitfalls, including several examples of different imaging appearances for the main oncological indications for each of the new non-FDG PET radiopharmaceuticals. This should facilitate the interpretation and recognition of common variants and pitfalls to ensure that, in clinical practice, the official report is accurate and helpful.

Some of these radiopharmaceuticals are already commercially available in many countries (e.g. ^68^Ga-DOTATATE and DOTATOC), others are in the process of becoming available (e.g. ^68^Ga-PSMA), and some are still being researched. However, this list is subject to change as some radiopharmaceuticals are increasingly utilised, while others gradually decrease in use.

## Radioisotopes

*Carbon-11* is a PET radioisotope with a T1/2 of 20.4 min. Due to the abundance of carbon in the chemistry of biomolecules, all C-11 radiopharmaceuticals demonstrate identical behaviour to natural compounds, allowing real tracing of the biological processes.

*Fluorine-18* is a PET radioisotope with a T1/2 of 109.7 min. Due to high chemical stability of the C-F bond in organic compounds, and the high water solubility of F-compounds, F-18 tracers usually exhibit suitable stability and biodistribution in humans. The vast clinical application of F-compounds has led to the development of efficient automated production methods of F-18 tracers for clinical use.

*Gallium-68* has a T1/2 of 67.7 min, and is usually obtained from a germanium-68 generator. Due to the T1/2 of 271 days of the parent isotope, 68Ge, the generator can be used for in-hospital production of Ga-68.

## Radiopharmaceuticals

### Acetate

Names: CH_3_[^11^C]O_2_, ^11^C-acetate

**Fig. 1:**
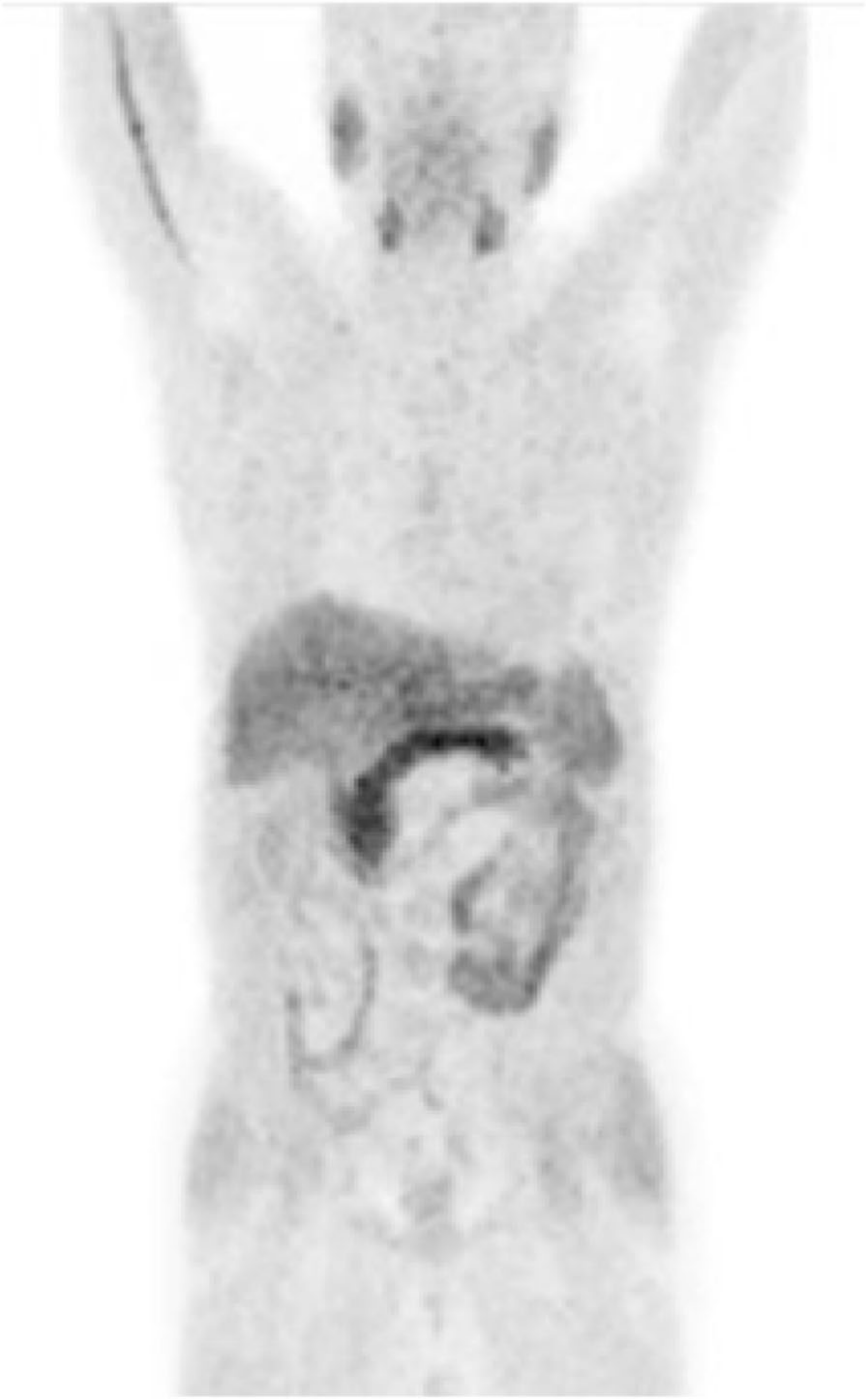
Physiological bio-distribution of ^11^C-acetate

Biodistribution and metabolism (Fig. 1)

After injection ^11^C-acetate is dispersed in many human tissues including the pancreas, bowels, liver, kidneys, and spleen. The tracer is not excreted in urine under normal circumstances. ^11^C-acetate is typically incorporated into the cellular membrane in proportion to the cellular proliferation rate or alternatively oxidised to carbon dioxide and water. ^11^C-acetate may also be converted into amino acids (Seltzer et al. 2004; Karanikas and Beheshti 2014).

Scan acquisition

• Fast of 4 h is suggested

• 4 or 5 MBq\Kg of ^11^C-acetate iv

• Uptake time 10–20 min

• Acquisition starts from the pelvis

**Fig. 2:**
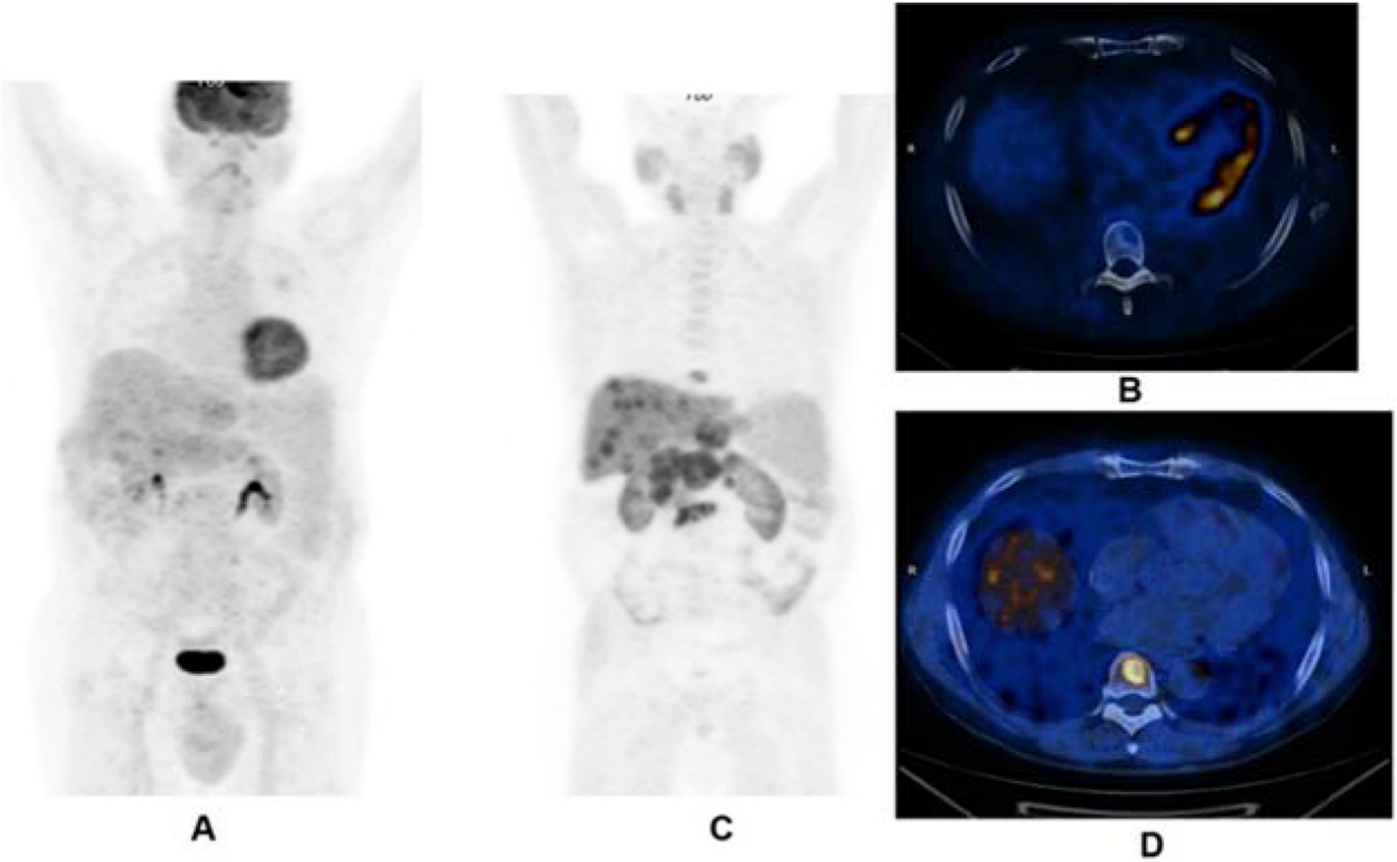
^11^C-acetate, staging hepatocellular carcinoma (HCC), comparison with 18F-FDG. *Clinical history*: 78 y.o. man with metastatic HCC after liver transplantation, patient underwent acetate and FDG study in a single day examination. *PET/CT findings*: acetate: multiple areas of increased uptake in the liver, lymph nodes, and bones consistent with lytic lesions at CT images. No FDG uptake. **a** FDG MIP. **b** FDG fused images. **c** acetate MIP. **d** acetate fused images

**Fig. 3:**
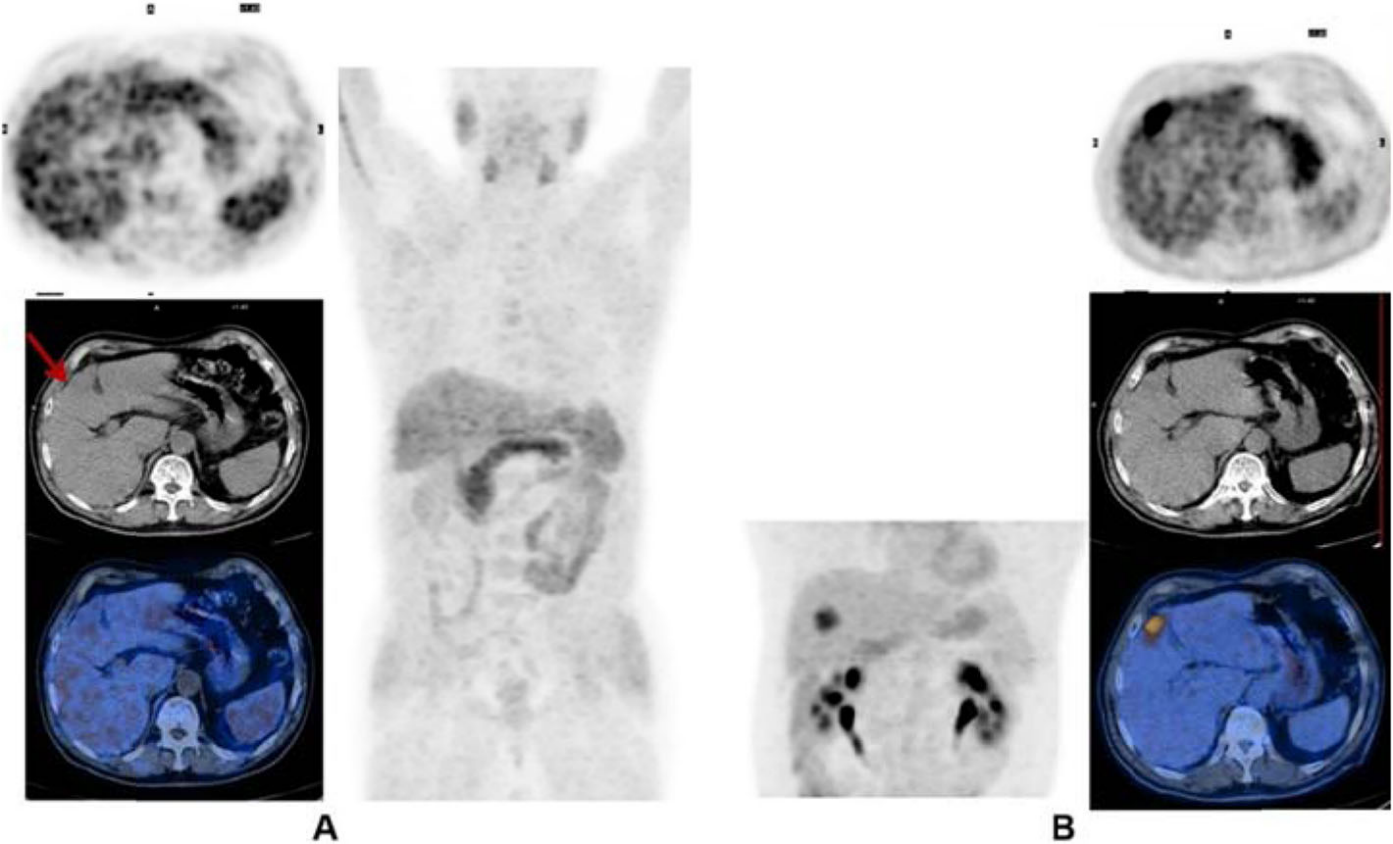
^11^C-acetate, staging hepatocellular carcinoma (HCC), comparison with ^18^F-FDG. *Clinical history*: 69 y.o. patients with HCC at presentation. Staging of a poorly differentiated HCC, patient underwent acetate and FDG study in a single day examination. *PET/CT findings*: acetate (**a** MIP and fused images): no areas of significant tracer uptake in the liver. The hypodense lesion in the IV segment (CT images red arrow) is consistent with the primary HCC. FDG (**b** MIP and fused images): increased tracer uptake in the IV segment (CT images red arrow) is consistent with the primary HCC

Clinical indications in oncology (Figs. 2 and 3)

The main clinical application of ^11^C-acetate is the detection of non ^18^F-FDG-avid neoplasm, such as differentiated hepatocellular carcinoma and renal cell carcinomas (Hain and Maisey 2003; Ho et al. 2003; Park et al. 2008). Some other applications of ^11^C-acetate PET are brain tumours (Liu et al. 2006) and lung carcinomas, while in the past the tracer has been used in prostate cancer (Sandblom et al. 2006).

### FES

Names: 16α-[^18^F] Fluoro-17β-estradiol; 16-Fluoroestradiol, ^18^F-fluoroestradiol

**Fig. 4:**
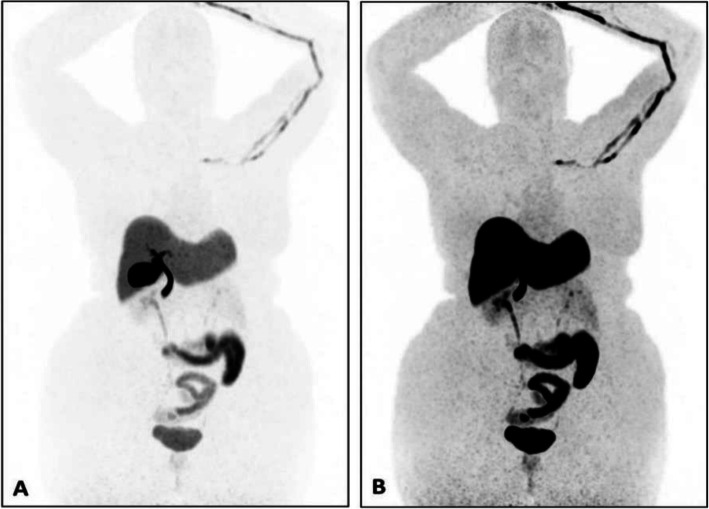
Physiological bio-distribution of ^18^F-fluoroestradiol. Low (**a**) and high (**b**) intensity MIP images

Biodistribution and metabolism (Fig. 4)

After injection, the tracer is cleared from the blood and metabolised in 20 min. ^18^F-fluoroestradiol binds to the oestrogen receptors on the tumour cell surface as well as intratumoural receptors in oestrogen receptor-positive tumours (Liao et al. 2016).

Scan acquisition

- Treatment with oestrogen receptor antagonists (e.g. tamoxifen, fulvestrant, faslodex, oestrogens) should be suspended for at least 5 weeks prior to performing the scan. Aromatase inhibitors and luteinizing hormone releasing hormone agonists may be continued
- No fasting is required
- 200 MBq of ^18^F-fluoroestradiol iv
- Level of binding of ^18^F-FES to the oestrogen receptors remains stable between 20 and 120 min postinjection. For logistical reasons, scanning procedure should start 60 min after injection

**Fig. 5:**
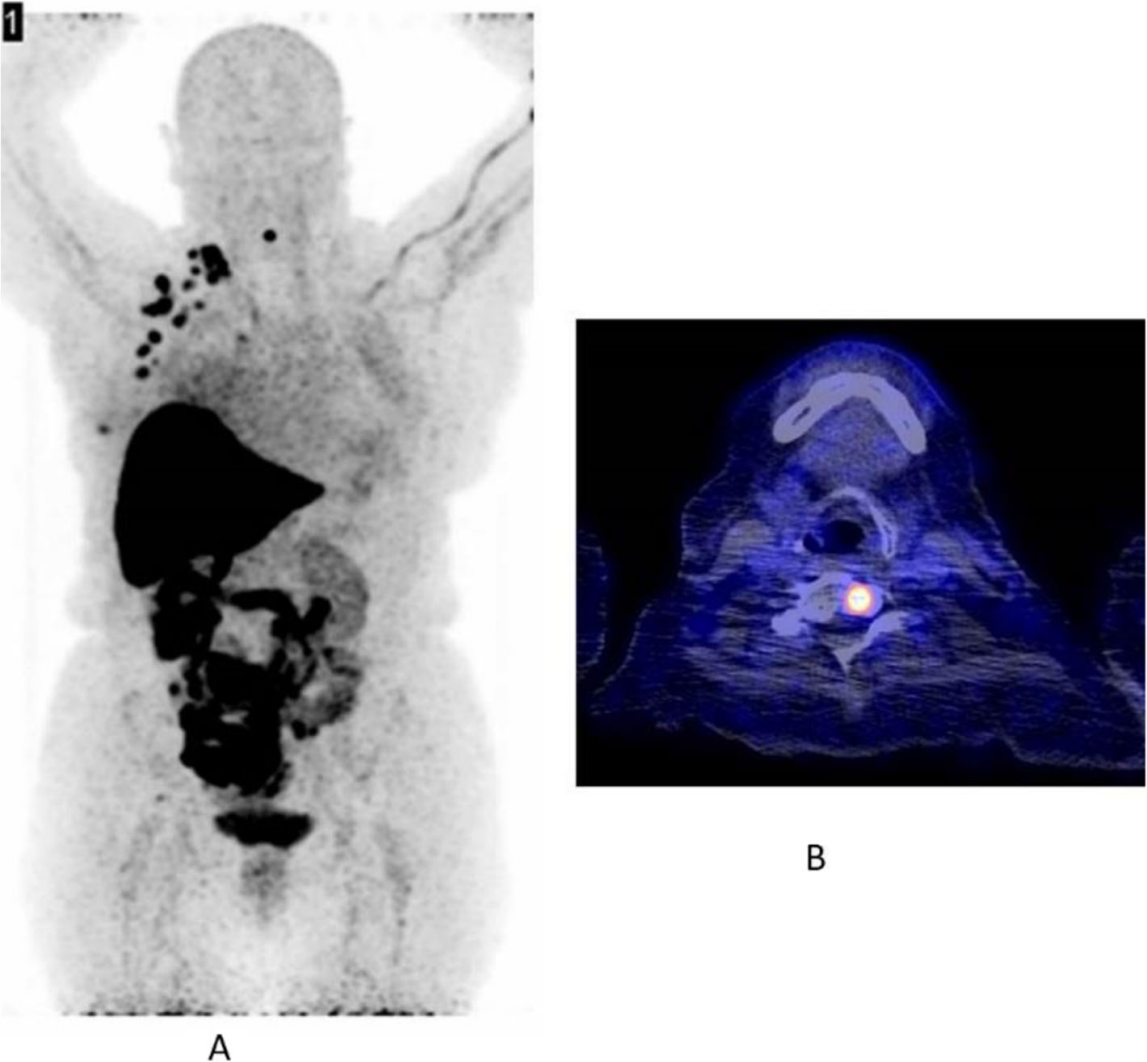
^18^F-fluoroestradiol, restaging breast cancer. *Clinical history*: 46 y.o. woman with proven breast cancer on the right side (ER+). Palpable lymph nodes in the right axilla. *PET/CT findings*: ER expression visible in the primary breast tumour and in several lymph nodes in the right axilla and in the right clavicular region in MIP (**a**); one focal lesion with increased ER expression in a cervical vertebra in fused images (**b**)

**Fig. 6:**
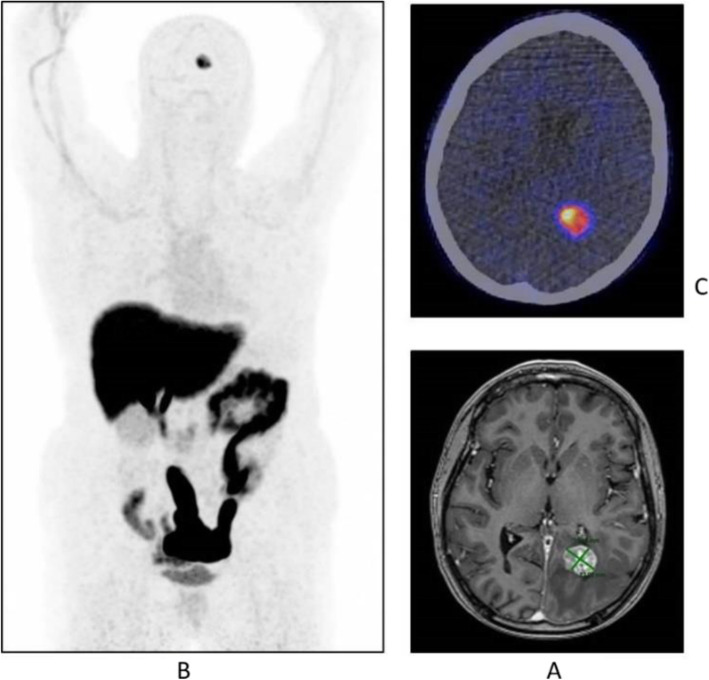
^18^F-fluoroestradiol, breast cancer, characterisation of brain metastasis. *Clinical history*: 53 y.o. woman with history of colon cancer (2004) and breast cancer (2011), ER+, presenting with an 18-mm brain lesion on MRI (**a**). Biopsy was not possible due to location. *PET/CT findings*: solitary lesion with increased ER expression in the brain in MIP (**b**), located in the left occipital lobe on fused images (**c**), suggesting brain metastasis from breast cancer

Clinical indications in oncology (Figs. 5 and 6)

^18^F-fluoroestradiol is a valuable tracer for the studies of the oestrogen receptor status of primary and metastatic breast or ovarian cancers (Venema et al. 2016; van Kruchten et al. 2013a; van Kruchten et al. 2012; van Kruchten et al. 2013b; Peterson et al. 2011; Linden et al. 2011).

### FET

Names: O-(2-[^18^F] Fluoroethyl)-L-tyrosine; ^18^F-fluoroethyltyrosine

**Fig. 7:**
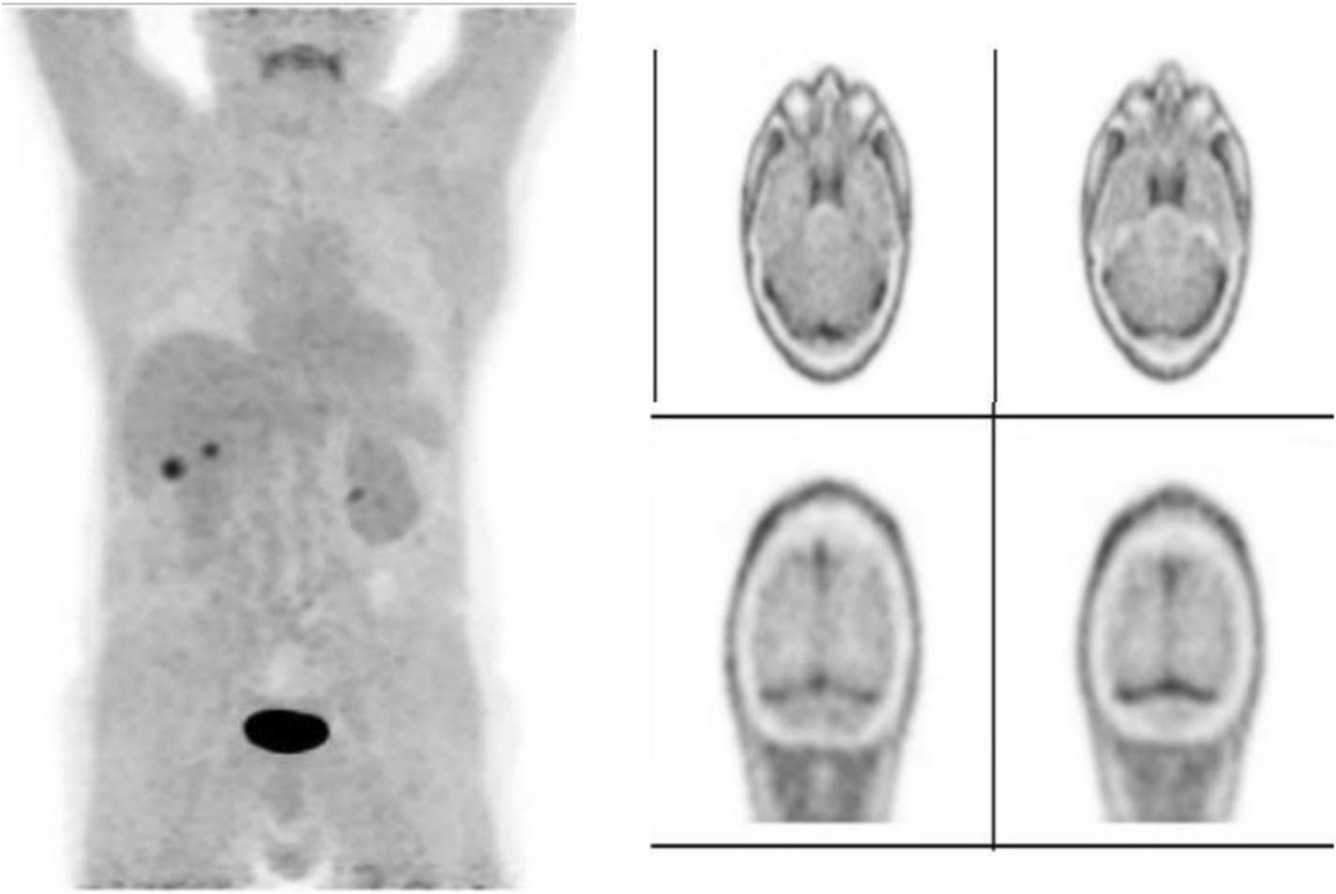
Physiological biodistribution of ^18^F-FET

Biodistribution and metabolism (Fig. 7)

^18^F-FET is an amino-acid PET tracer. After injection, the tracer is trapped into cancerous cells, though it is not incorporated into proteins (Abe et al. 2006).

Scan acquisition

• Fasting for at least 4 h is required

• 4–5 MBq\Kg of ^18^F-FET iv

• Dynamic one bed brain acquisition for 40 min or static one bed brain acquisitions at 10 and 40–50 min. after injection, for 10 min.

**Fig. 8:**
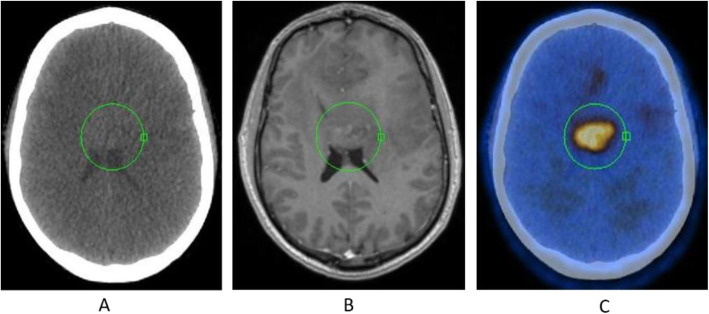
^18^F-FET, evaluation of a brain lesion. *Clinical history*: 46 y.o. man with nausea and headache. CT was non-informative (**a**). MRI shows an infiltrating lesion with low Gd enhancement (**b**). *PET/CT findings*: high FET uptake (**c**). Surgery confirmed a high-grade glioma

**Fig. 9:**
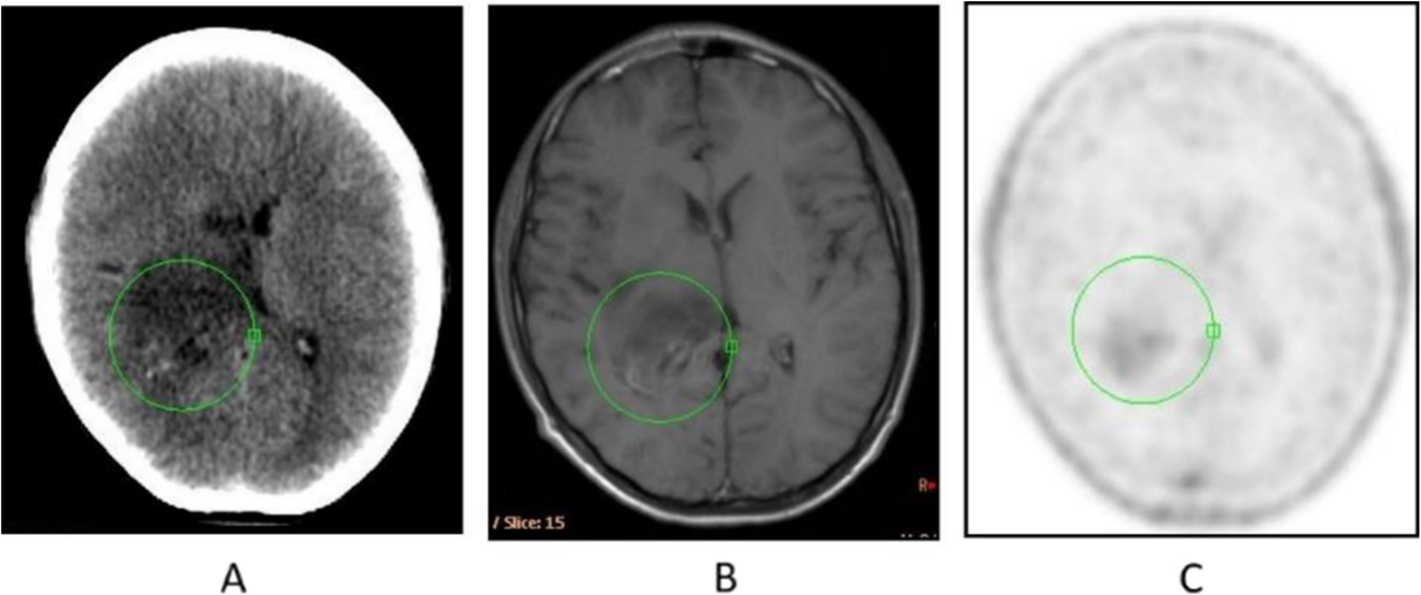
^18^F-FET, evaluation of a brain lesion. *Clinical history*: 46 y.o. man with seizure. CT (**a**) and MRI (**b**) shows an infiltrating lesion in the right parietal region (**a**). *PET/CT findings*: faint FET uptake in PET (**c**). Surgery confirmed a low-grade glioma

**Fig. 10:**
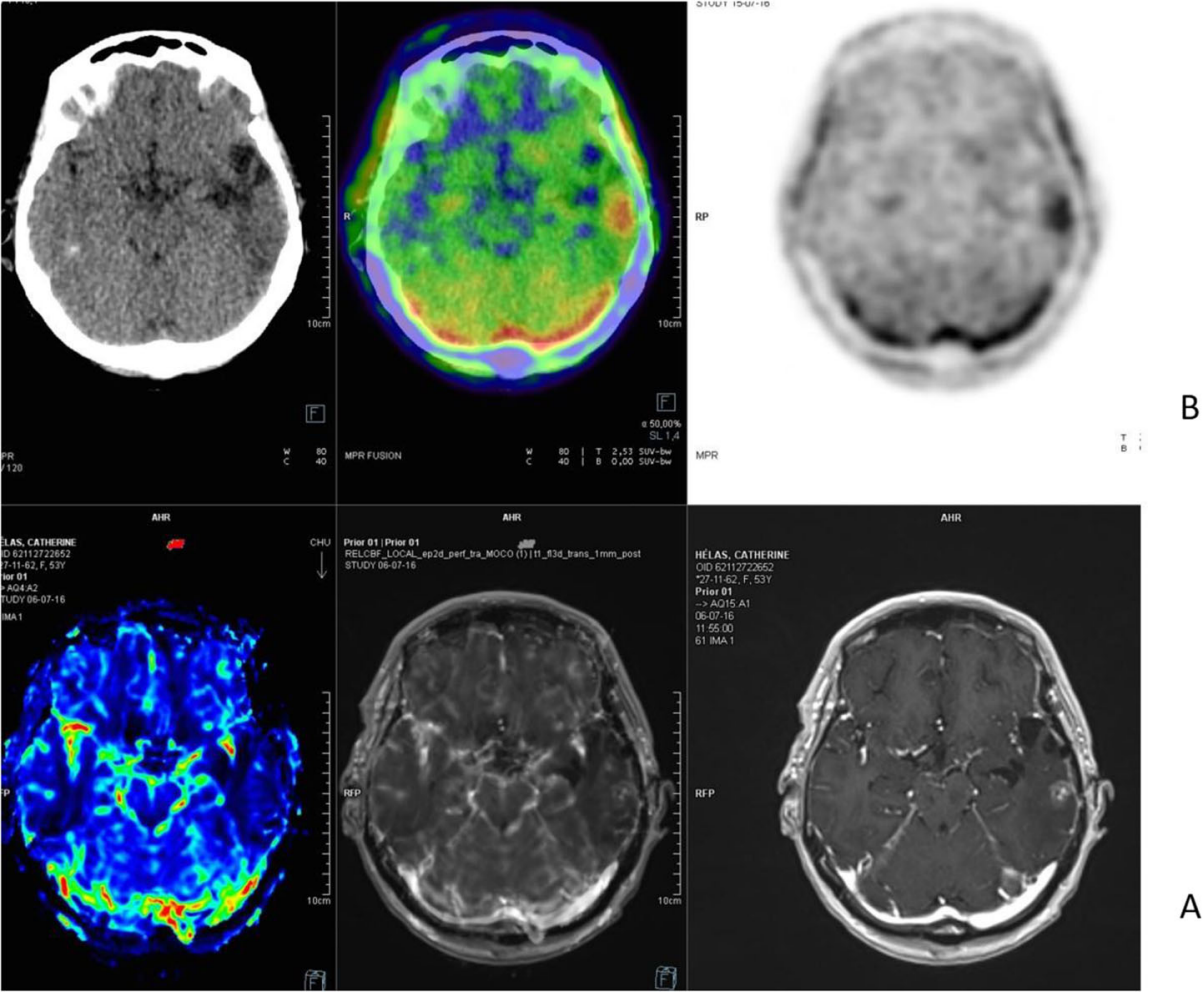
^18^F-FET, suspicion of recurrence. *Clinical history*: 55 y.o. woman, with grade II–III oligo-astrocytoma treated by surgery and adjuvant radiation therapy 13 months prior. MRI shows a focal area of Gd enhancement but no corresponding perfusion anomaly, consistent with radiation necrosis (**a**). *PET/CT findings*: highly increased FET uptake in the lesion, consistent with persistent/recurrent tumour on PET and fused images (**b**). Surgery revealed grade II oligo-astrocytoma

Clinical indications in oncology (Figs. 8, 9, and 10)

Diagnosis of central nervous system tumours (very low background in healthy brain) (Galldiks et al. 2015; Albert et al. 2016; Unterrainer et al. 2016; Kunz et al. 2011; Poulsen et al. 2017).

### FLT

Names: 3′-deoxy-3′-[^18^F]-fluorothymidine; ^18^F-fluorothymidine

**Fig. 11:**
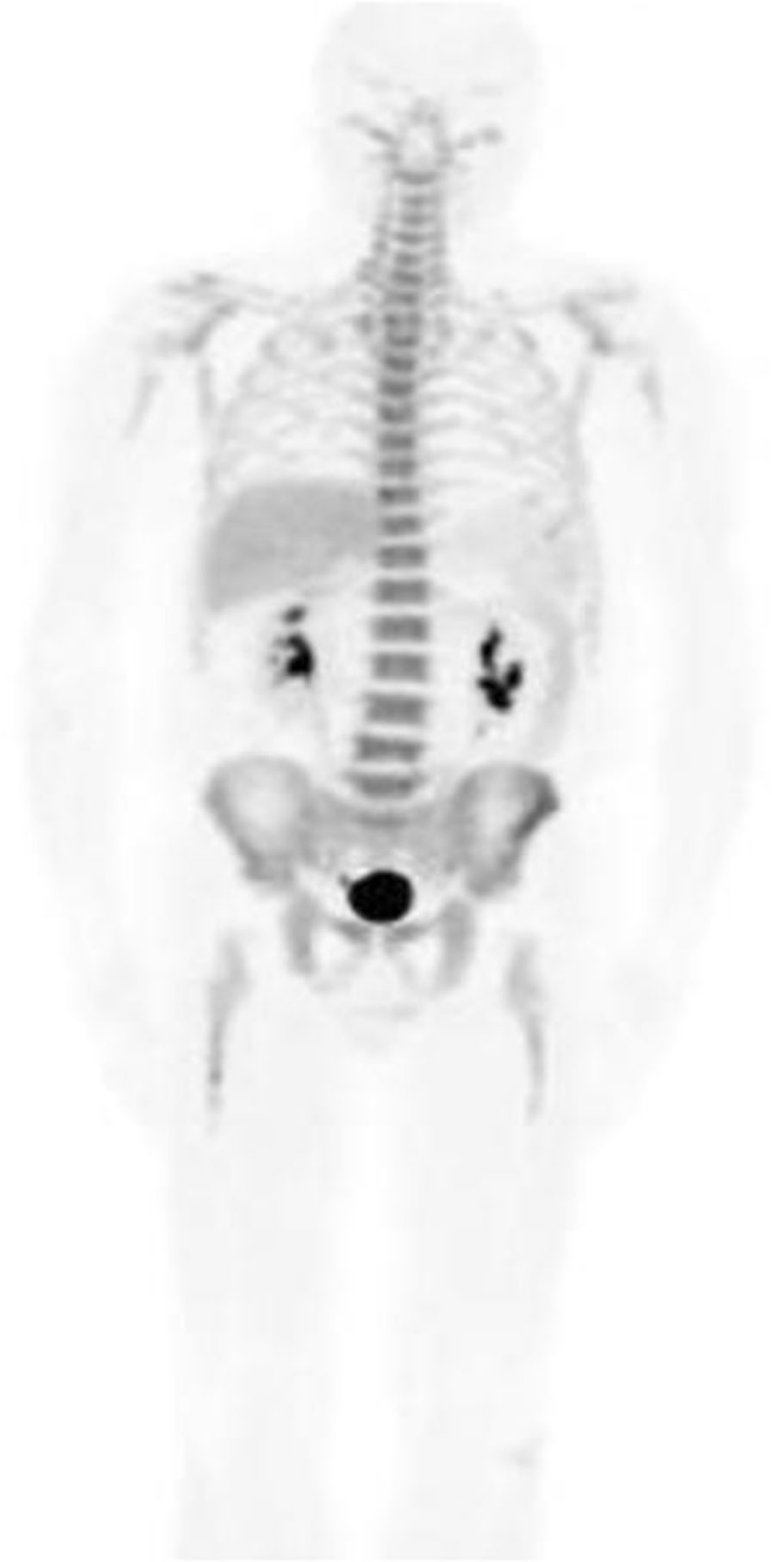
Physiological biodistribution of ^18^F-FLT

Biodistribution and metabolism (Fig. 11)

^18^F-FLT is an analogue of the nucleoside thymidine; however, substitution of the 3′-F atom prevents from further entering the regular biochemical pathway. FLT is transported from the blood into cells by active transport and phosphorylated by thymidine kinase I without incorporation into the DNA. The conjugated FLT is cleared via the kidneys and excreted in the urine. The accumulated activity in the cells is proportional to thymidine kinase 1 activity as well as cellular proliferation (Grierson and Shields 2000; Oh et al. 2004; Shankar 2012; Turcotte et al. 2007; Vesselle et al. 2003).

Scan acquisition

• No fasting is required

• 2–3 MBq\Kg of ^18^F-FLT iv

• Uptake time 50–60 min

**Fig. 12:**
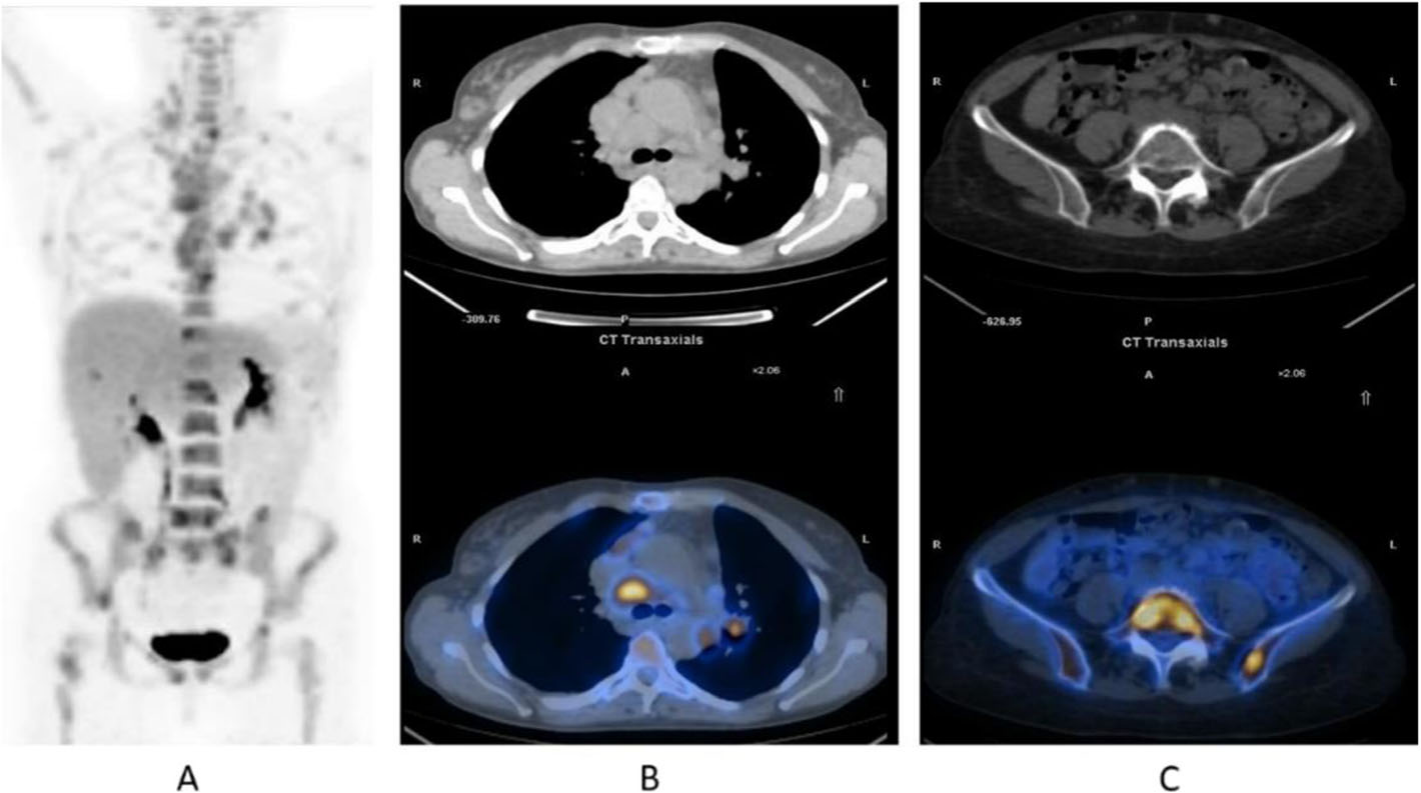
^18^F-FLT, NSCLC, before second line therapy. *Clinical history*: 57 y.o. patient with NSCLC who progressed after first line chemotherapy. *PET/CT findings*: intense tracer uptake in the left lung and in multiple large mediastinal lymph nodes and small bone lesions. **a** MIP. **b** CT and fused images of the thorax. **c** CT and fused images of the pelvis

**Fig. 13:**
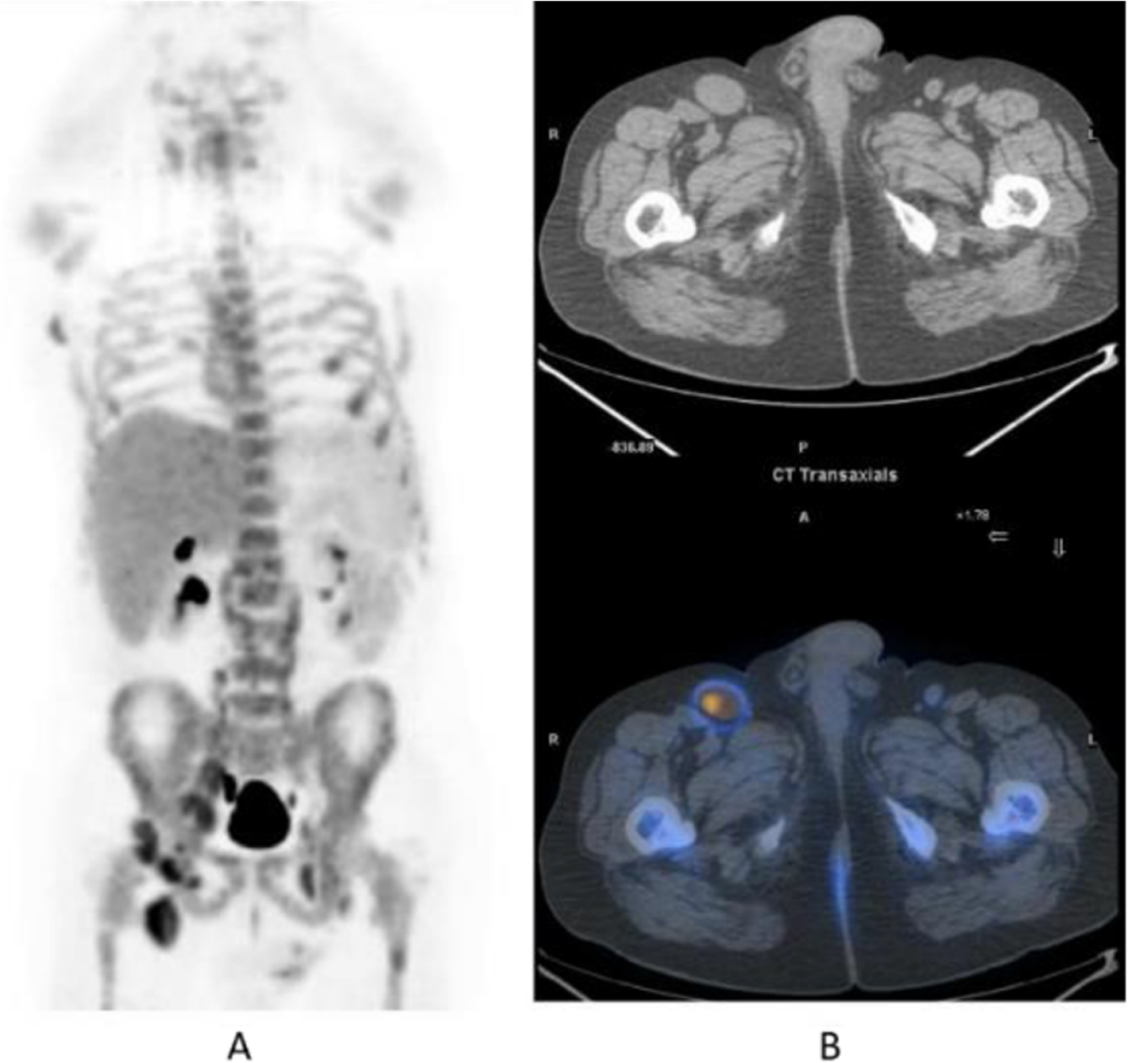
^18^F-FLT, non-Hodgkin lymphoma, staging. *Clinical history*: 52 y.o. patient with non-Hodgkin lymphoma at presentation. *PET/CT findings*: intense tracer uptake in enlarged inguinal and right iliac chain nodes. **a** MIP. **b**CT and fused images

**Fig. 14:**
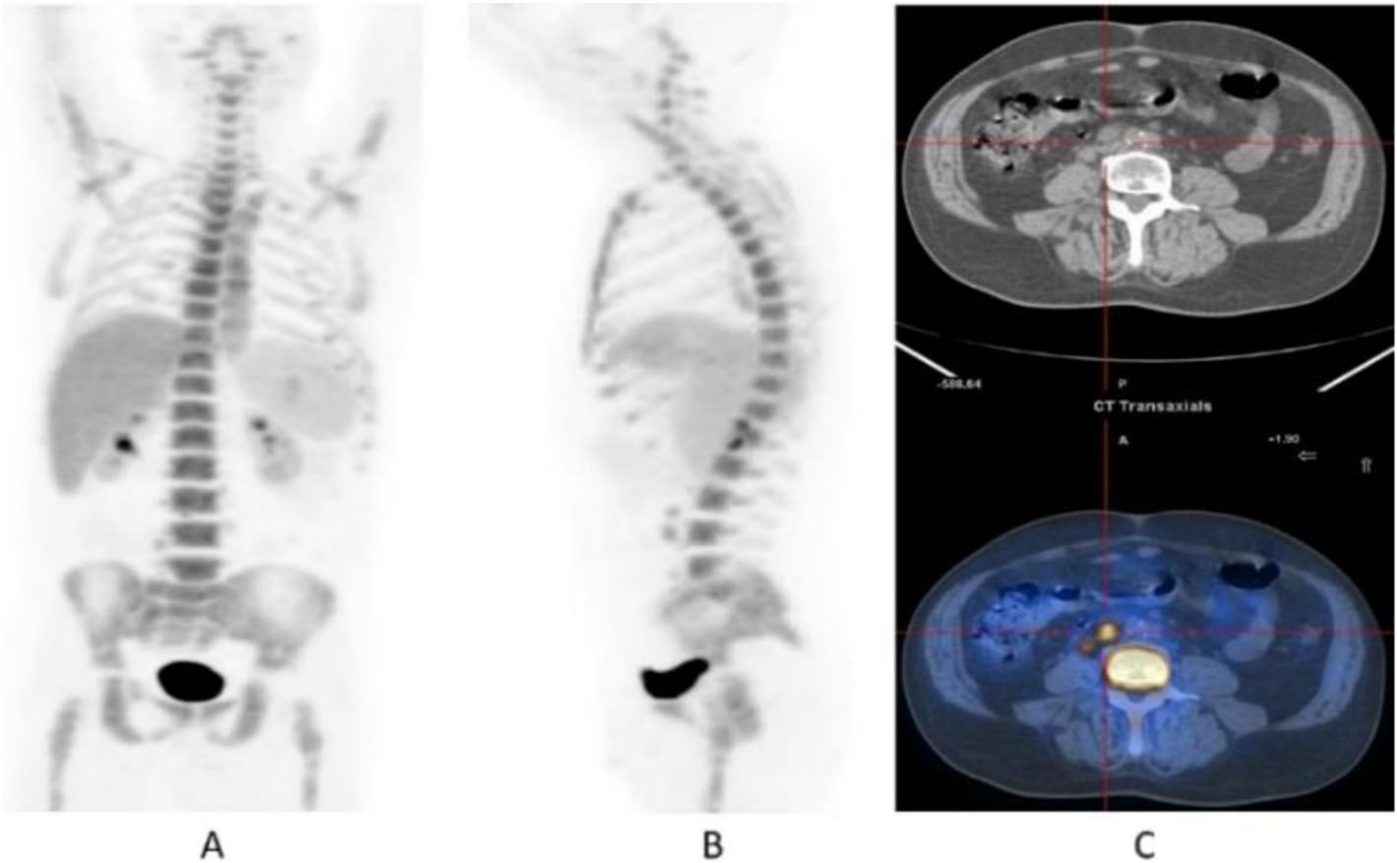
^18^F-FLT, Hodgkin lymphoma, response evaluation. *Clinical history*: 32 y.o. patient with Hodgkin lymphoma after second-line therapy. *PET/CT findings*: moderate uptake of the tracer in two retroperitoneal lymph nodes. **a** MIP. **b** MIP in a sagittal view. **c** CT and fused images

Clinical indications in oncology (Figs. 12, 13, and 14)

^18^F-FLT is a marker for tumour cell proliferation that has been introduced to improve the accuracy of early FDG PET assessment (Kenny et al. 2007).

### Methionine

Names: L-[methyl-^11^C] Methionine; ^11^C-Methionine

**Fig. 15:**
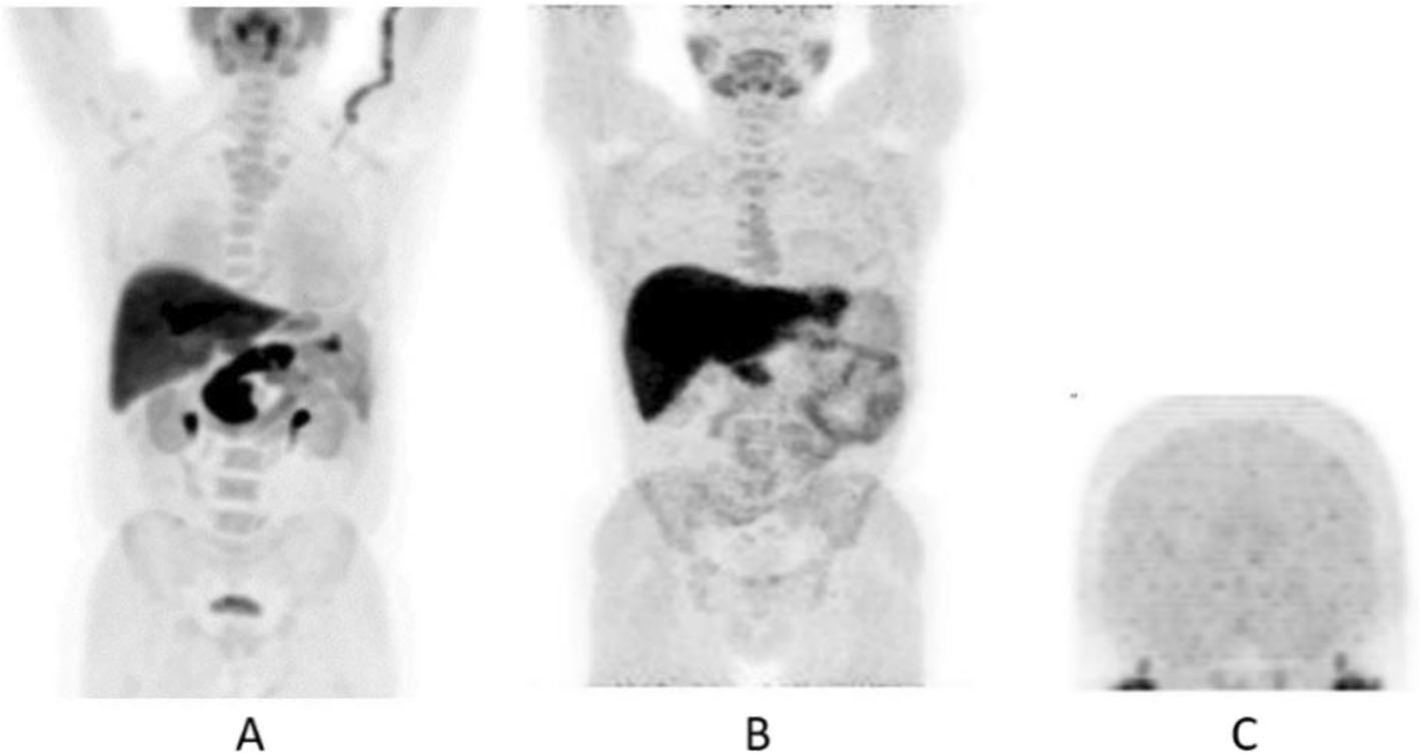
Physiological biodistribution of 11C-Methionine. MIP at 10 min (**a**) and 20 min (**b**) after administration; brain (**c**): there is only faint tracer uptake in the brain. There is low, variable uptake in the bowel. In most cases, no radioactive urine is detected in the ureters or bladder at 20 mins after injection

Biodistribution and metabolism (Fig. 15)

^11^C-Methionine, an essential amino acid, enters the cells by various aminoacid transporters and is involved in the synthesis of proteins and lipids, as well as in the regulation and synthesis of DNA and RNA (Davis et al. 1982; Deloar et al. 1998; Harris et al. 2013).

Scan acquisition

• Fasting for at least 2 h

• 3 MBq/kg of ^11^C-Methionine iv

• Injection immediately before the start of the emission

**Fig. 16:**
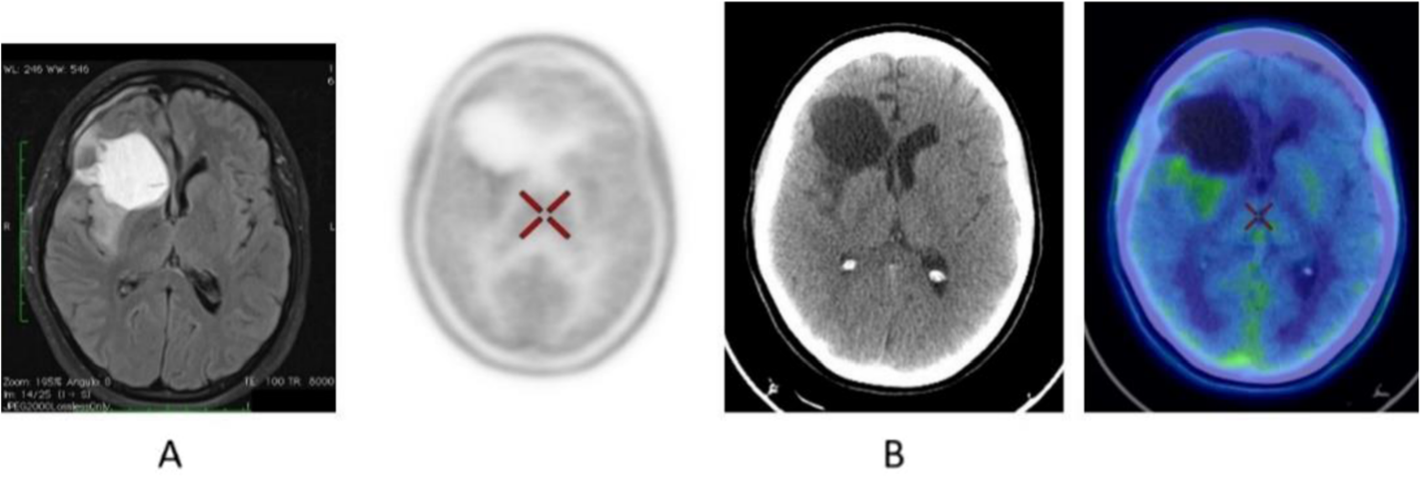
^11^C-Methionine, Glioblastoma grade 2, surgical changes. *Clinical history*: 25 y.o. female. Glioblastoma grade 2. Surgical treatment with macroscopically complete resection of right frontal glioblastoma. MRI: T2-FLAIR image shows heterogeneous hyperintensity (**a**) and T1 using Gd shows peripheral contrast enhancement. *PET/CT findings*: slight tracer uptake in margins of surgical field, compatible with inflammatory activity in PET/CT images (**b**)

**Fig. 17:**
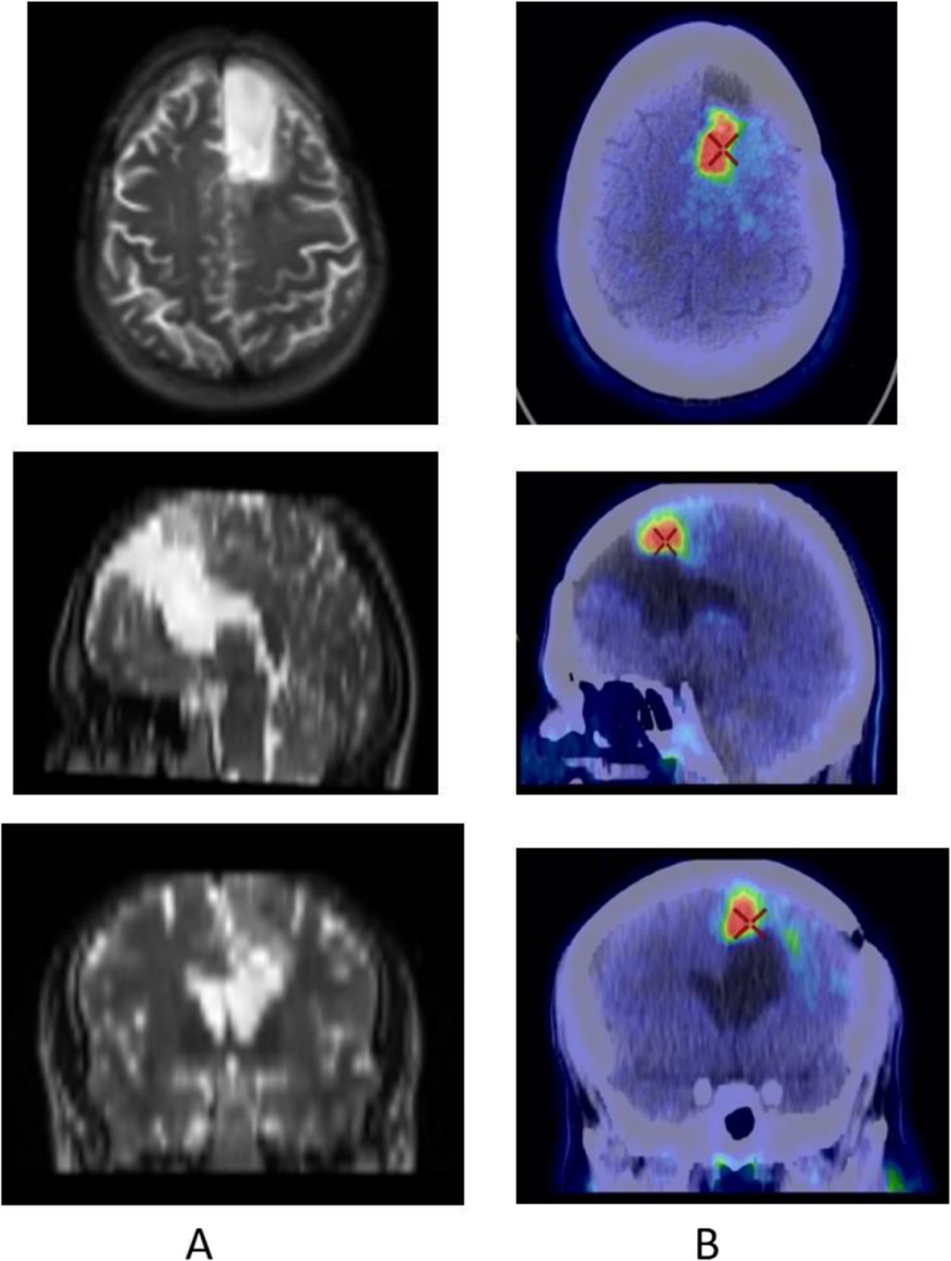
^11^C-Methionine, suspicion of recurrence. *Clinical history*: 41 y.o. male. Left frontal grade III oligoastrocytoma partially removed and treated with radiotherapy and chemotherapy. Asymptomatic for 3 years until relapse. After chemotherapy, MRI showed tumour growth, nodule in external surgical cavity wall, multiple Gd positive foci in left frontal lobe and a smaller one right anterior parasagittal. It is not possible to differentiate between gliosis or relapse (**a**). *PET/CT findings*: intense ^11^C-Methionine uptake in left frontal lobe related to tumour relapse, which extends to adjacent white matter (**b**)

**Fig. 18:**
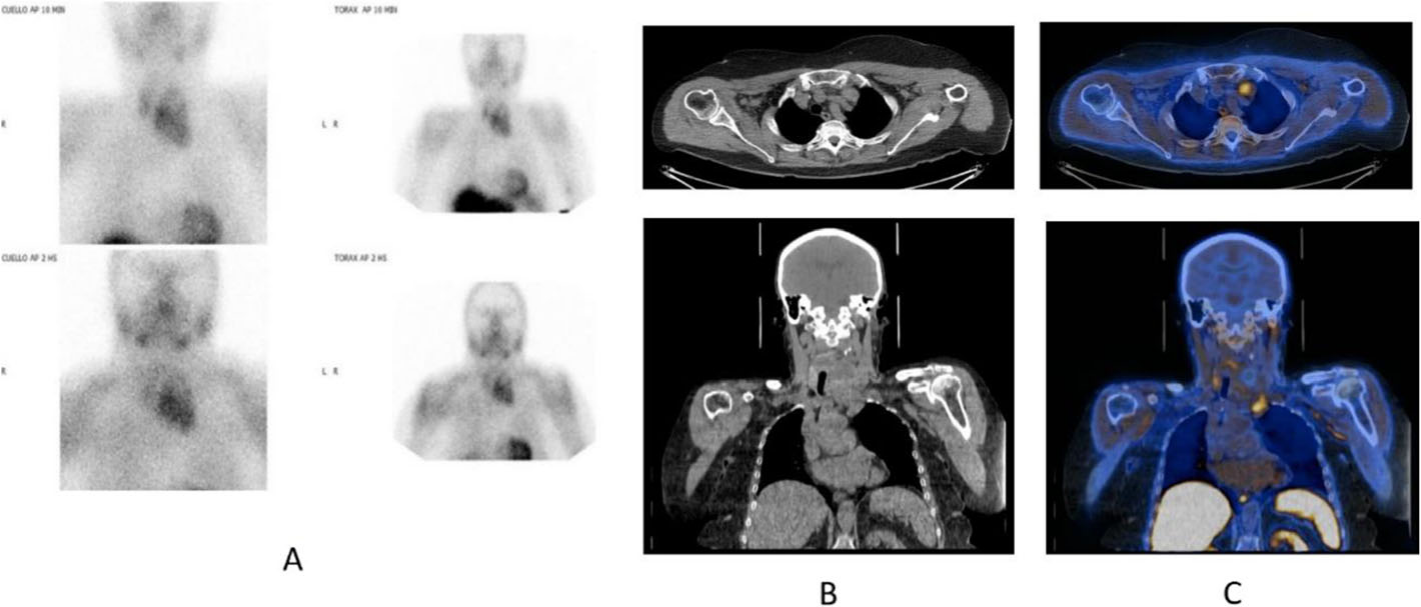
^11^C-Methionine, Primary hyperparathyroidism. *Clinical history*: 74 y.o. female patient with chronic renal failure and goitre. Parathyroid hormone 68 pg/mL. 99mTc-MIBI SPECT: multinodular goitre that extends to the thorax with a left moderate uptake nodule. There is no evidence of hyperfunctioning parathyroid tissue (**a**). *PET/CT findings*: multinodular goitre with a large mass in the left lobe that displaces the trachea to the right on CT (**b**). In the anterolateral and lower left section of this mass there is a nodule with focal tracer uptake without a clear plane of cleavage with the gland on fused images (**c**). These findings suggest a parathyroid adenoma

Clinical indications in oncology (Figs. 16, 17, and 18)

^11^C-Methionine is used in the detection of brain tumours, primarily gliomas. The gliomas present an increased protein metabolism and capture ^11^C-Methionine through specific carriers, in contrast to normal tissues that show low uptake.

### Choline

Names:

1. [11C]CH, ^11^C-choline
2. [^18^F]CH, ^18^F-fluorocholine

**Fig. 19:**
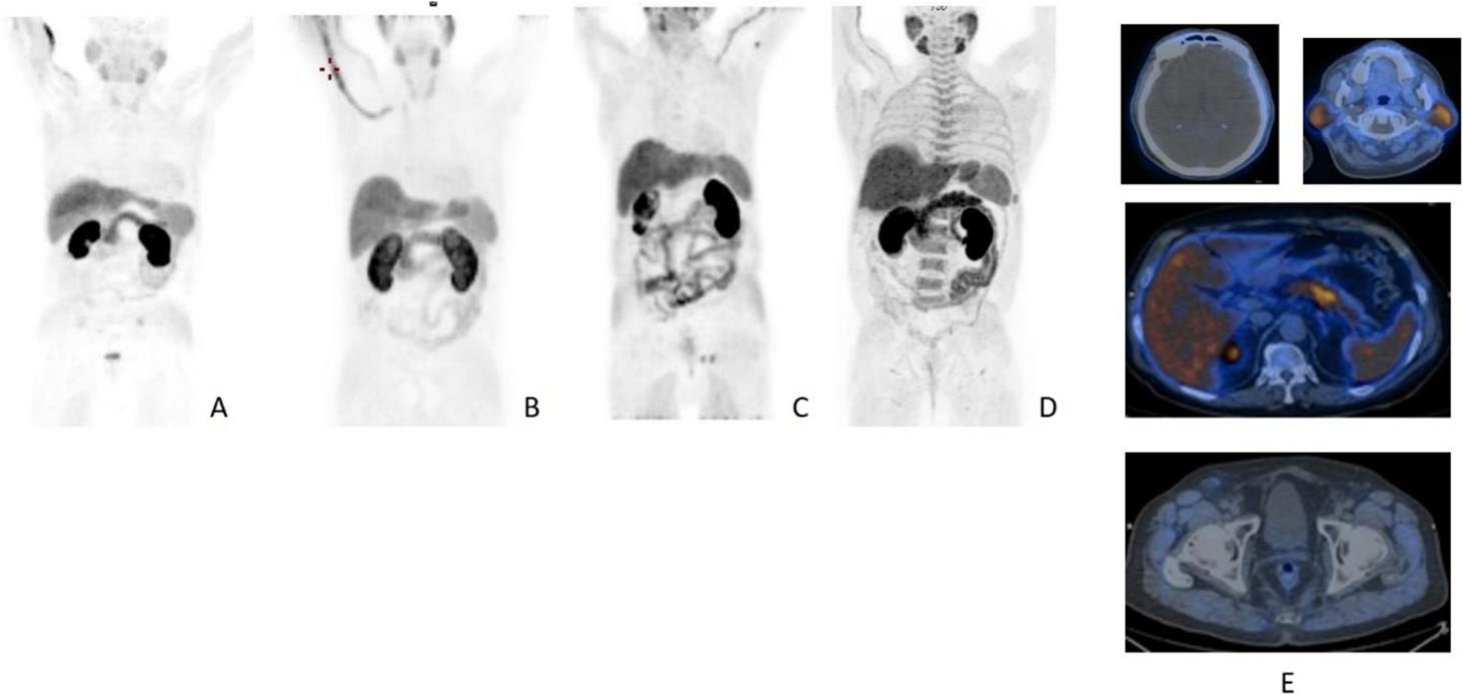
Physiological bio-distribution and normal variants of ^11^C-choline 2-5 min after administration: main findings. **a** Normal biodistribution but a small amount of radioactive urine is present in the bladder; mild uptake in the thyroid. **b** The presence of intense uptake in the vessels in which the tracers has been injected is a relatively common finding; some mild thyroid uptake is present. **c** Moderate uptake in the bowel may be present. **d** Some diffuse faint uptake in the bone marrow may be present especially after treatments as a bone marrow rebound

Biodistribution and metabolism (Fig. 19)

After injection, the tracer rapidly clears from the circulation (< 3 min), with high clearance by liver and kidneys. Increased metabolism will lead to an increased uptake of choline in the cell membranes and tissues.

^11^C-choline distributes mainly to the pancreas, kidneys, liver, spleen, and colon. Based upon the relatively low urinary excretion of radioactivity, renal distribution is predominantly to the organ itself, rather than via formation of urine.

The urinary excretion of ^18^F-fluorocholine has been reported to be about 5% of the administered activity in female patients and 2% in male patients within 60 min after injection (Mitterhauser et al. 2005; DeGrado et al. 2001; DeGrado et al. 2002).

Scan acquisition

• Fasting of 4 h is suggested

• 4 or 5 MBq\Kg of ^11^C-choline iv/300 MBq ^18^F-fluorocholine iv

• Uptake time 2–5 min for ^11^C-choline/30 min for ^18^F-fluorocholine

• Acquisition starts from the pelvis for ^11^C-choline/head-thorax for ^18^F-fluorocholine

**Fig. 20:**
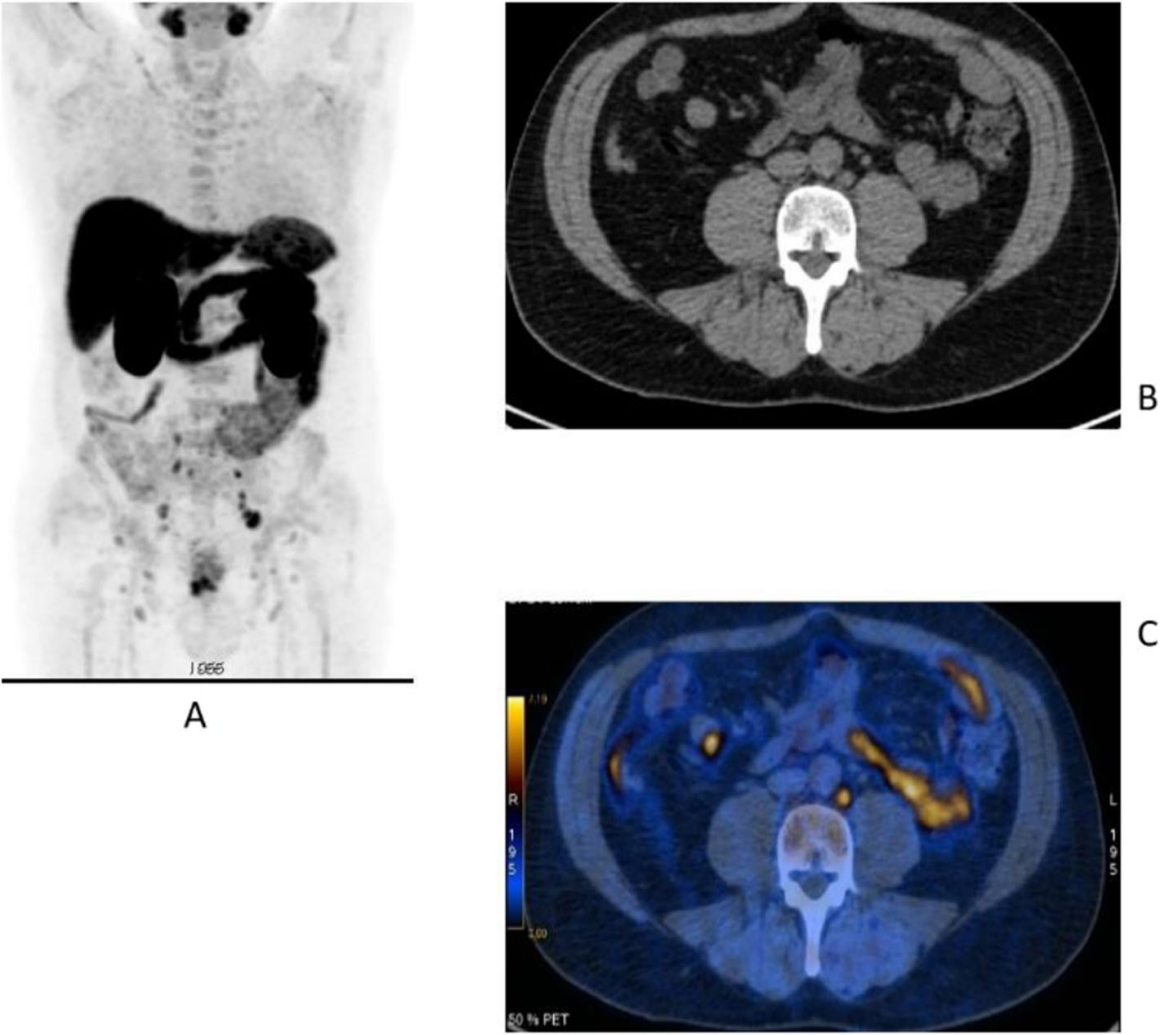
^11^C-choline, Initial staging: in very high-risk prostate cancer. *Clinical history*: 74 y.o. man with prostate cancer, Gleason score 4 + 5 according to biopsy. PSA = 126 ng/ml, T3a according to TRUS, candidate for radical prostatectomy. *PET/CT findings*: multiple foci of increased uptake seen through the pelvis and retroperitoneum, representing pathological uptake in the prostate and lymph node metastases. However, there is no evidence of osseous or visceral involvement. MIP (**a**), CT (**b**), and fused images (**c**)

**Fig. 21:**
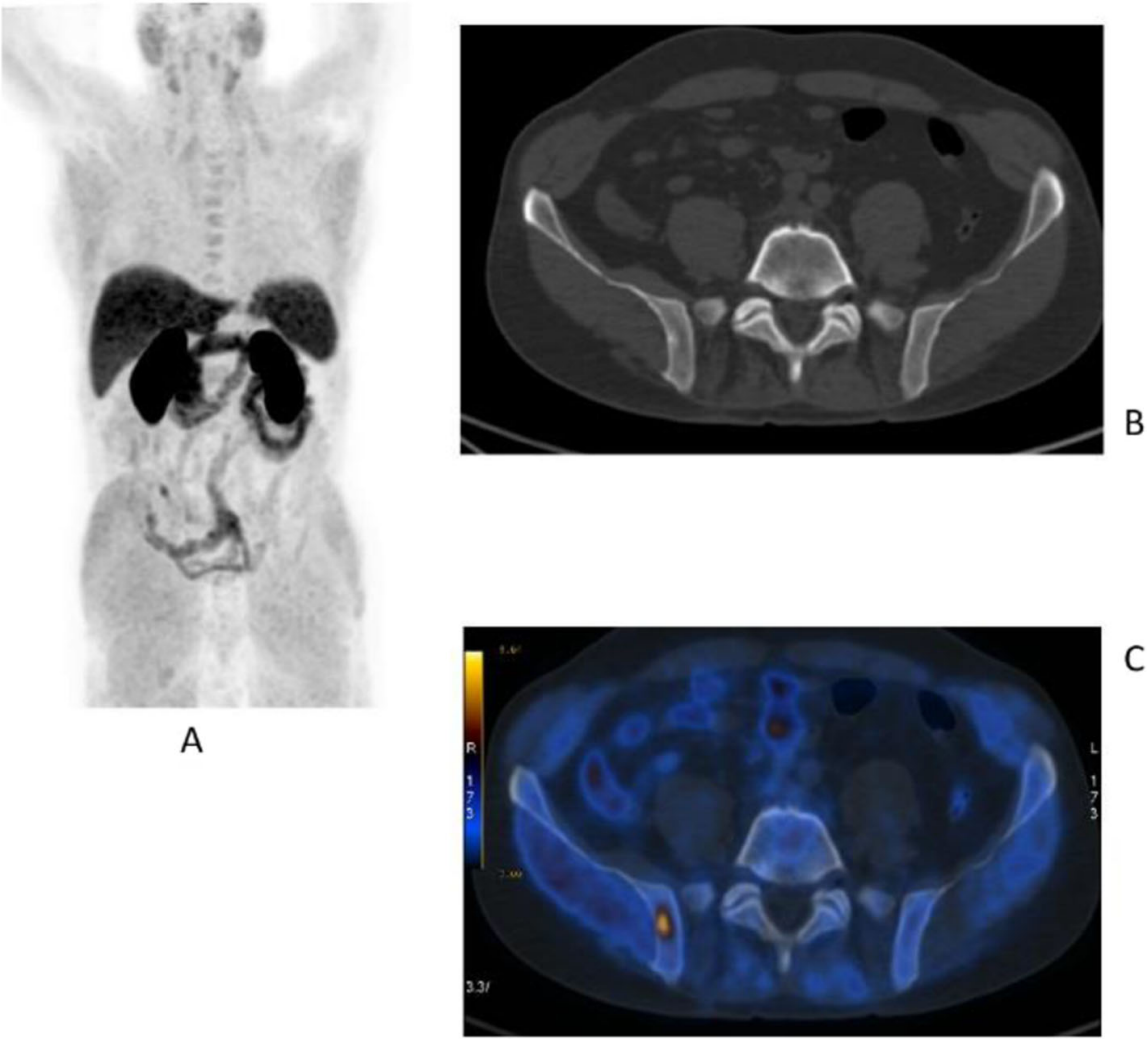
^11^C-choline, biochemical recurrence (BCR). *Clinical history*: 70 y.o. man with prostate cancer, Gleason score 4 + 3, treated with radical prostatectomy as primary treatment; BCR with a TTR 48 months and PSA 0.6 ng/ml PSAdt 5 months. *PET/CT findings*: focal increased uptake seen in the right iliac bone. MIP (**a**), CT (**b**), and fused images (**c**) showed a very small osteoblastic lesion

**Fig. 22:**
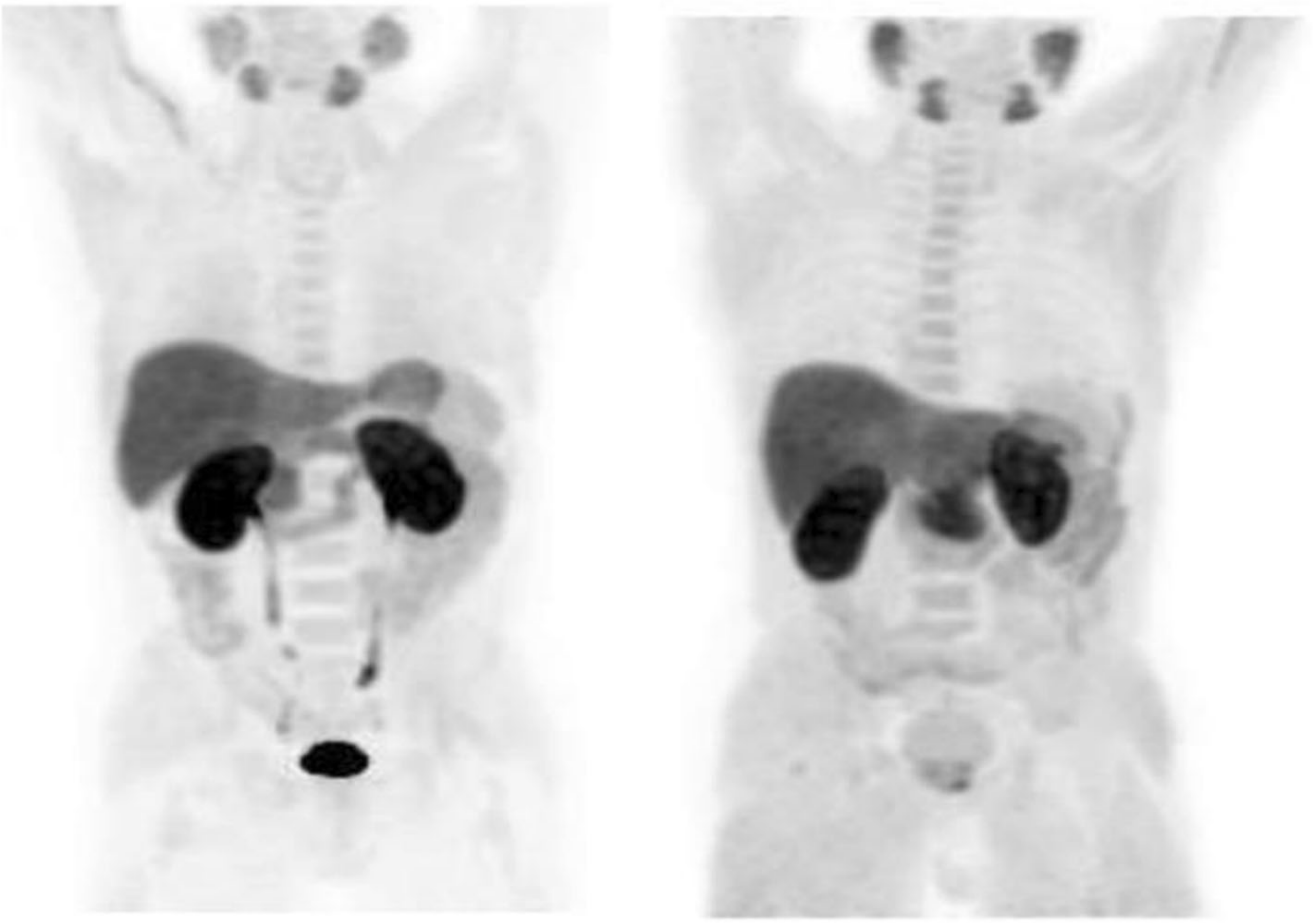
Physiological biodistribution of ^18^F-fluorocholine

**Fig. 23:**
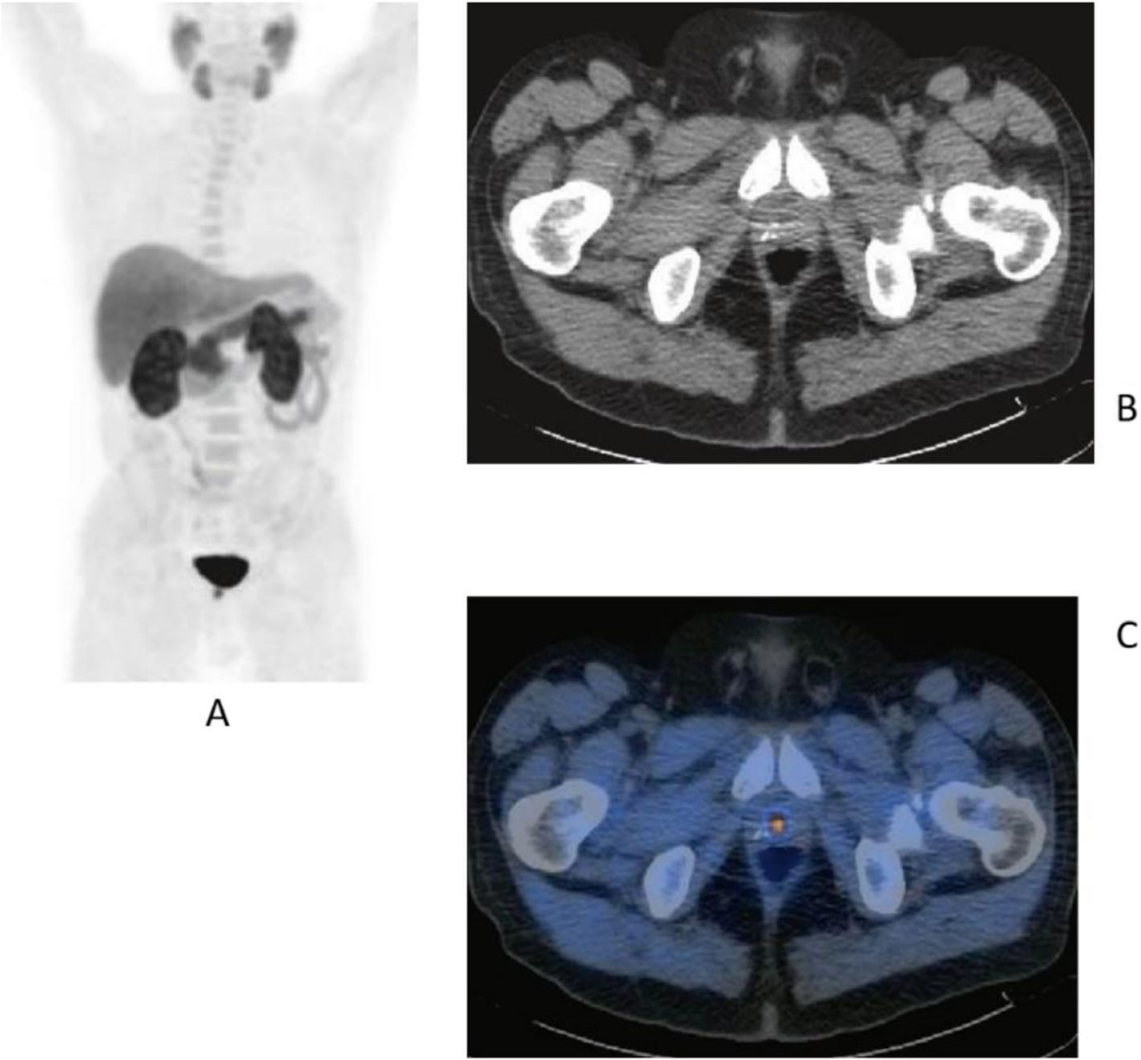
^18^F-fluorocholine, prostate cancer, biochemical recurrence (BCR). *Clinical history*: 72 y.o. man with prostate cancer, Gleason score 3 + 3, treated with radiation therapy. Patient underwent transuretral prostate resection prior to radiotherapy. BCR with a TTR 14 months and PSA 0.8 ng/ml PSAdt 13 months. *PET/CT findings*: focal increased uptake below the bladder seen on MIP (**a**), CT (**b**), and fused images (**c**)

**Fig. 24:**
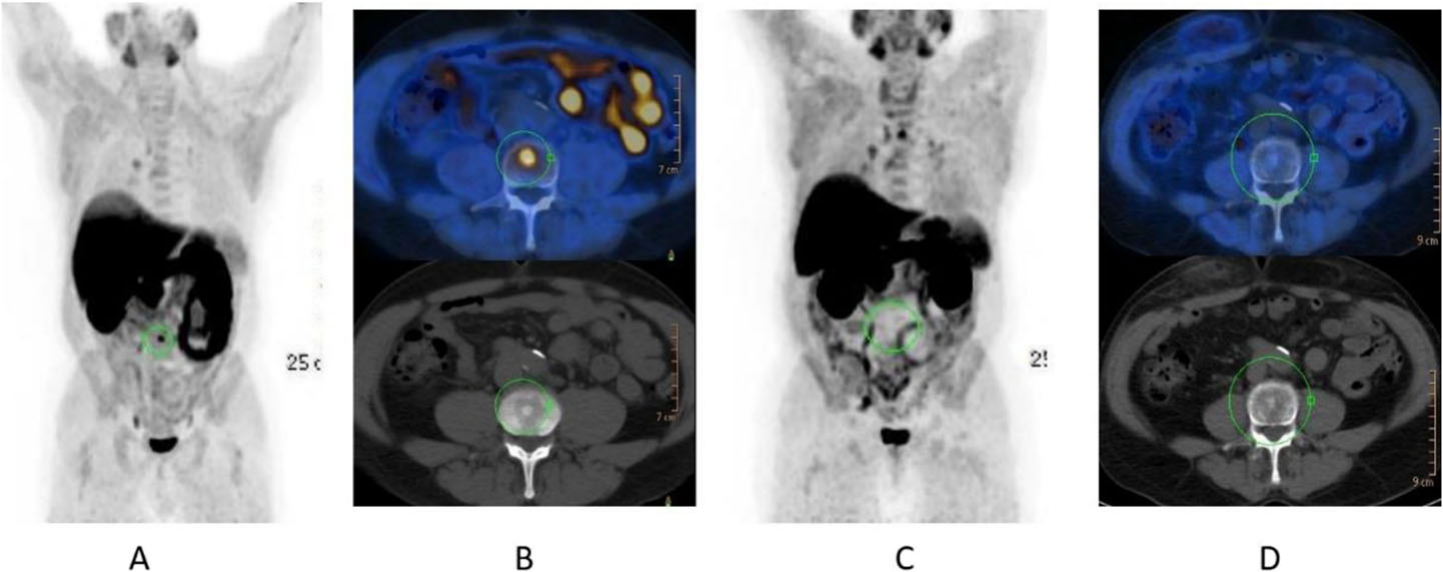
^18^F-fluorocholine, prostate cancer, response evaluation. *Clinical history*: 73 y.o. man with prostate cancer, Gleason score 4 + 3, treated with radical prostatectomy. BCR with PSA 2.3 ng/ml PSAdt 6 months; evaluation after 2 months of hormonal treatment (PSA 0.5 ng/ml). *PET/CT findings*: intense ^18^F-Choline uptake in the vertebra before therapy (**a** MIP; **b** fused and CT) not visible after 2 months of hormonal treatment (**c** MIP; **d** fused and CT)

Clinical indications in oncology (Figs. 20, 21, 22, 23, and 24)

The main clinical application of choline is in prostate cancer patients for staging and restaging the disease in case of biochemical recurrence after primary treatment (Kryza et al. 2008; Evangelista et al. 2013).

### PSMA

Names: [^68^Ga] prostate-specific membrane antigen ligand; ^68^Ga-PSMA

**Fig. 25:**
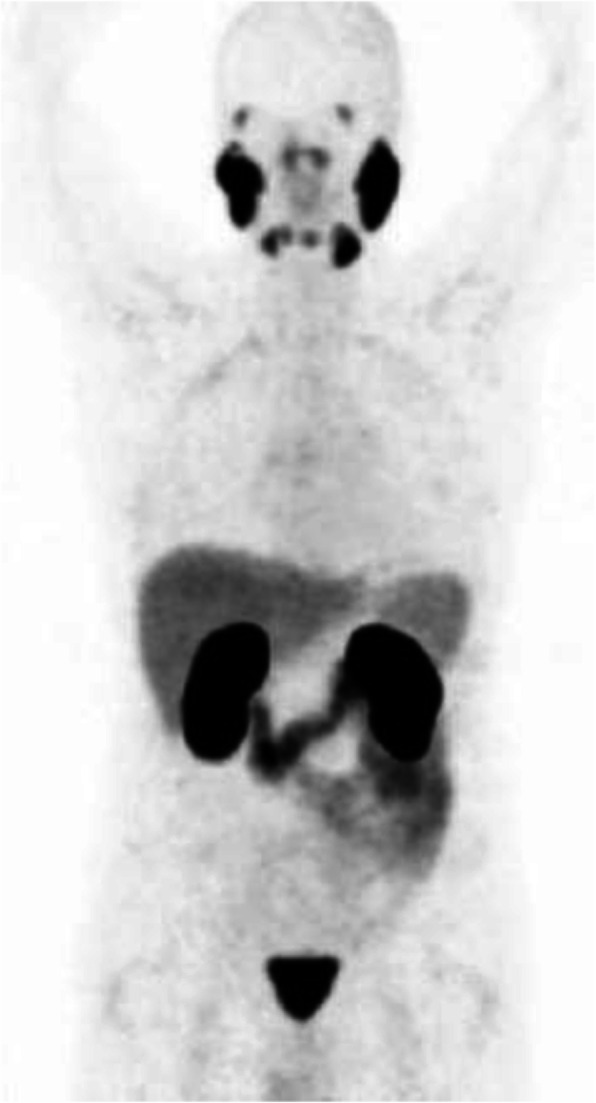
Physiological biodistribution of ^68^Ga-PSMA (PSMA 11). Salivary glands, small bowel (jejunum), kidneys, spleen, and liver are the organs with the highest uptake of ^68^Ga-PSMA-11

Biodistribution and metabolism (Fig. 25)

Prostate specific membrane antigen (PSMA), a tumour-associated antigen and type II transmembrane protein, is expressed on the membrane of prostatic epithelial cells and overexpressed on prostate tumour cells. Upon internalisation of the radiotracer, PSMA-expressing tumour cells can be detected during PET imaging (Heidenreich et al. 2014; Afshar-Oromieh et al. 2016; Demirci et al. 2016).

Scan acquisition

• Fasting of 4 h is suggested

• 2 or 3 MBq\Kg of ^68^Ga-PSMA iv

• Uptake time 60–100 min

• Acquisition starts from the pelvis

Clinical indications in oncology (Figs. 26, 27, 28, 29, 30, 31, 32, 33, 34, 35, 36, and 37)

**Fig. 26:**
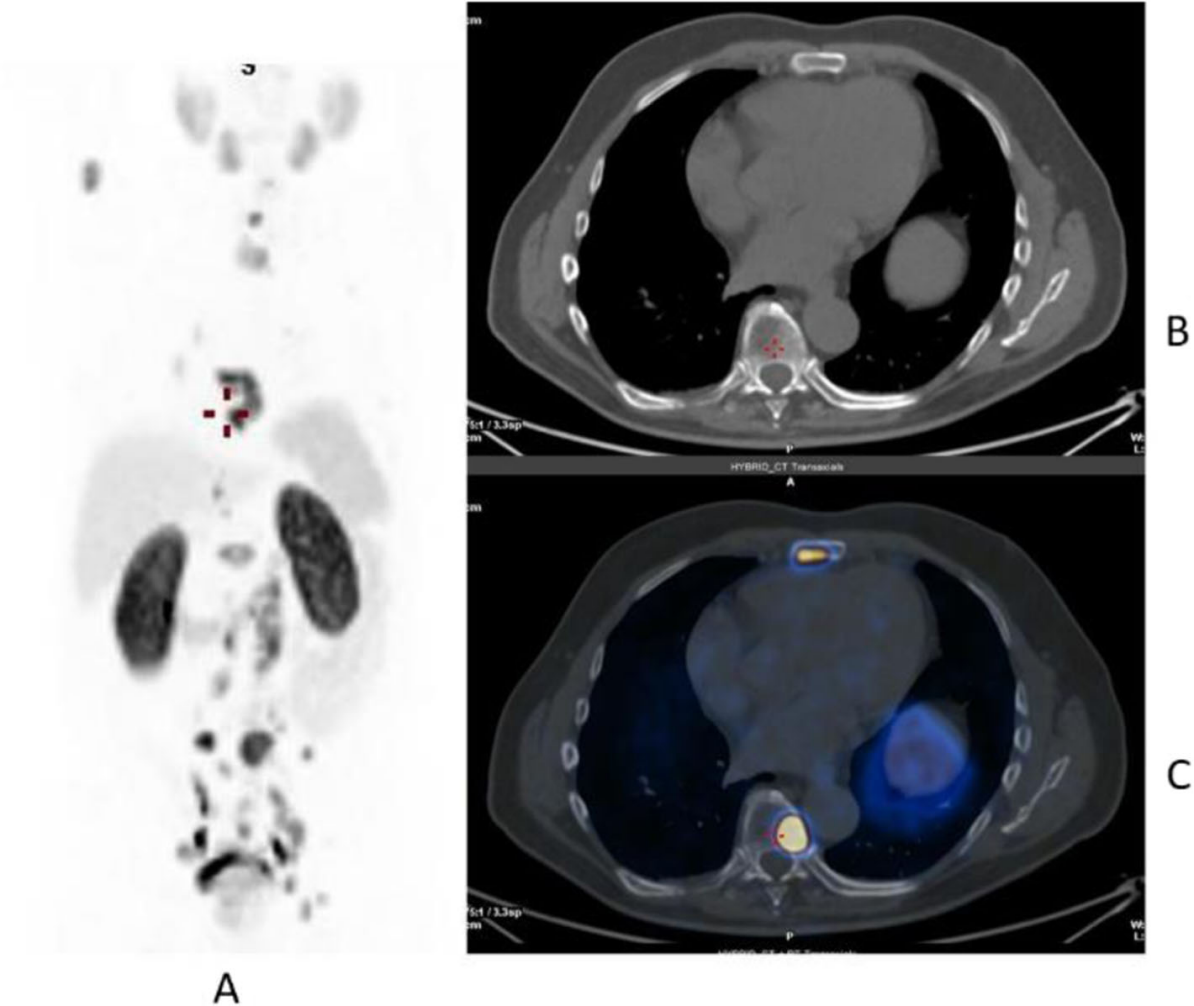
^68^Ga-PSMA, prostate cancer, staging. *Clinical history*: 56 y.o. man with prostate cancer. At presentation, Gleason score 4 + 5; PSA = 14 ng/ml, candidate to radical prostatectomy. *PET/CT findings*: multiple foci of increased uptake involving prostate, lymph nodes, and bones (**a** MIP). Vertebral lesion seen on fused images (**c**) is not evident on CT (**b**)

**Fig. 27:**
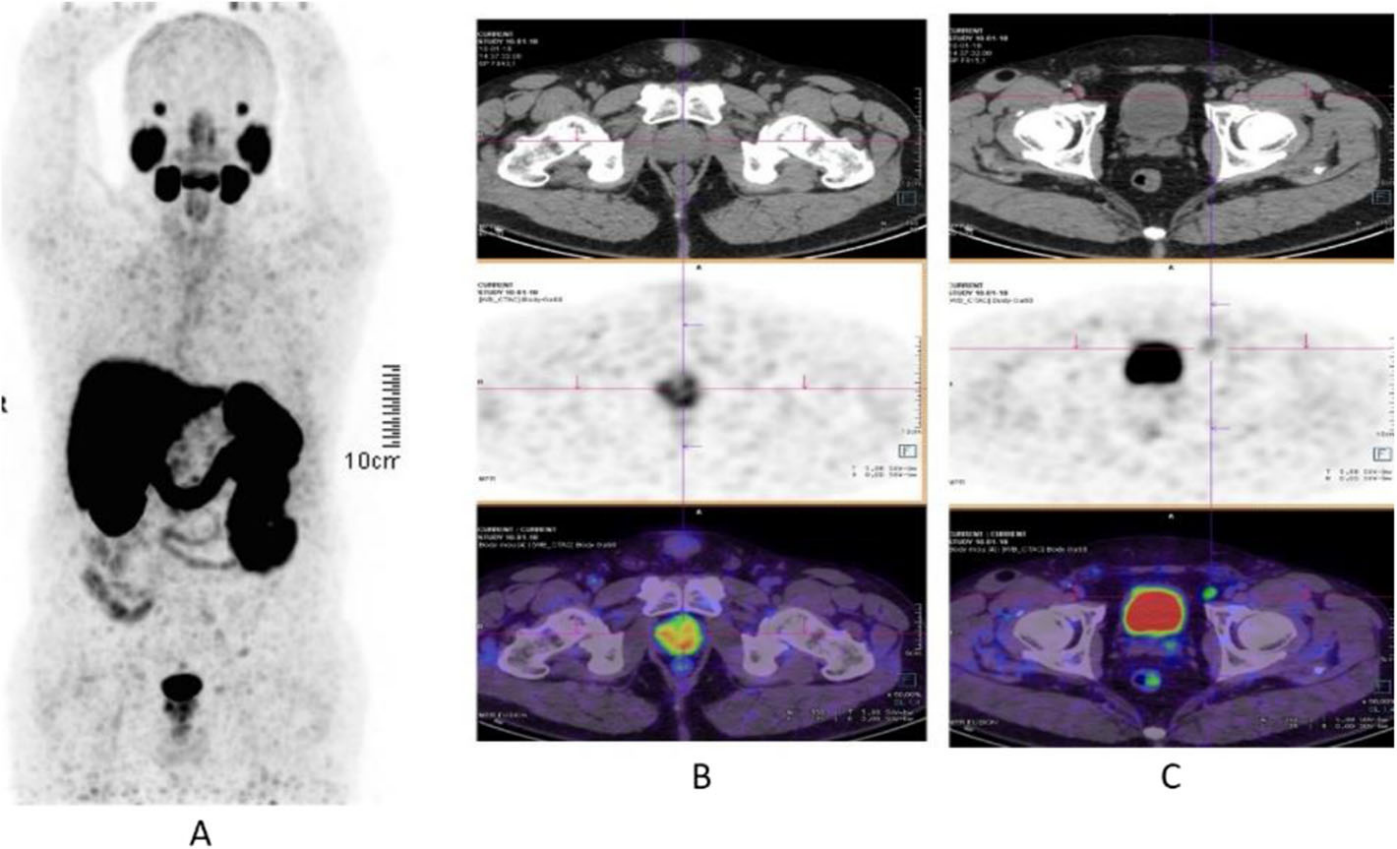
68Ga-PSMA, prostate cancer, initial staging. *Clinical history*: 58 y.o. man with prostate cancer, Gleason score 3 + 4. Suspicious rib lesion on bone SPECT/CT. *PET/CT findings*: increased uptake in prostate tumour. No abnormal findings in the skeleton. Faint uptake in inguinal and external iliac nodes, with abnormal appearance on CT, corresponding to inflammation. **a** MIP; **b**, **c** fused images

**Fig. 28:**
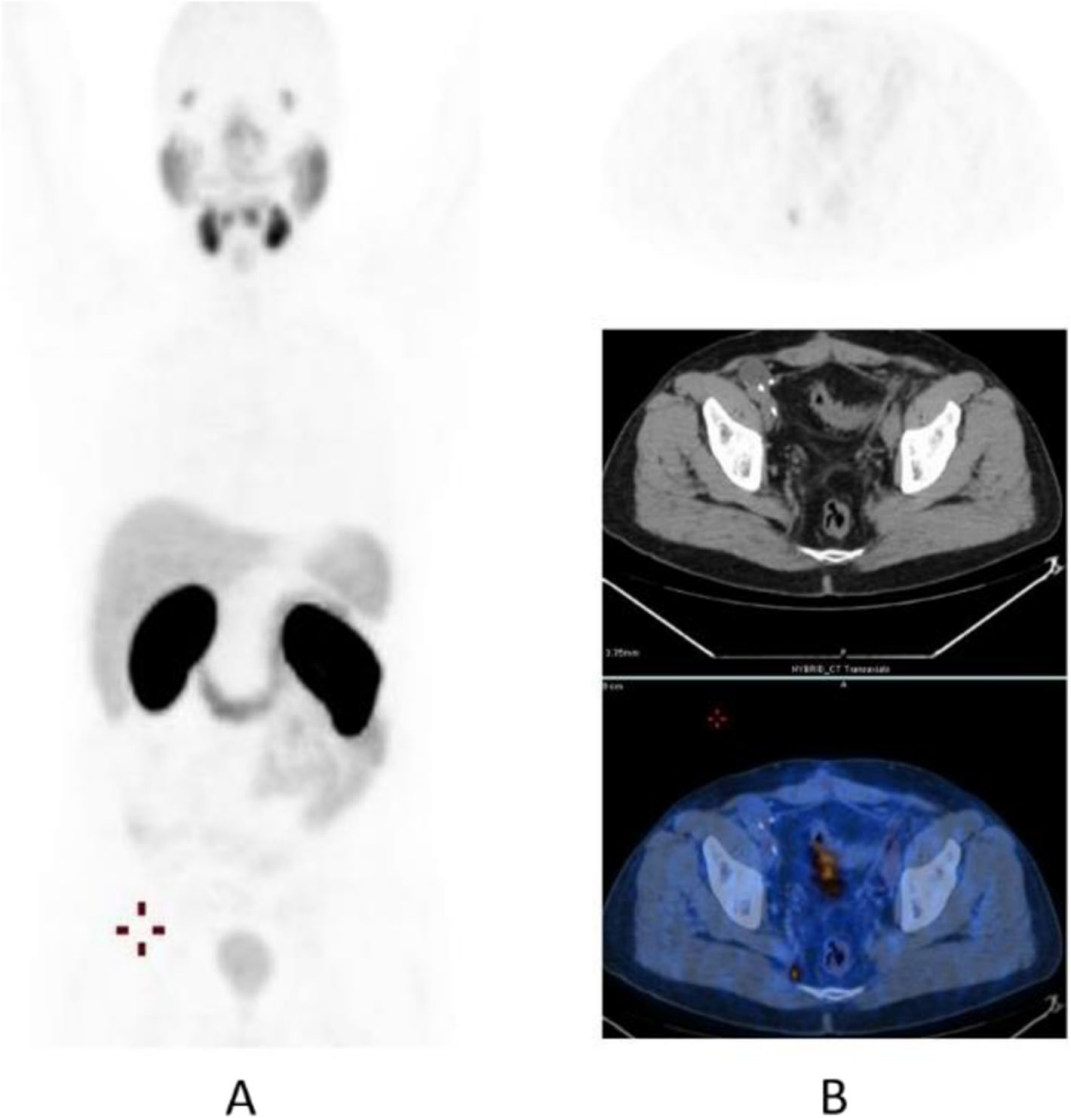
^68^Ga-PSMA, prostate cancer, biochemical recurrence (BCR): sensitivity. *Clinical history*: 68 y.o. man with prostate cancer, Gleason score 4 + 3, radical prostatectomy as primary treatment; PSA 0.8 ng/ml, PSAdt 5 months, TTR 24 months, candidate for salvage radiation therapy to the prostatic fossa. *PET/CT findings*: single focus of faint uptake seen in the internal iliac chain, corresponding to a small right presacral nodule at CT image. **a** MIP. **b** PET, CT, and fused images

**Fig. 29:**
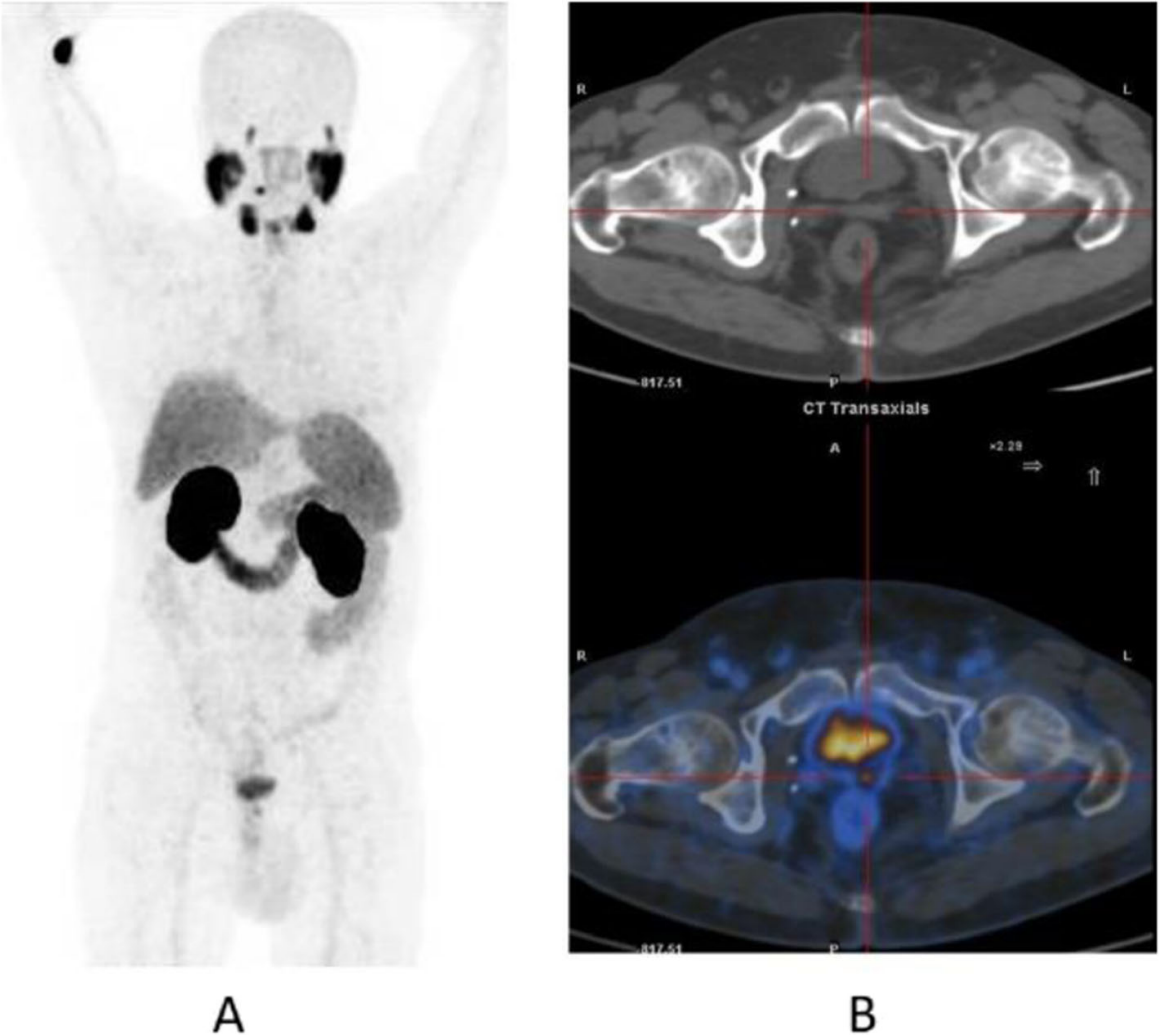
^68^Ga-PSMA, prostate cancer, biochemical recurrence (BCR). *Clinical history*: 62 y.o. man with prostate cancer, radical prostatectomy as primary treatment Gleason score 4 + 4; PSA = 0.7 ng/ml, PSAdt 5 months, TTR 24 months, candidate to salvage radiation therapy to the prostatic fossa. *PET/CT findings*: single focus of increased uptake seen in the prostatic fossa consistent with local relapse after biopsy. **a** MIP; **b** CT and fused images

**Fig. 30:**
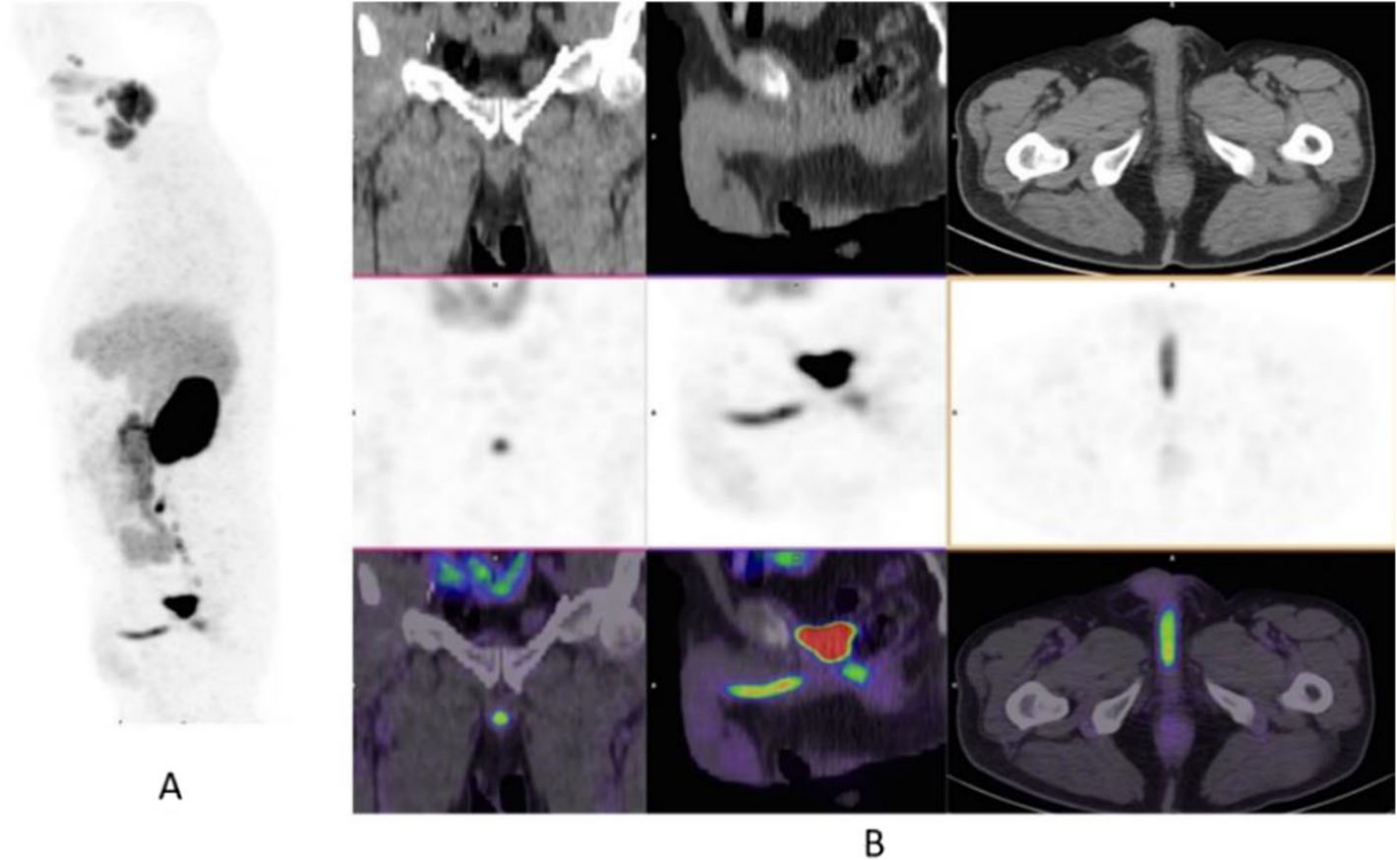
^68^Ga-PSMA, prostate cancer, biochemical recurrence (BCR): specificity. *Clinical history*: 68 y.o. man with prostate cancer, prostatectomy as primary treatment, Gleason score 4 + 5; pT3N0Mx + adjuvant radiotherapy. Three years later: PSA = 2.49 ng/ml, PSAdt 8 months. *PET/CT findings*: no evidence of recurrent disease, but urinary activity is evident in the prostatic fossa and the urethra. **a** MIP in a lateral view; **b** CT, PET, and fused images

**Fig. 31:**
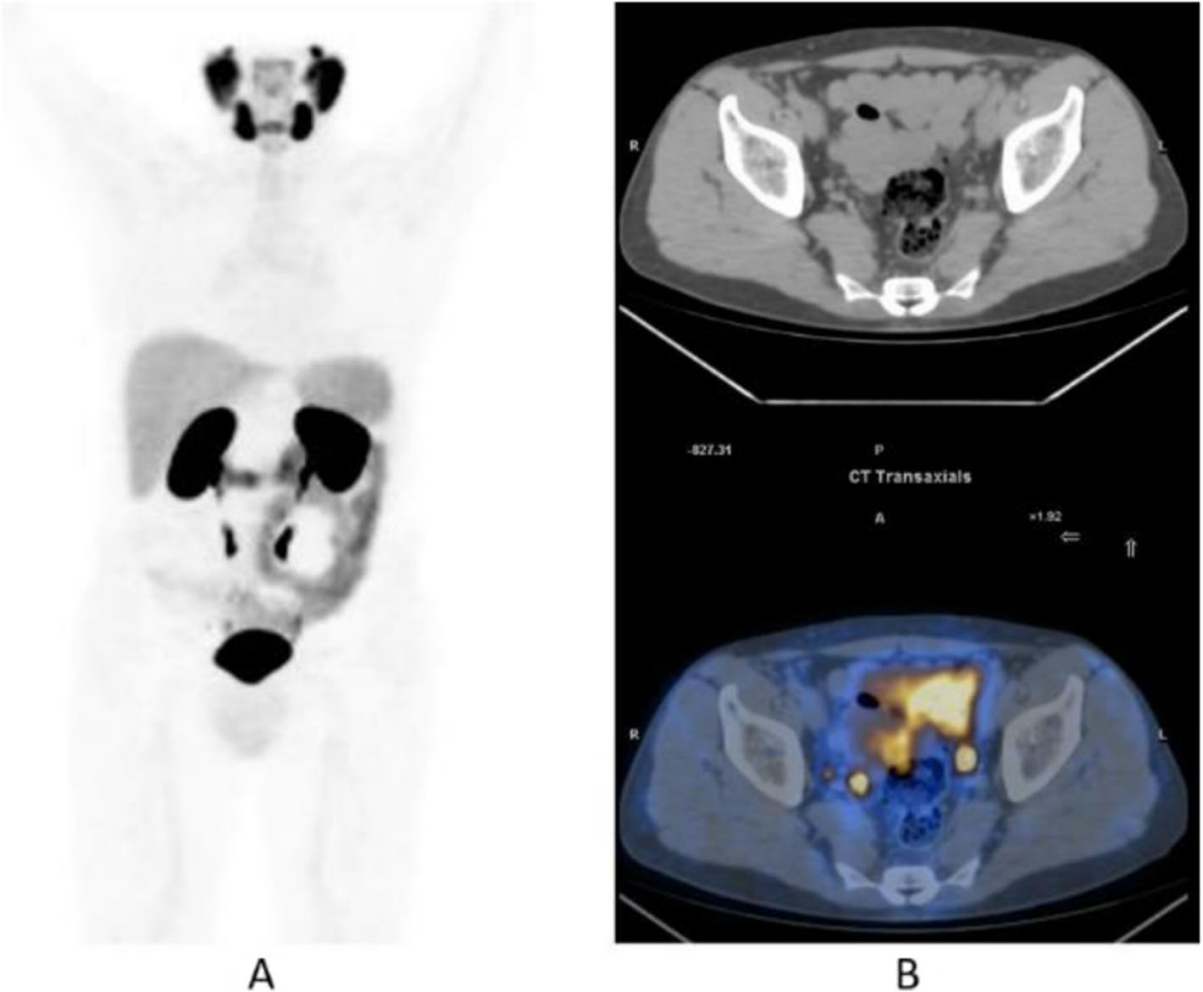
^68^Ga-PSMA, prostate cancer, biochemical recurrence (BCR): sensitivity. *Clinical history*: 64 y.o. man with prostate cancer, radical prostatectomy as primary treatment Gleason score 4 + 4; PSA = 0.7 ng/ml, PSA dt 6 months, TTR 12 months, candidate for salvage radiation therapy in the prostatic fossa. *PET/CT findings*: single focus of increased uptake seen in a right external iliac lymph node (obturatory) measuring 8 mm in maximum diameter. **a** MIP; **b** CT and fused images

**Fig. 32:**
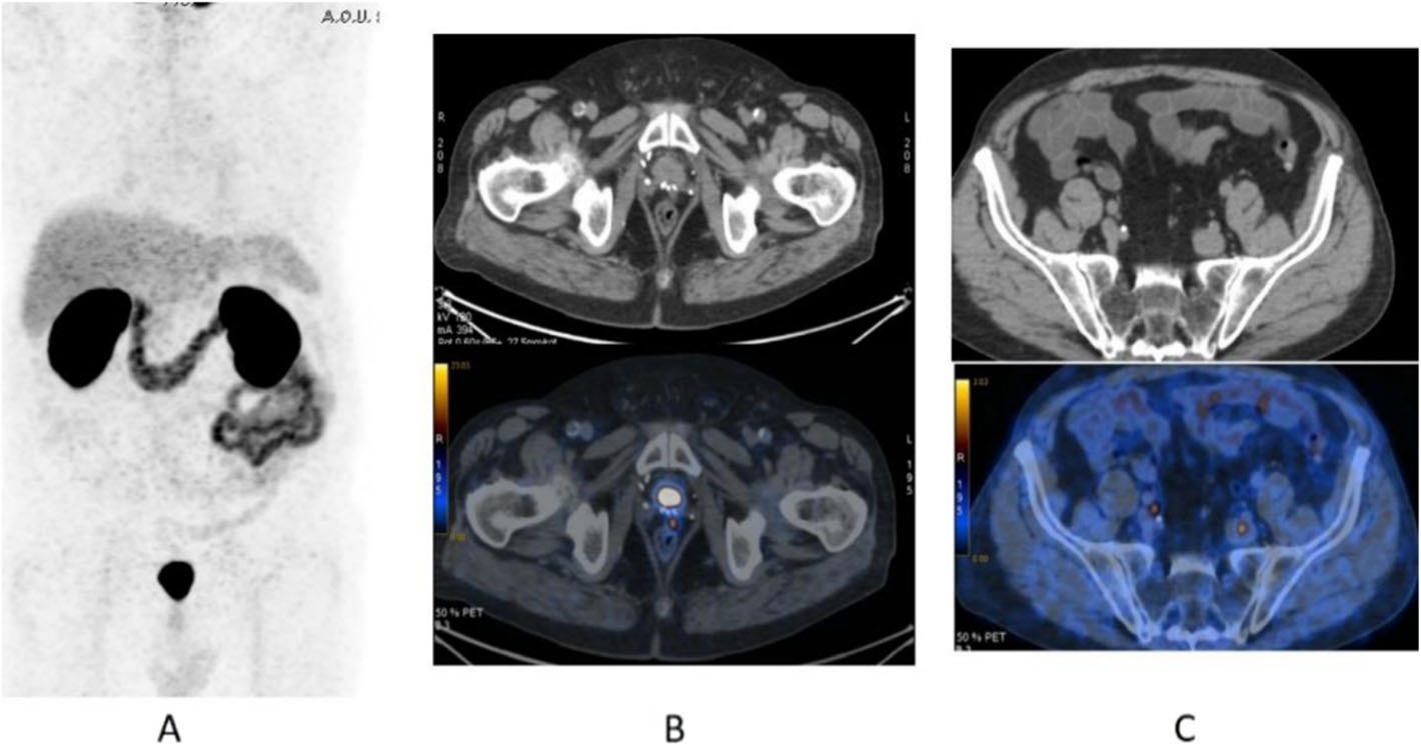
^68^Ga-PSMA, prostate cancer, biochemical recurrence (BCR): sensitivity. *Clinical history*: 72 y.o. man with prostate cancer, radical prostatectomy as primary treatment Gleason score 4 + 5; PSA = 0.4 ng/ml, PSA dt 6 months, TTR 10 months, candidate for salvage radiation therapy in the prostatic fossa. *PET/CT findings*: a focus of increased uptake is seen in the prostatic fossa and a right common iliac lymph node measuring 7 mm in maximum diameter. **a** MIP; **b** CT and fused images, local relapse; **c** CT and fused images, iliac lymph node

**Fig. 33:**
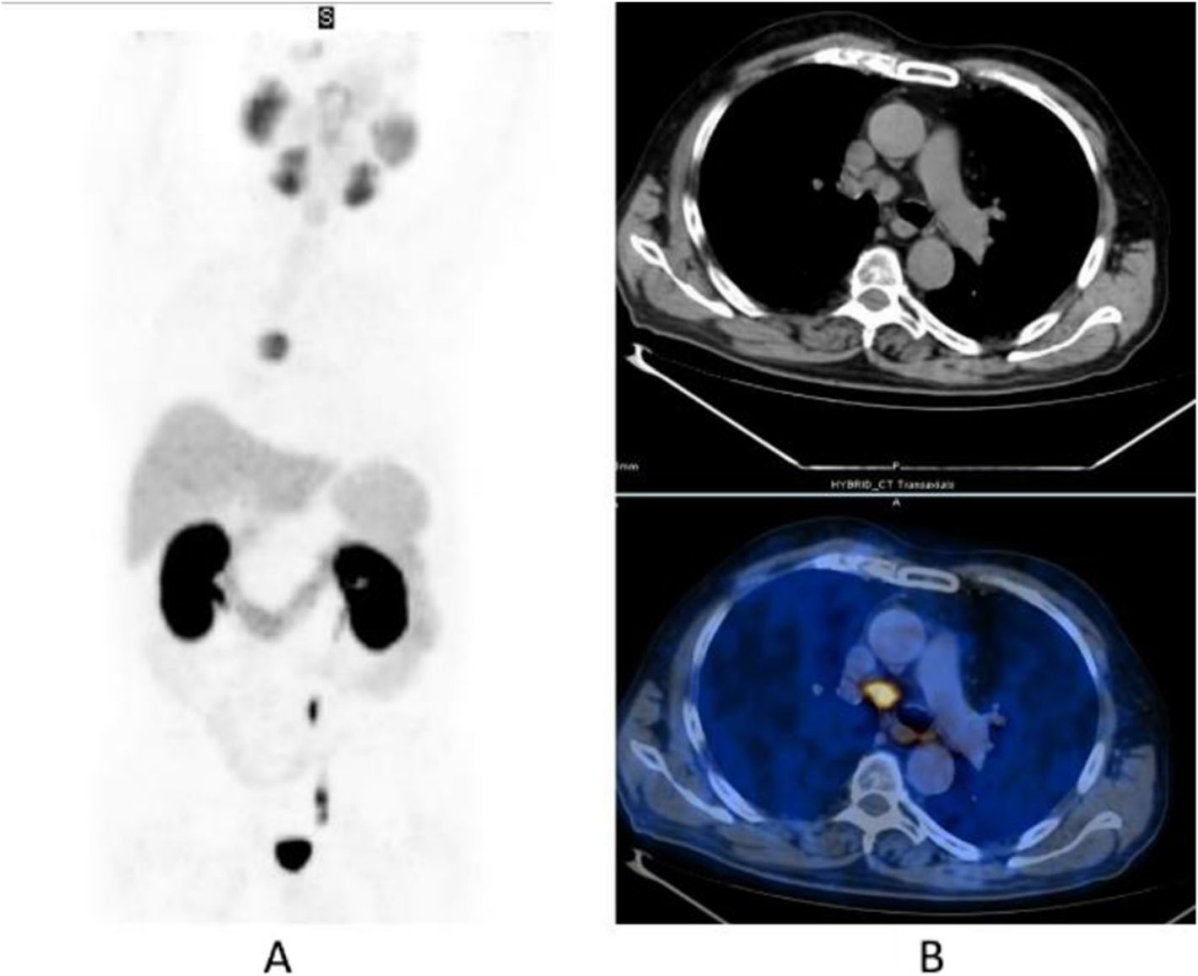
^68^Ga-PSMA, prostate cancer, biochemical recurrence (BCR): uncommon precarinal solitary metastasis *Clinical history*: 64 y.o. man with prostate cancer, radical prostatectomy as primary treatment Gleason score 4 + 5; PSA = 1.9 ng/ml, PSA dt 6 months, TTR 28 months. *PET/CT findings*: focus of increased uptake seen in an enlarged precarinal lymph node (24 mm). Transbronchial biopsy diagnosed prostate cancer relapse. **a** MIP images; **b** CT and fused images

**Fig. 34:**
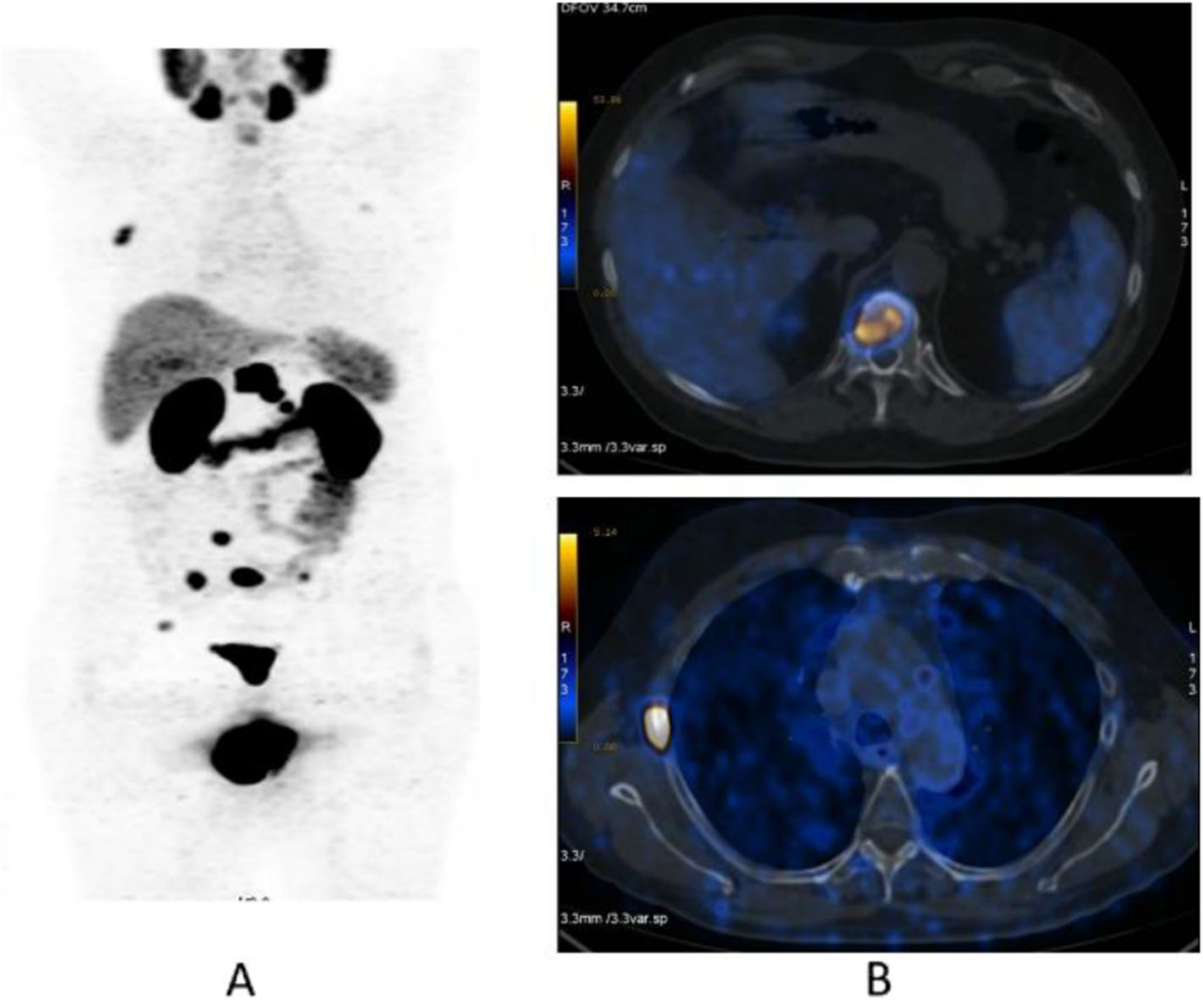
^68^Ga-PSMA, prostate cancer, biochemical recurrence (BCR): 223Ra feasability. *Clinical history*: 72 y.o. man with prostate cancer, radical prostatectomy as primary treatment Gleason score 4 + 3; during abiraterone treatment, PSA increased up to 127 ng/ml, candidate to 223Ra chloride. *PET/CT findings*: multiple foci of increased uptake in the bones. No lymph node or visceral metastases. After ^68^Ga-PSMA PET/CT, the patient has been referred to 223Ra chloride treatment. **a** MIP; **b** fused images

**Fig. 35:**
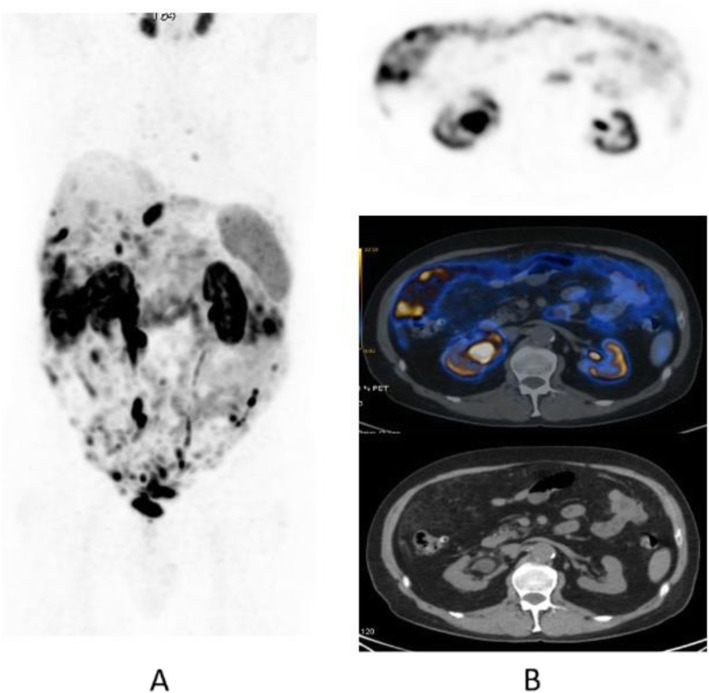
^68^Ga-PSMA, prostate cancer, biochemical recurrence (BCR): peritoneal invasion. *Clinical history*: 67 y.o. man with prostate cancer, radical prostatectomy as primary treatment Gleason score 4 + 3; during anti-androgen treatment, PSA increased up to PSA = 114 ng/ml. *PET/CT findings*: multiple foci of increased uptake in the peritoneum, liver capsule, and mediastinal lymph nodes. **a** MIP. **b** PET, fused, and CT images

**Fig. 36:**
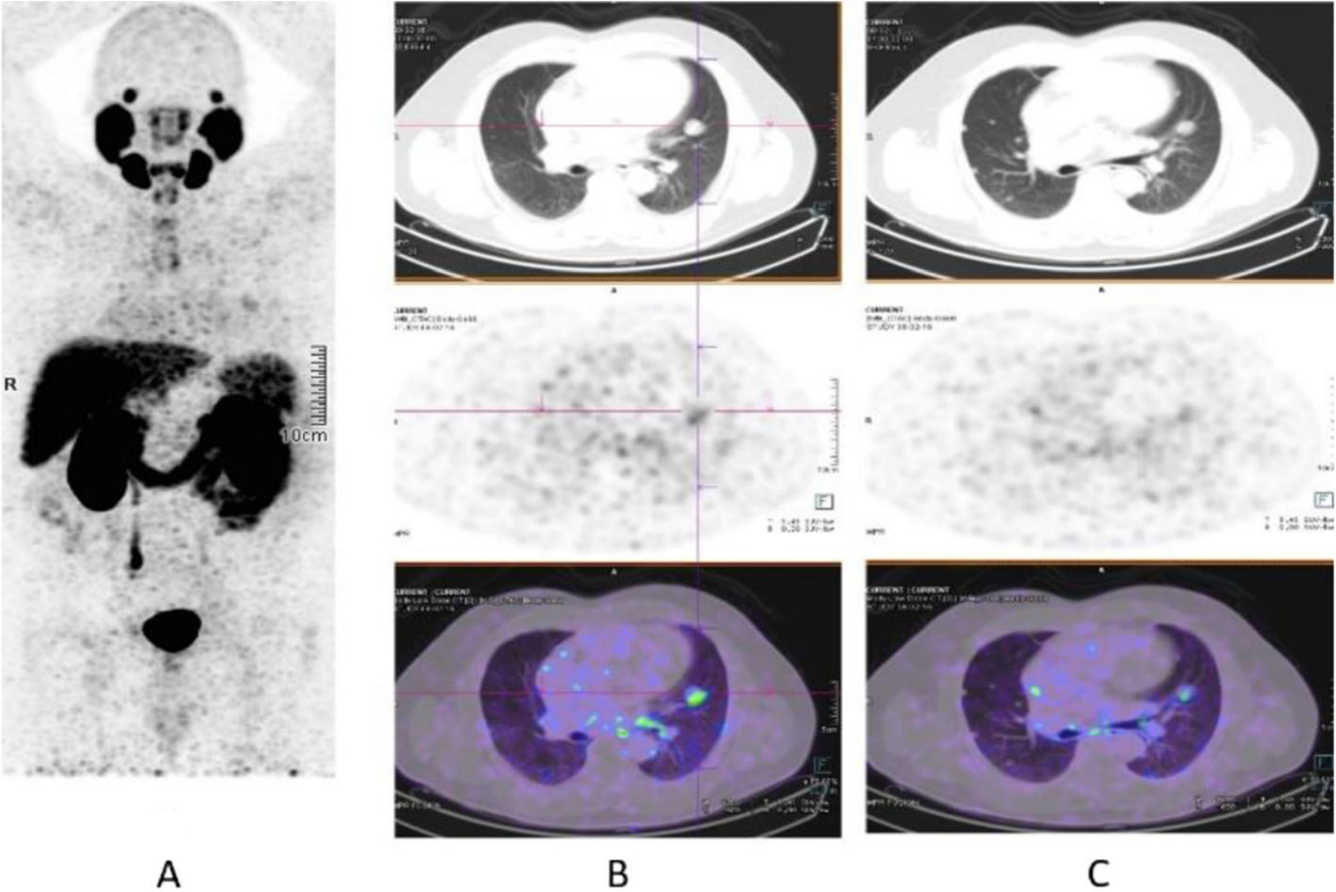
^68^Ga-PSMA, prostate cancer, biochemical recurrence (BCR): pulmonary metastasis. *Clinical history*: 66 y.o. man with prostate cancer (Gleason score 4 + 3, pT3N0Mx), radical prostatectomy, and adjuvant RT. Three years later PSA = 1.62 ng/ml, PSAdt < 6 months. Bone scan, abdominal CT, and pelvic MRI were all negative. *PET/CT findings*: mild tracer uptake in multiple pulmonary nodules visualised on CT, the largest nodule is 19 mm, in the lingula. **a** MIP; **b**, **c** CT, PET, and fused images

**Fig. 37:**
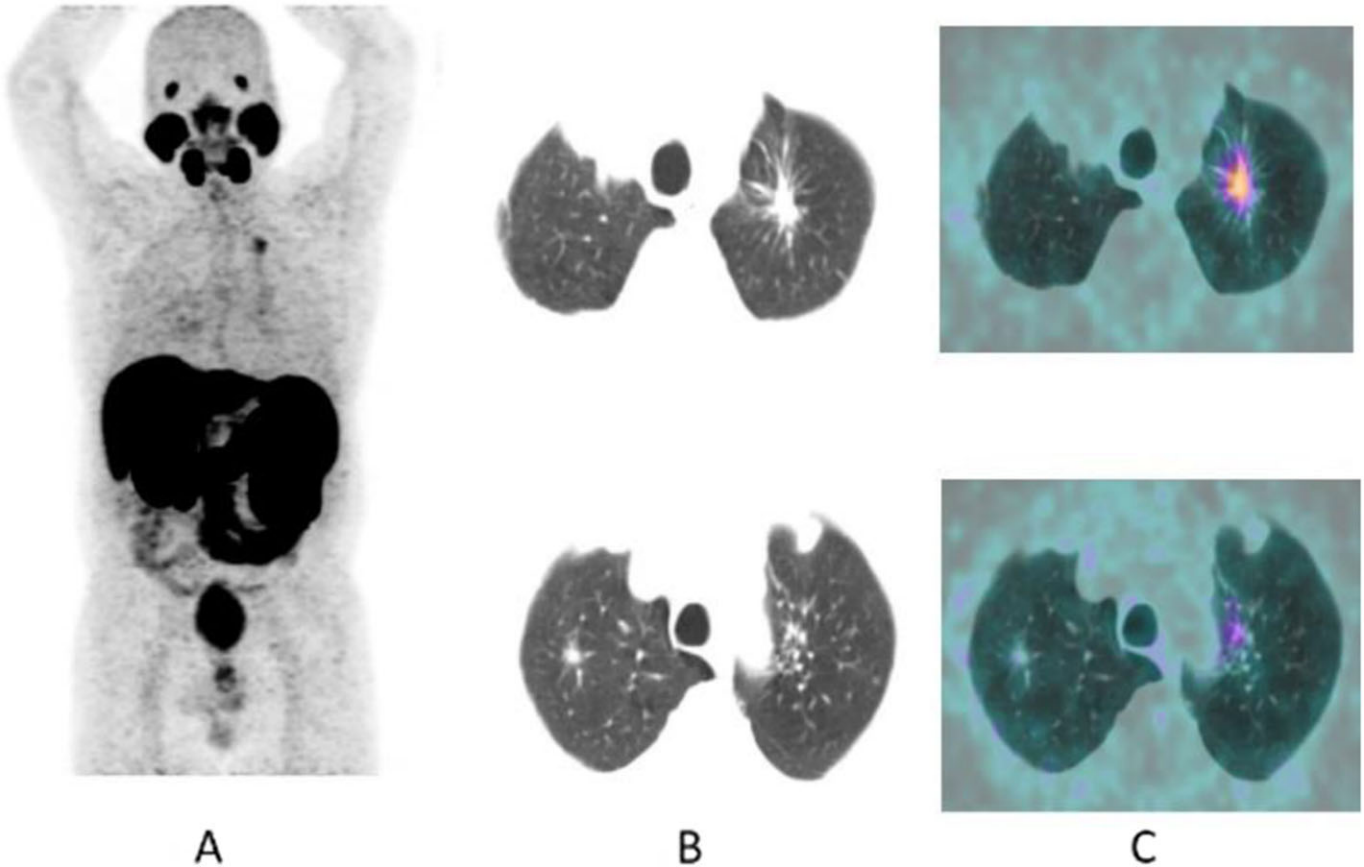
^68^Ga-PSMA, prostate cancer, initial staging: lung lesion. *Clinical history*: 68 y.o. patient diagnosed with prostate cancer, treated with radical prostatectomy. First biochemical recurrence: PSA 0.39 ng/ml. *PET/CT findings*: increased uptake in a pulmonary nodule with irregular margins and spiculated edges in the left upper lobe. Biopsy diagnosed a primary lung adenocarcinoma. **a** MIP, **b** CT images, **c** fused images

The main clinical application of ^68^Ga-PSMA is in prostate cancer patients, namely initial diagnosis (Fendler et al. 2017), nodal staging (Schneider et al. 2016), restaging in case of biochemical recurrence (Calais et al. 2018; Maurer et al. 2016), and theranostic in case of ^177^Lu-PSMA treatment (Mottet et al. 2011; Zamboglou et al. 2016), or alfa emitters such as ^225^AcPSMA (Maurer et al. 2016).

### DOPA

Names: L-3,4-Dihydroxy-6-[^18^F] fluorophenylalanine, ^18^F-DOPA, ^18^F-Fluoro-L-DOPA

**Fig. 38:**
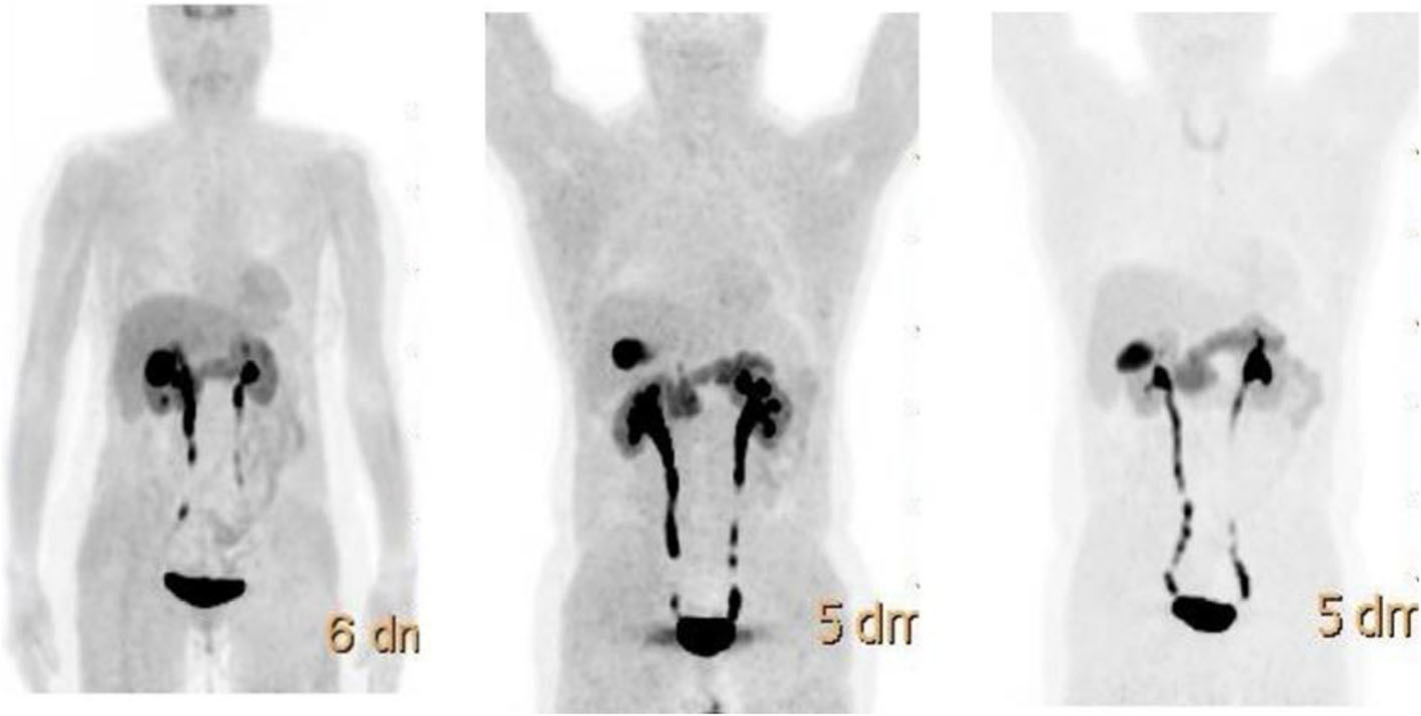
Physiological biodistribution of ^18^F-FDOPA

Biodistribution and metabolism (Fig. 38)

^18^F-DOPA reflects all stages of DOPA transport, storage, and metabolism. The tracer is metabolised in the striatum, but also in peripheral tissues such as liver, kidneys, and lung (Rahbar et al. 2017).

Scan acquisition

• Fasting for more than 4 h

• 2–3 MBq/Kg of ^18^F-DOPA iv

• Uptake time 60 min for extra-cranial tumours. An additional acquisition of 10 min after injection is suggested in medullary thyroid cancer.

• Uptake time 10 min for primary brain tumours.

**Fig. 39:**
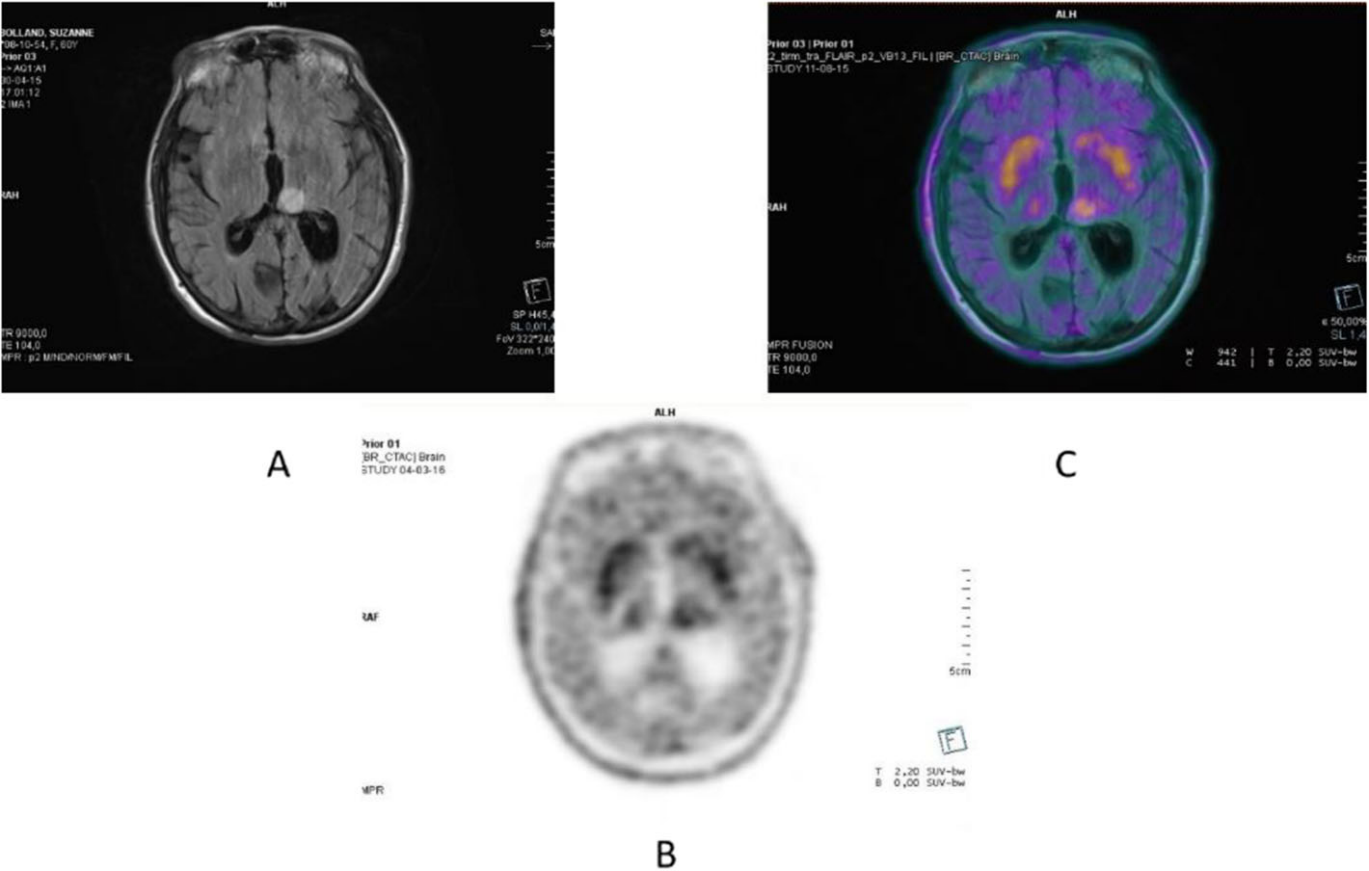
^18^F-FDOPA, glioma. *Clinical history*: 61 y.o. woman with indeterminate left thalamic lesion. MRI consistent with low grade glioma (**a**). *PET/CT findings*: mild ^18^F-FDOPA uptake in the lesion, also consistent with low grade glioma (**b** PET, **c** fused PET and MR). Follow-up (2 years): no evolution

**Fig. 40:**
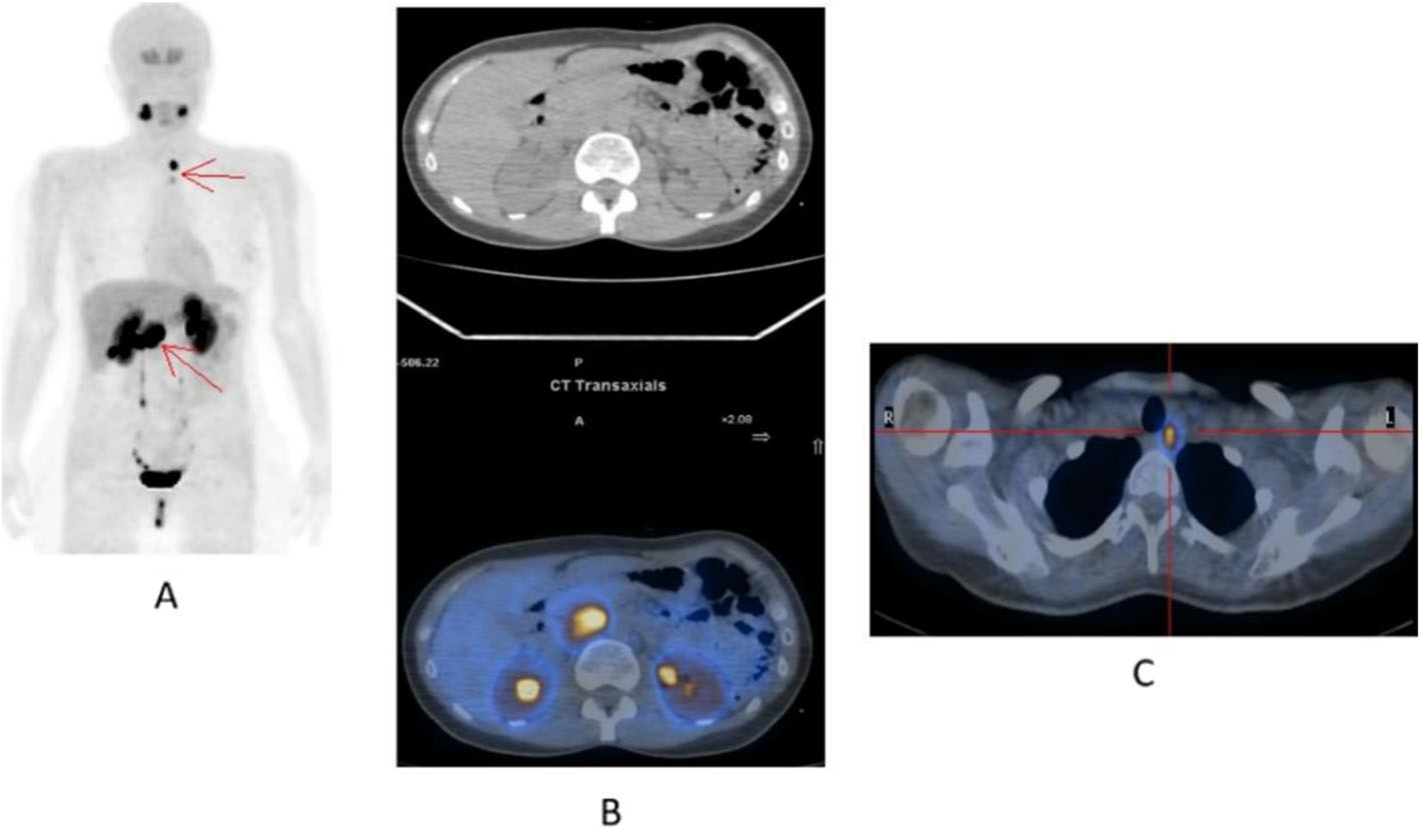
^18^F-FDOPA, paraganglioma. *Clinical history*: 48 y.o. man with suspected abdominal paraganglioma. *PET/CT findings*: intense uptake of the tracer in the abdominal paraganglioma and in a left paratracheal lesion showed in MIP (**a**), CT and fused images (**b** and **c**). Surgery confirmed two paraganglioma lesions

**Fig. 41:**
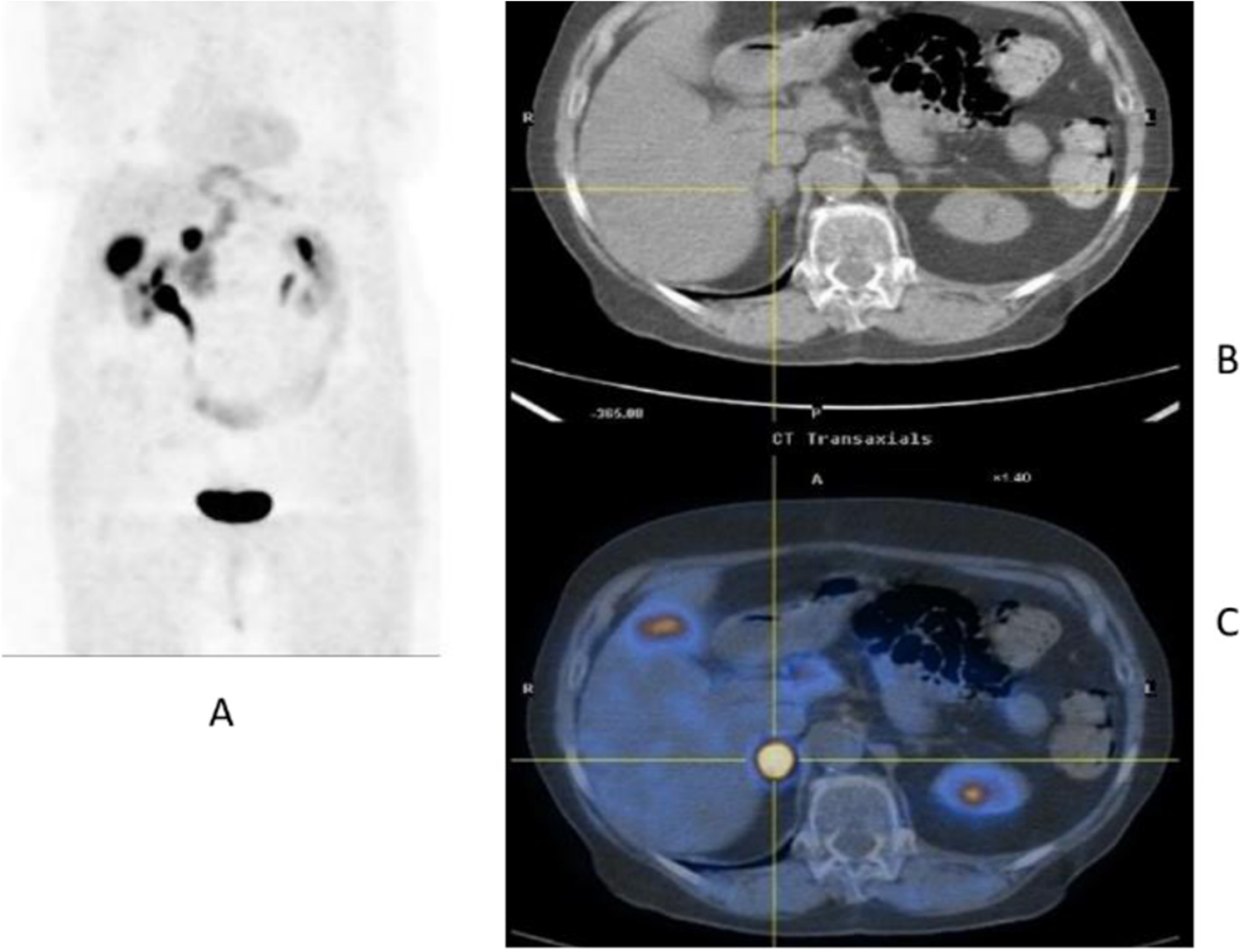
^18^F-FDOPA, pheochromocytoma. *Clinical history*: 45 y.o. man with resistant hypertension. Clinical suspicion of phaeochromocytoma. *PET/CT findings*: intense uptake of the tracer in the right adrenal gland in MIP (**a**), which is enlarged on CT (**b**) and fused images (**c**). Surgery confirmed presence of phaeochromocytoma

**Fig. 42:**
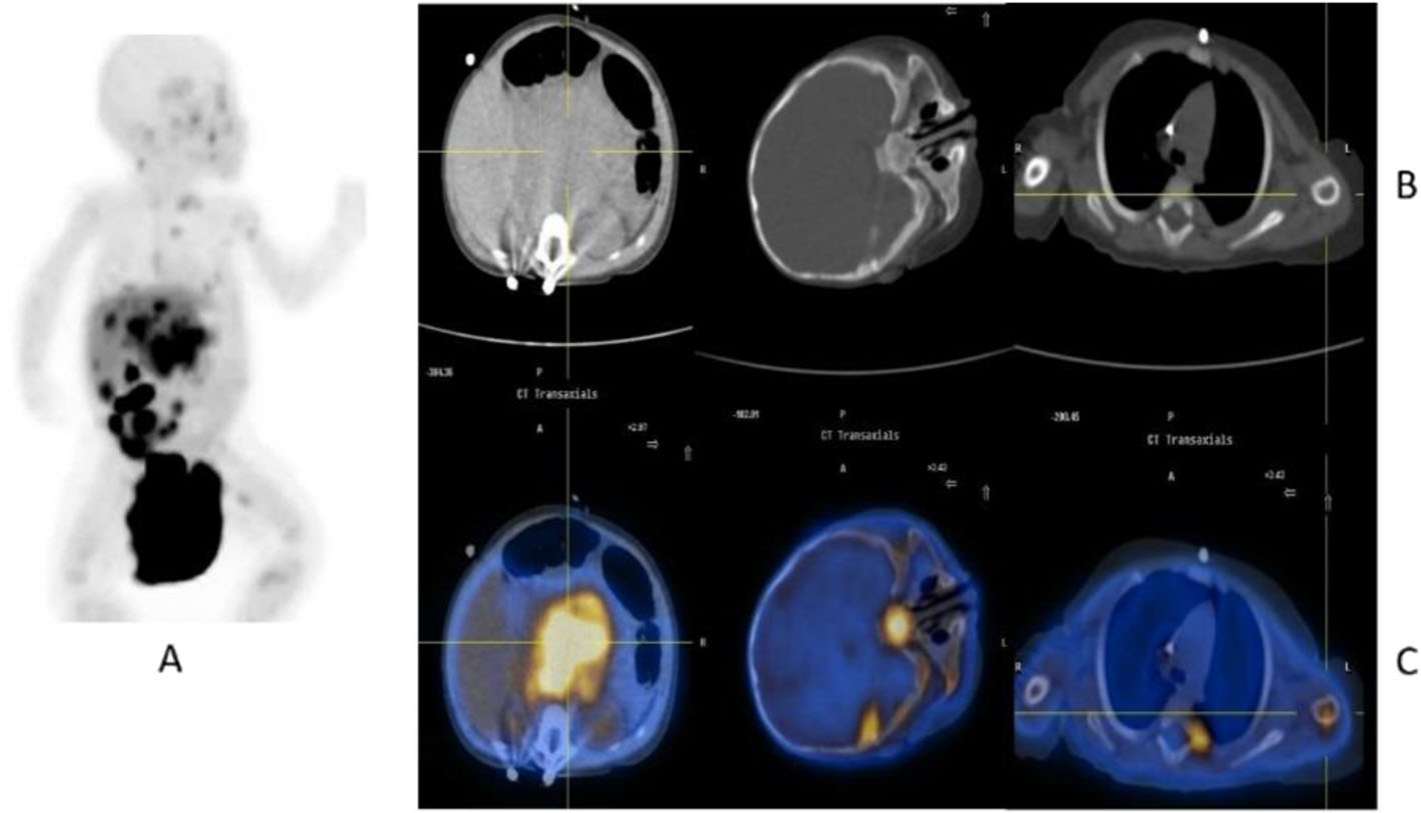
^18^F-FDOPA, neuroblastoma. *Clinical history*: 6 months old girl with neuroblastoma, stage IV at presentation. *PET/CT findings*: intense uptake of the tracer in multiple metastases in the liver and bones, in particular sphenoid and left humerus, seen on MIP (**a**), CT (**b**), and fused images (**c**)

**Fig. 43:**
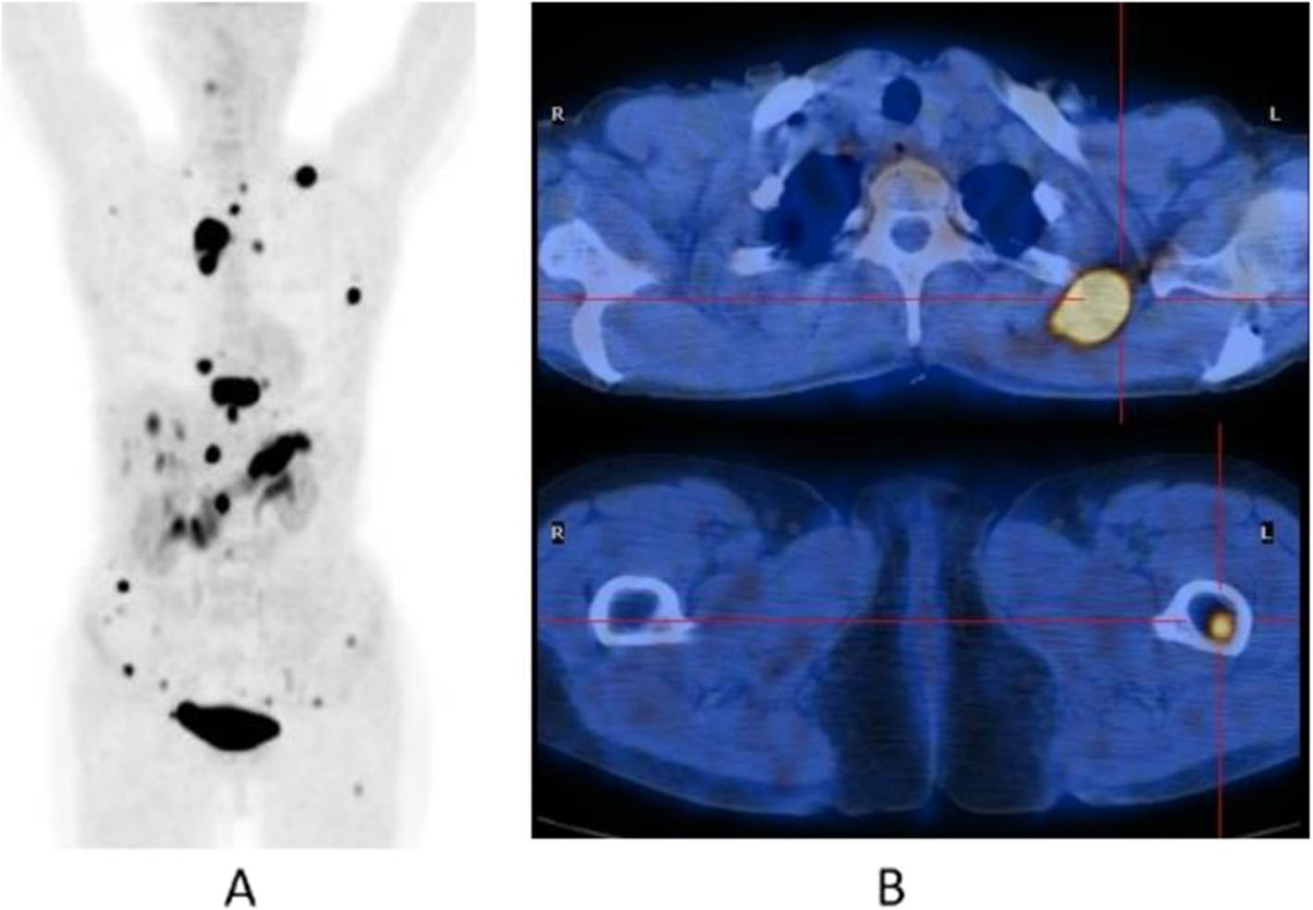
^18^F-FDOPA, carcinoid. *Clinical history*: 45 y.o. man with suspected right lung carcinoid. *PET/CT findings*: intense tracer uptake in the right lung and in multiple bone and soft tissue lesions seen on MIP (**a**) and fused images (**b**)

Clinical indications in oncology (Fig. 39, 40, 41, 42, and 43)

^18^F-DOPA is used in the detection of neuroendocrine tumours. It is the PET tracer of choice for recurrence detection in patients with medullary thyroid cancer and may play a role in the management of patients with pheochromocytoma and neuroblastoma. ^18^F-DOPA PET/CT is also used in recurrent glioma (Kratochwil et al. 2017; Chondrogiannis et al. 2013; Soussan et al. 2012; Amodru et al. 2018).

### 5-HTP

Names: [^11^C] 5-hydroxytryptophan; ^11^C-HTP

**Fig. 44:**
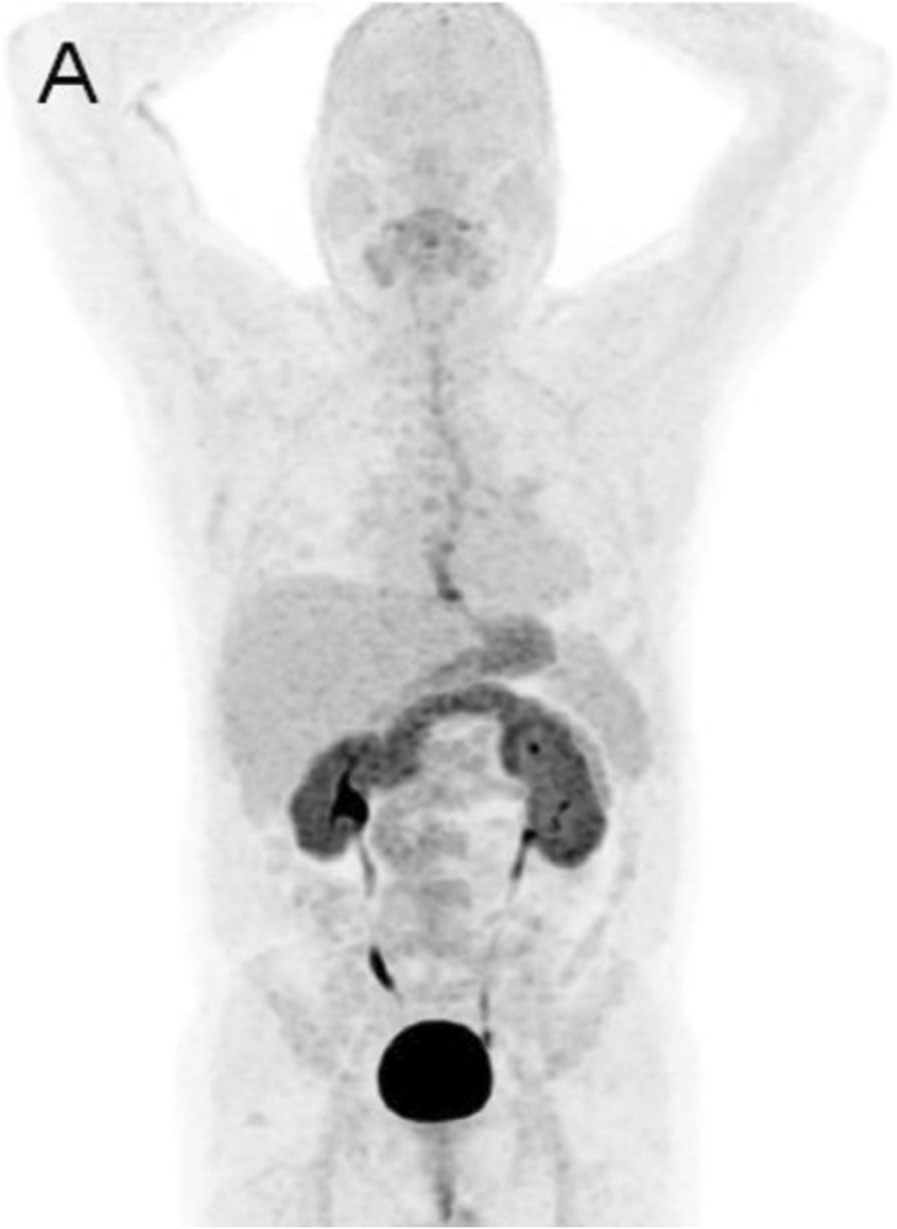
Physiological biodistribution of ^11^C 5-HTP

Biodistribution and metabolism (Fig. 44)

^11^C-HTP is taken up into neuroendocrine tumours cells by L-large amino acid transporter followed by decarboxylation to serotonin. The resulting end-product is then transported into storage vesicles through the vesicular monoamine transporter as well as went through the metabolic pathway of serotonin (Addeo et al. 2018; Piccardo et al. 2012).

Scan acquisition

• No special diet is required

• 370 MBq of ^11^C-HTP iv

• Uptake time 1 h

**Fig. 45:**
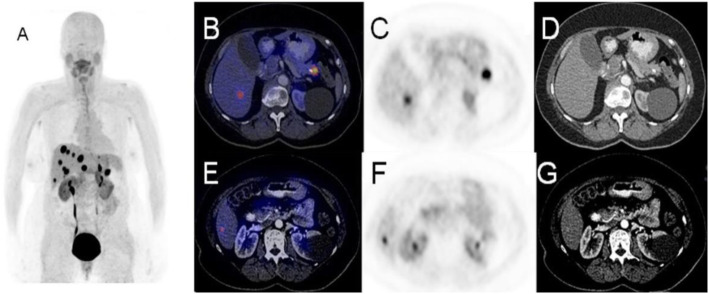
^11^C 5-HTP NET of the pancreas, staging. *Clinical history*: 65 y.o. woman with pancreatic NET, metastatic to liver. *PET/CT findings*: MIP (**a**) showing multiple sites of intense tracer accumulation in the upper abdomen. Fused images of PET with diagnostic CT (**b**, **e**), PET only (**c**, **f**), and CT only (**d**, **g**) showing intense tracer accumulation in a lesion in the cauda of the pancreas, with vague contrast enhancement and a calcification on the CT as well as in liver metastases (**b**–**d**)

Clinical indications in oncology (Fig. 45)

^11^C-HTP is used in the detection of neuroendocrine tumours. Since the uptake is related to the serotonergic pathway, ^11^C-HTP is a possible alternative to ^68^Ga-DOTA-peptide or ^18^F-DOPA (Neels et al. 2006).

### Somatostatin analogues

Names: [^68^Ga] (1,4,7,10-tetraazacyclododecane-N, N′, N″, N‴-tetraacetic acid)-1- (d)-Phe1-Thy3-octreotate (DOTATATE)- (d)-Phe1-Thy3-octreotide (DOTATOC)- NaI3-octreotide (DOTANOC)

**Fig. 46:**
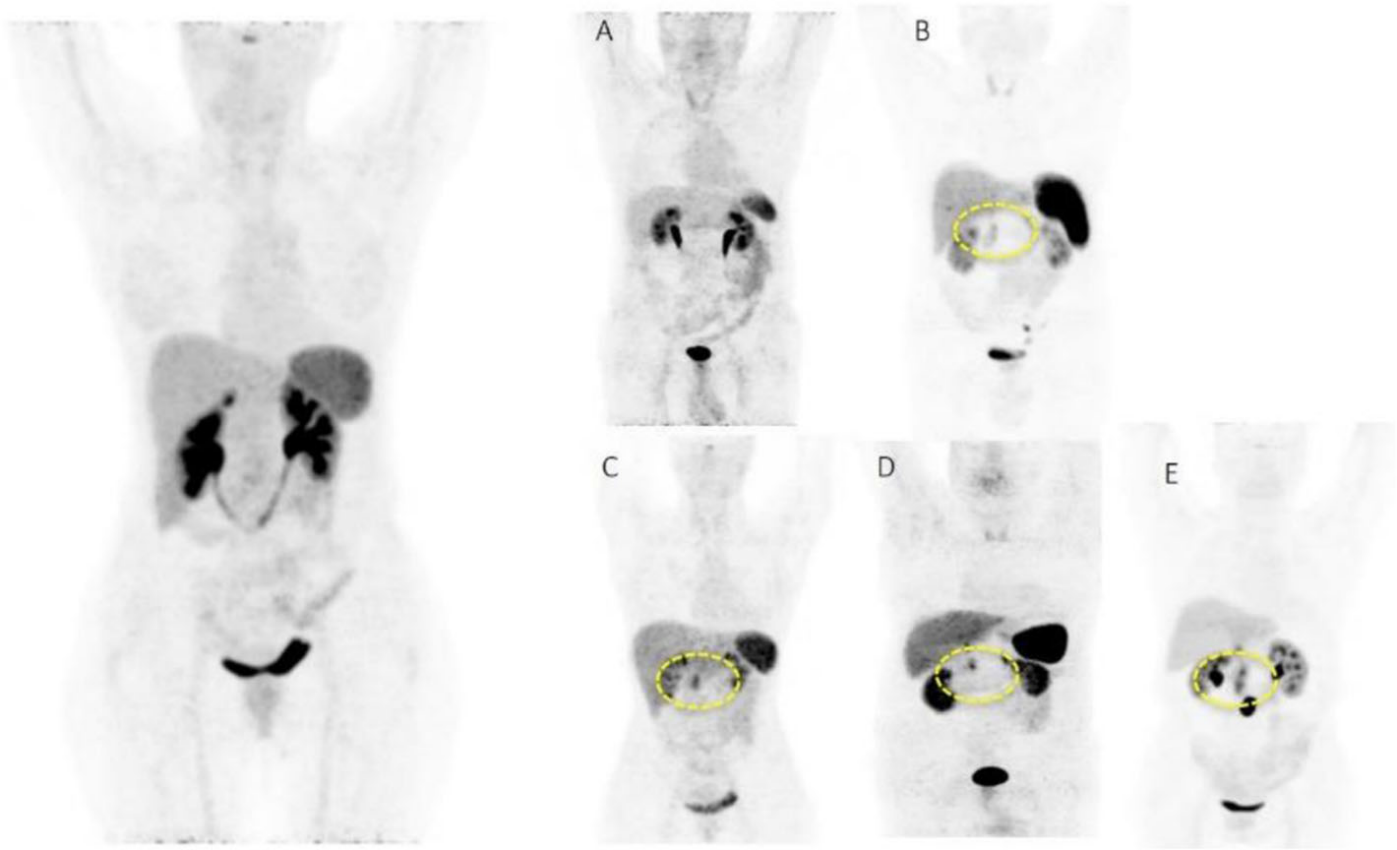
Physiological bio-distribution of 68Ga-DOTA peptide. Spleen and liver are the organs with the higher uptake of ^68^Ga-DOTA peptide. Patterns of increased physiological uptake in the uncinate process of the pancreas (yellow marker): **a** no uptake, **b** mild diffuse uptake, **c** intense diffuse uptake, **d** intense focal uptake, **e** diffuse and inhomogeneous uptake

Biodistribution and metabolism (Fig. 46)

Synthetic somatostatin peptides show long biological half-life and stronger and more specific affinity for somatostatin receptors available on the cellular surface of neuroendocrine tumours. DOTATATE, DOTATOC, and DOTANOC have different affinities for receptor subtypes (Kroiss et al. 2013; Bergeret et al. 2019).

Scan acquisition

• No special diet is required

• 2–3 MBq\Kg of ^68^Ga-DOTA-Peptide iv

• Uptake time 1 h

Clinical indications in oncology (Figs. 47, 48, 49, 50, 51, 52, 53, 54, 55, 56, 57, 58, 59, 60, 61, 62, 63, 64, and 65)

**Fig. 47:**
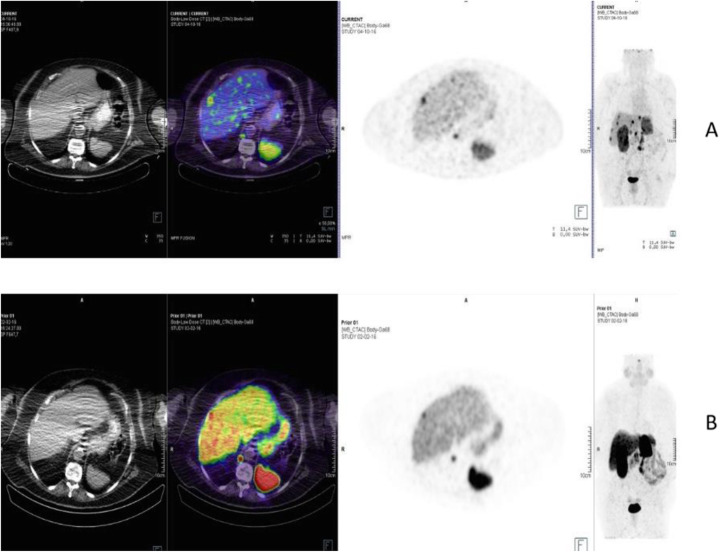
^68^Ga-DOTA peptide, follow-up of NET, comparison ^68^Ga-DOTANOC, ^68^Ga-DOTATATE. *Clinical history*: 68 y.o. woman with midgut NET and liver metastases treated with octreotide. *PET/CT findings*: ^68^Ga-DOTANOC (**a**) and ^68^Ga-DOTATATE (**b**) performed 8 months apart show similar uptake in the metastatic lesions, but due to the higher liver ^68^Ga-DOTATATE uptake, more metastases are identified in the ^68^Ga-DOTANOC study

**Fig. 48:**
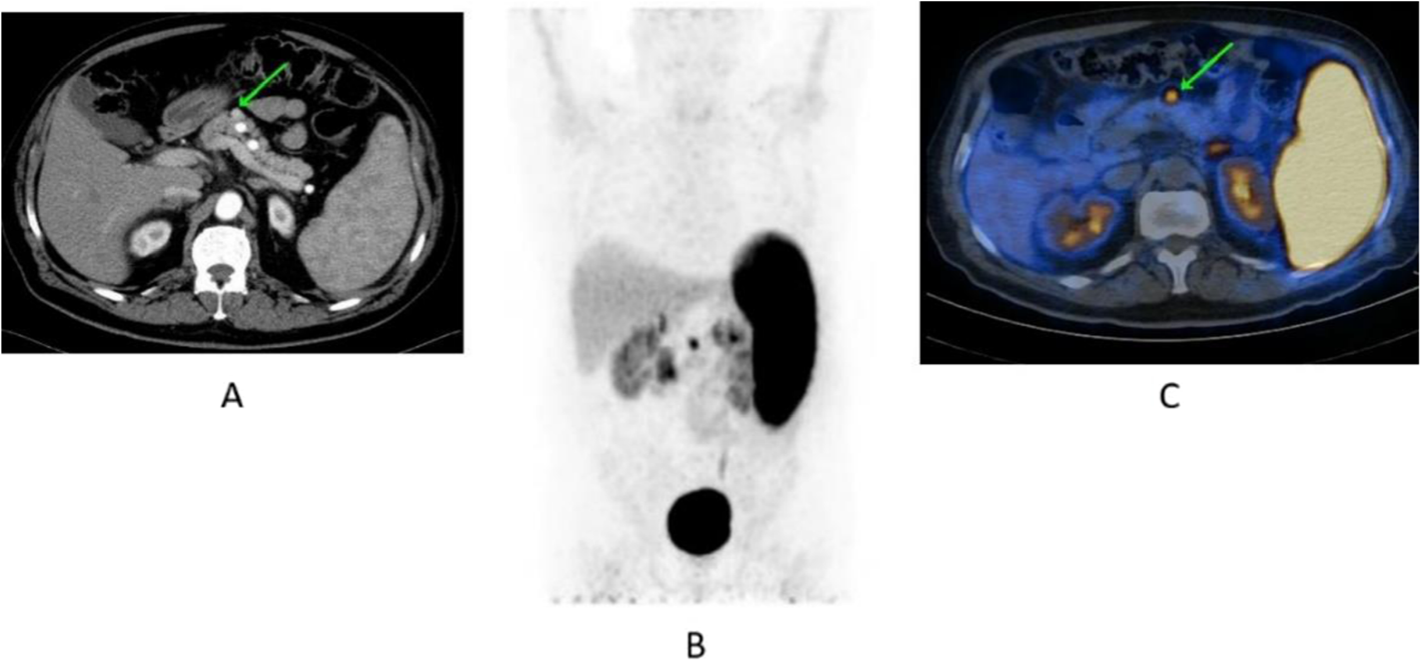
^68^Ga-DOTA peptide, suspected lesion of the pancreas, specificity. *Clinical history*: 50 y.o. woman; a hypervascular lesion in the pancreas was incidentally found on CT (**a**). *PET/CT findings*: focal area of high expression of somatostatin receptor analogues in the pancreas corresponding to the CT finding (**b** MIP, **c** fused). Surgery confirmed the presence of a well differentiated neuroendocrine tumour (Ki67 2%)

**Fig. 49:**
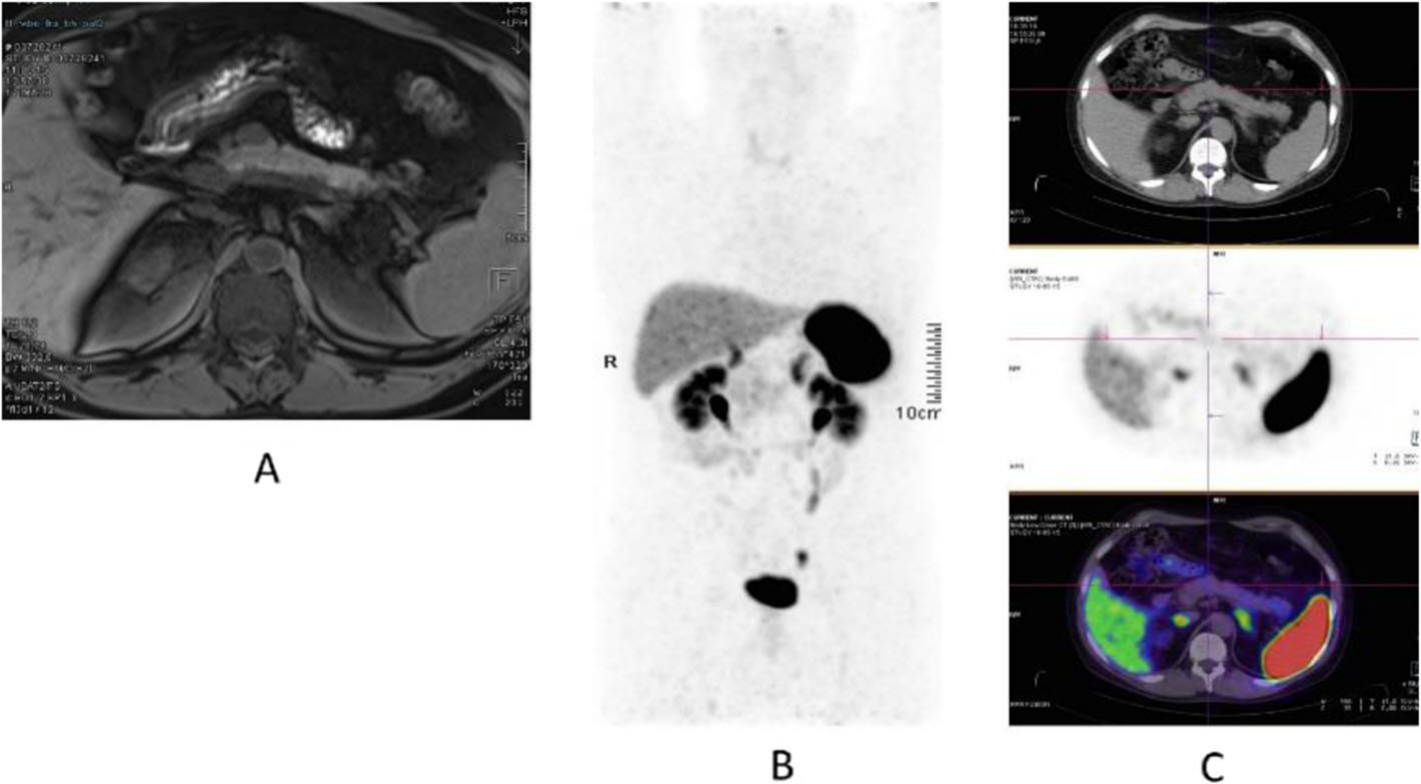
^68^Ga-DOTA peptide, suspected lesion of the pancreas, false negative. *Clinical history*: 48 y.o. man with lesion in the pancreatic body (**a** MRI). Clinical suspicion of insulinoma (hypoglycaemias). *PET/CT findings*: no uptake in the lesion (**b** MIP, **c** CT, PET, and fused images). The patient underwent surgery, which confirmed the presence of an insulinoma (frequently false negative with ^68^Ga-DOTA peptide)

**Fig. 50:**
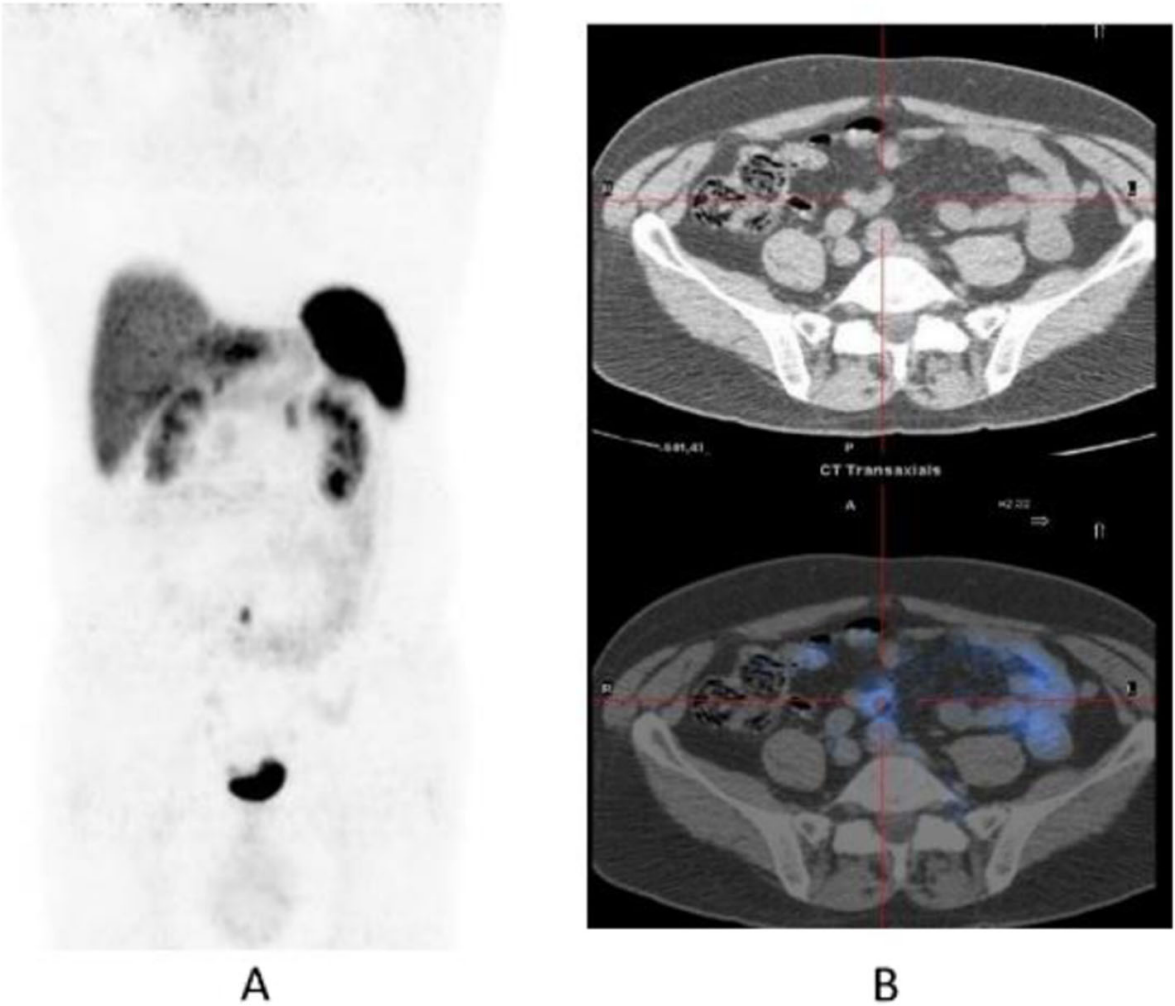
68Ga-DOTA peptide, suspected lesion of the liver, specificity. *Clinical history*: 52 y.o. male with an incidental detection of a single liver lesion in the left lobe. The lesion is suspect to be a metastasis on ultrasound and CT from an unknown primary site. *PET/CT findings*: increased focal uptake in a loop of the ileum, suspect to be a primary tumour. Increased uptake in the left liver lobe consistent with a secondary lesion (**a** MIP, **b** CT and fused images). After surgery, the lesions were diagnosed to be a well-differentiated NET grade 1 (Ki67 2%)

**Fig. 51:**
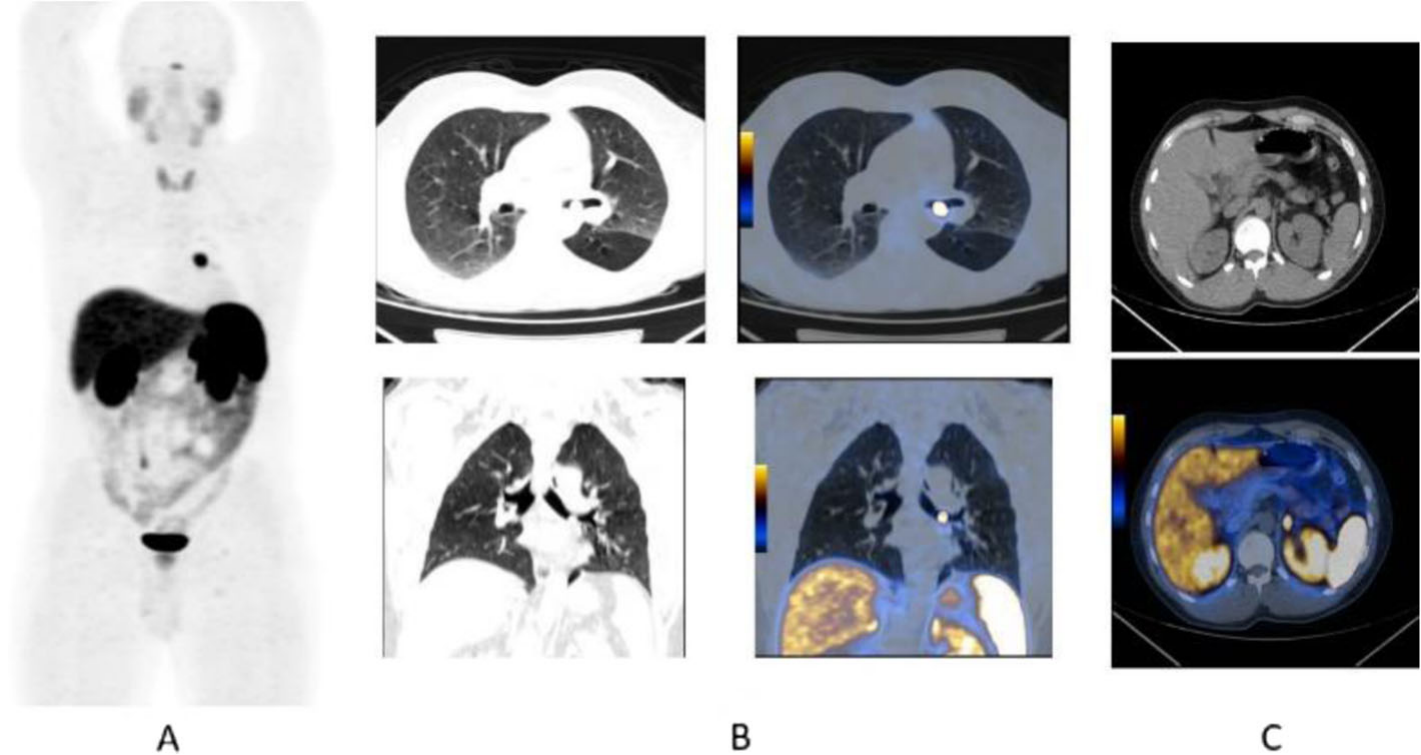
^68^Ga-DOTA peptide, staging of endobronchial carcinoid tumour, specificity. *Clinical history*: 33 y.o. man. Persistent cough and wheezing not responsive to bronchodilators. CT showed endobronchial node in left main bronchus. Bronchoscopy biopsy: NET. *PET/CT findings*: uptake in node protruding into the left main bronchus (**a** MIP, **b** CT and fused images). Focal uptake in the left adrenal gland (**c**). MRI confirmed the presence of an adrenal adenoma

**Fig. 52:**
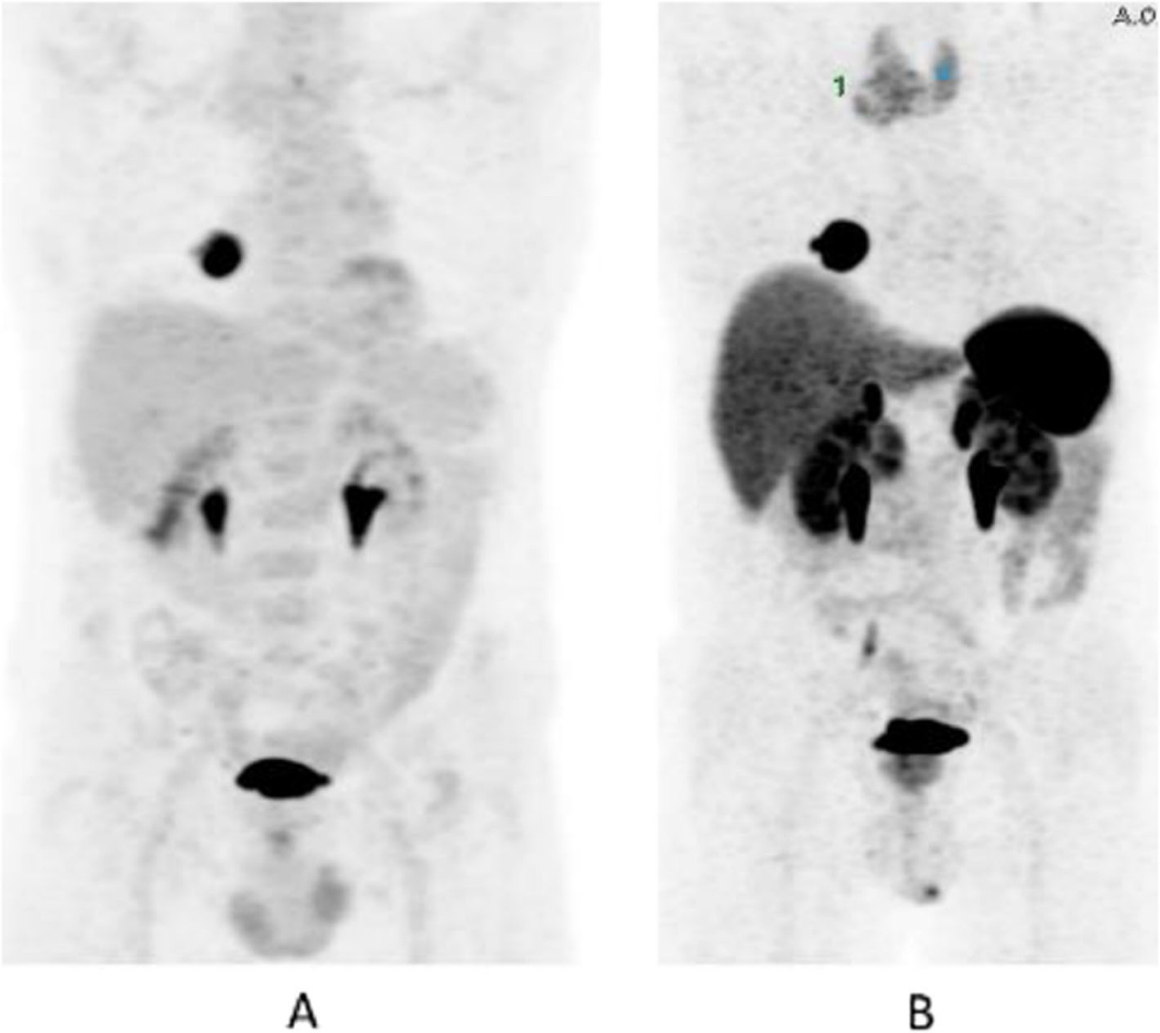
^68^Ga-DOTA peptide, staging of NET lung primary, comparison with FDG. *Clinical history*: 65 y.o. man. Staging of a lung mass with FDG PET and biopsy indicated a moderately differentiated NET (grade 2, Ki67 8%). Consequently, the patient underwent a second PET using ^68^Ga-DOTANOC to stage the disease more accurately. *PET/CT findings*: increased focal uptake in FDG PET in the right lung, without any other findings (**a** MIP). ^68^Ga-DOTANOC showed intense uptake in the lung and in the thyroid due to a known De Quervain thyroiditis (**b** MIP)

**Fig. 53:**
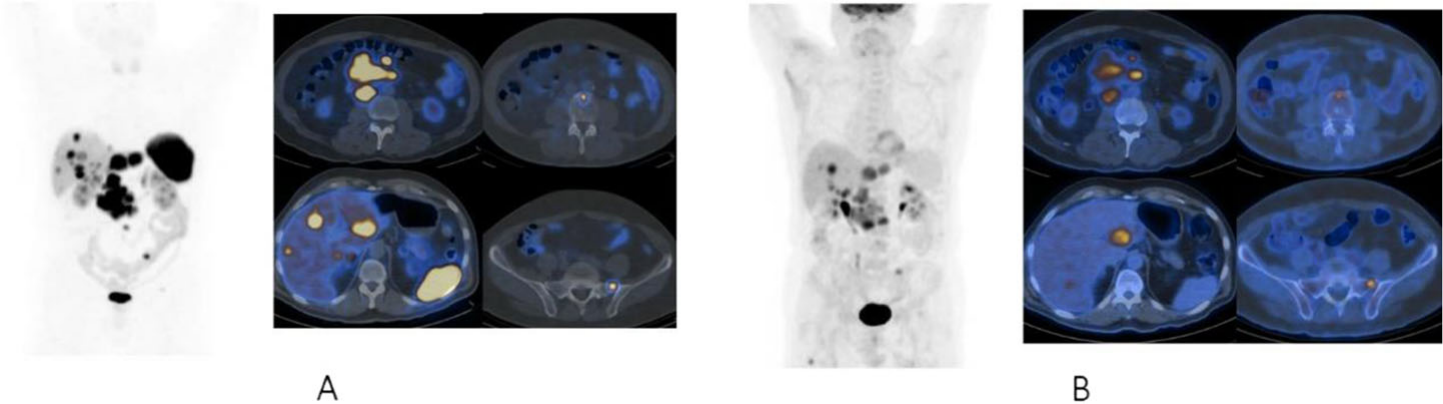
^68^Ga-DOTA peptide, staging NET of the pancreas, comparison with FDG. *Clinical history*: 68 y.o. man with moderately differentiated multi-metastatic NET of pancreas (Ki67 8%). *PET/CT findings*: ^68^Ga-DOTANOC PET/CT shows intense pathologic uptake of somatostatin receptor analogue by the known pancreatic tumour, as well as in lymph nodes, multiple liver lesions, and previously unknown bone lesions (**a**). FDG-PET/CT confirms pathologic uptake of the tracer in pancreas and lymph nodes and in some of the known liver and bone lesions (**b**)

**Fig. 54:**
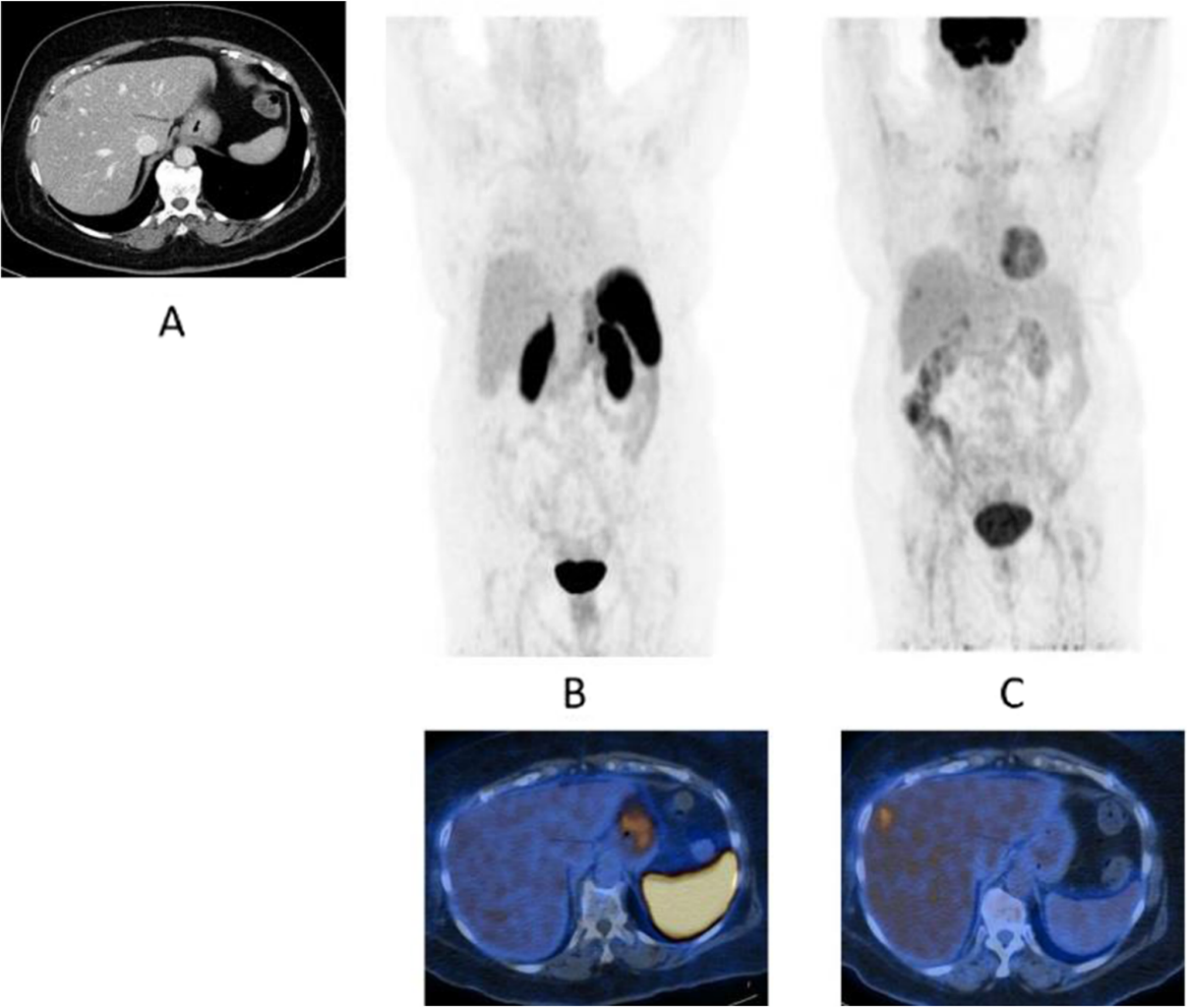
68Ga-DOTA peptide, NET of the pancreas, suspicion of relapse, comparison with FDG. *Clinical history*: 63 y.o. man with known well differentiated pancreatic NET (Ki67:2%); routine follow-up CT detected a suspicious lesion in the liver (**a**); PET/CT was requested to restage the patient. *PET/CT findings*: ^68^Ga-DOTANOC PET/CT shows only physiological uptake of somatostatin receptor analogue (**b** MIP and fused PET/CT). FDG-PET/CT shows a mild pathologic uptake of the tracer in the liver and confirms liver involvement (**c** MIP and fused PET/CT)

**Fig. 55:**
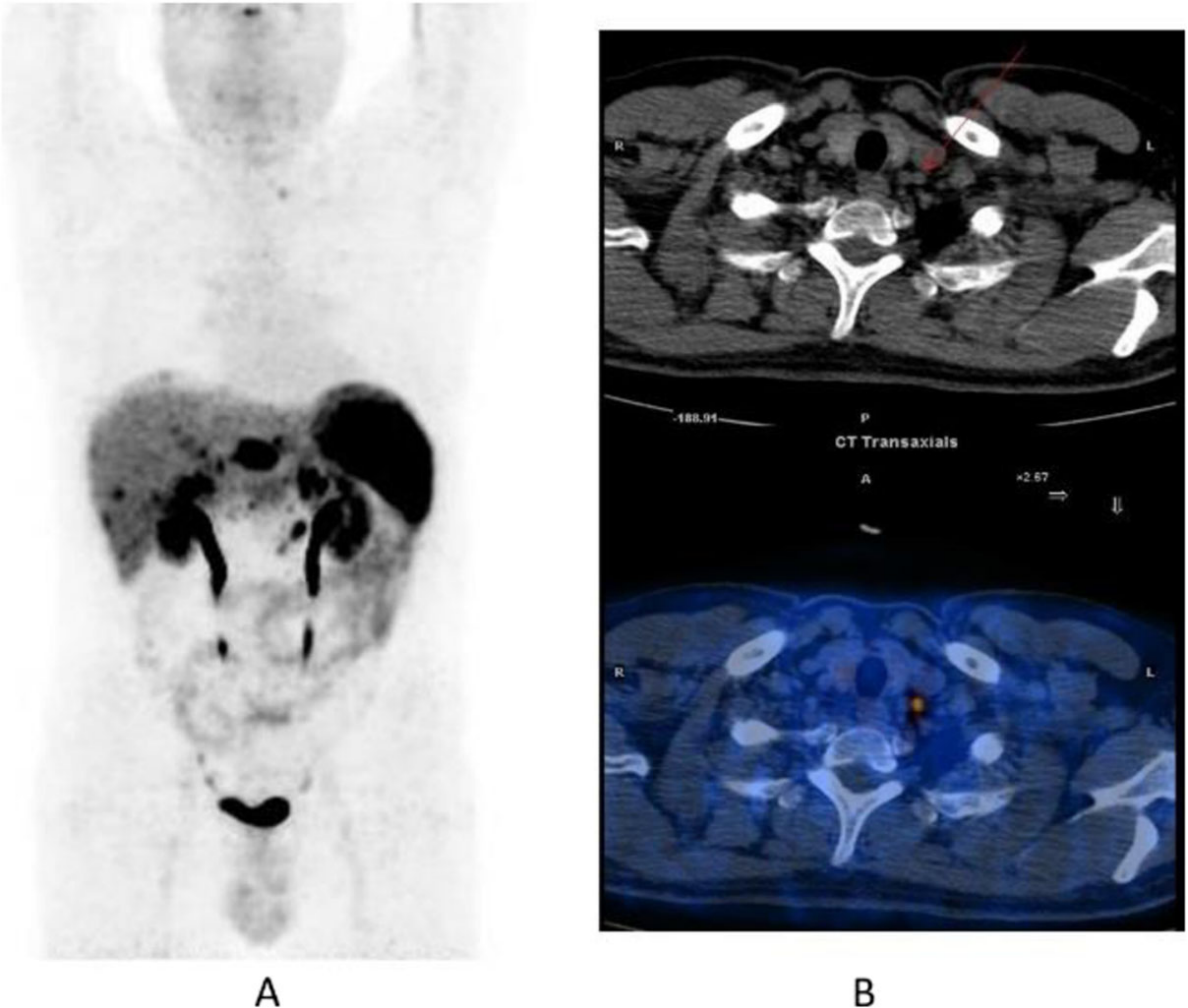
^68^Ga-DOTA peptide, staging NET of the pancreas, sensitivity. *Clinical history*: 60 y.o. man. Incidental finding of a NET of the pancreas grade 2 (Ki67 5%). *PET/CT findings*: multiple areas of increased tracer uptake in the pancreas, liver, and abdominal lymph nodes (**a** MIP). Note the uptake in a left supraclavicular lymph node 5 mm in size (**b** CT and fused images)

**Fig. 56:**
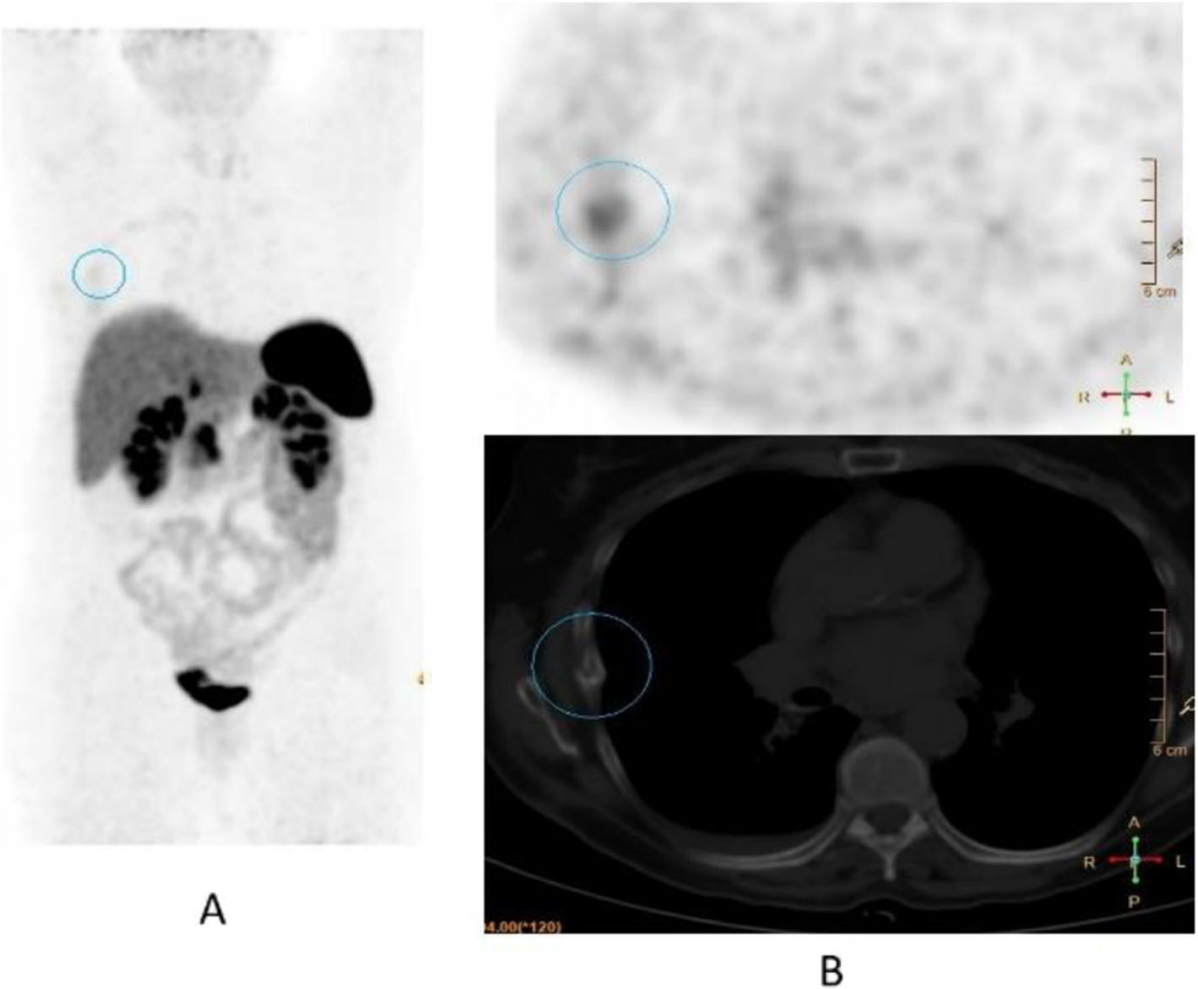
^68^Ga-DOTA peptide, restaging after surgery for lung carcinoid, specificity. *Clinical history*: 60 y.o. man; restaging after surgery for lung carcinoid. *PET/CT findings*: focal increased ^68^Ga-DOTATOC uptake in a benign rib fracture (**a** MIP, **b** PET and fused PET/CT, blue marker)

**Fig. 57:**
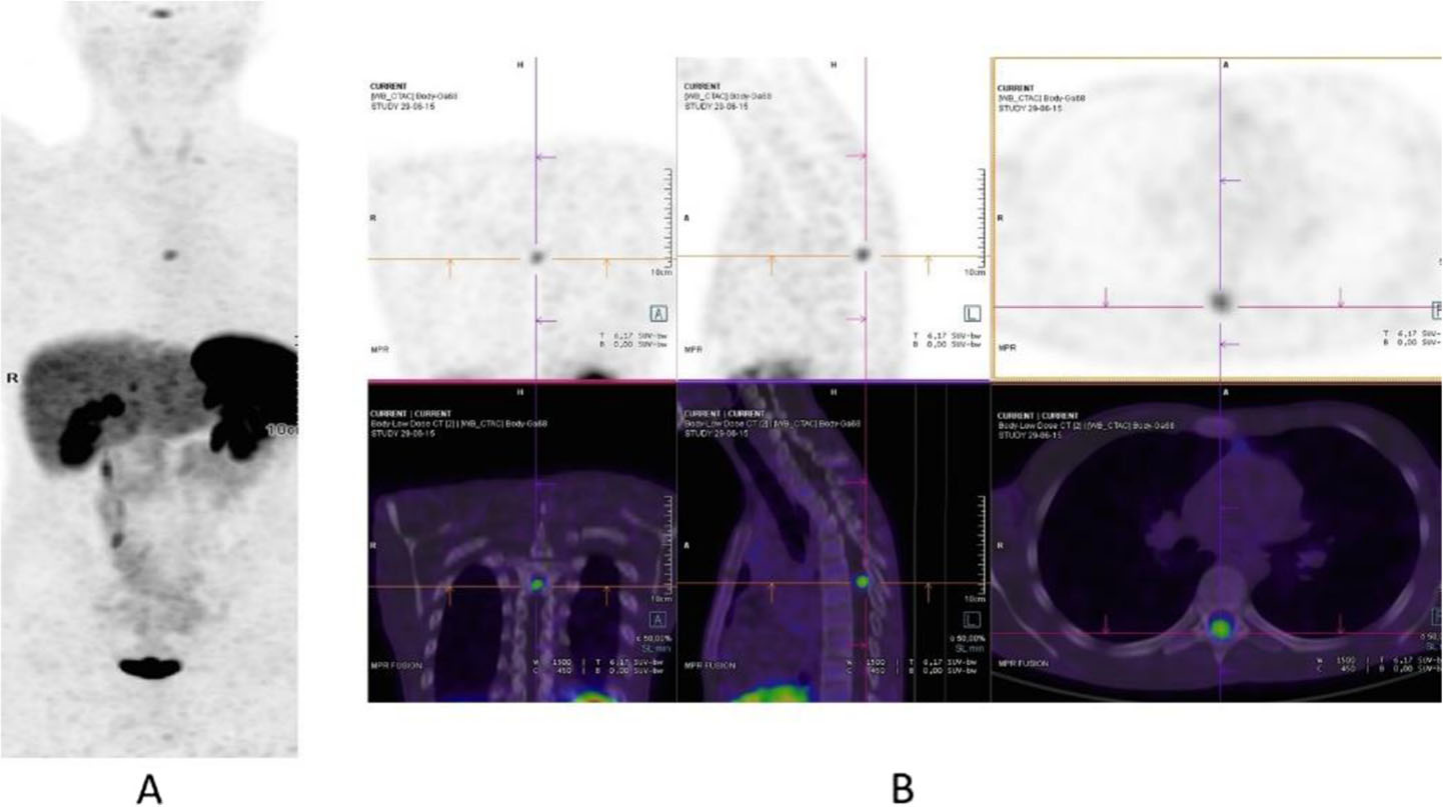
^68^Ga-DOTA peptide, suspicion of NET, specificity. *Clinical history*: 17 y.o. boy with known Von Hippel-Lindau disease. Increased level of pancreatic polypeptide. *PET/CT findings*: ^68^Ga-DOTANOC PET shows an intramedullary focus of increased uptake at the level of T8 (**a** MIP, **b** PET and fused PET/CT), corresponding to a hemangioblastoma

**Fig. 58:**
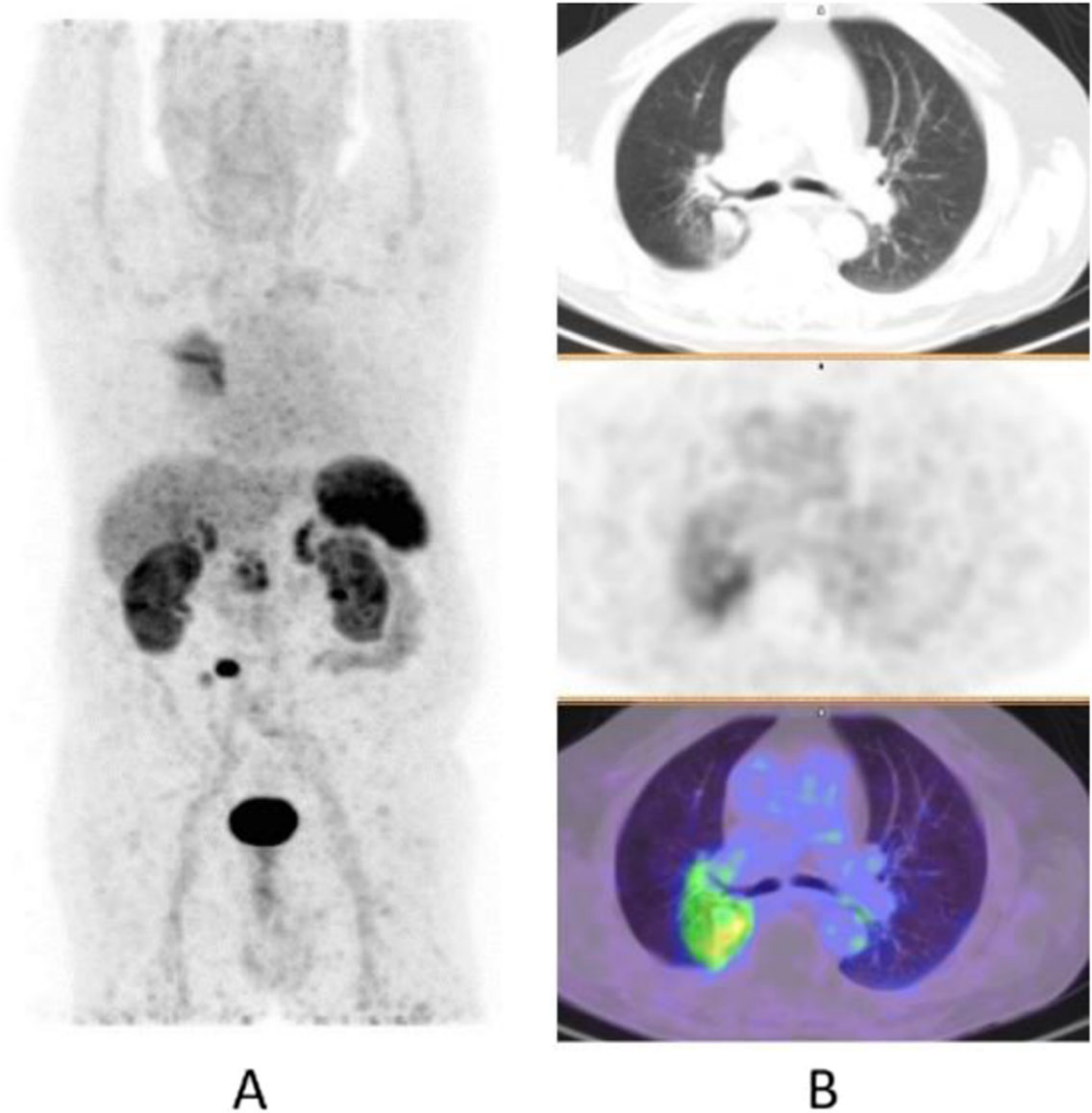
^68^Ga-DOTA peptide, follow-up of midgut NET, specificity. *Clinical history*: 69 y.o. woman with NET treated by surgery the year before. Right upper lobe lung adenocarcinoma treated by chemo- and radiation therapy 4 years prior. *PET/CT findings*: in addition to multifocal recurrent disease (**a** MIP), ^68^Ga-DOTANOC PET shows moderately increased uptake in the previously irradiated lung parenchyma (**b**)

**Fig. 59:**
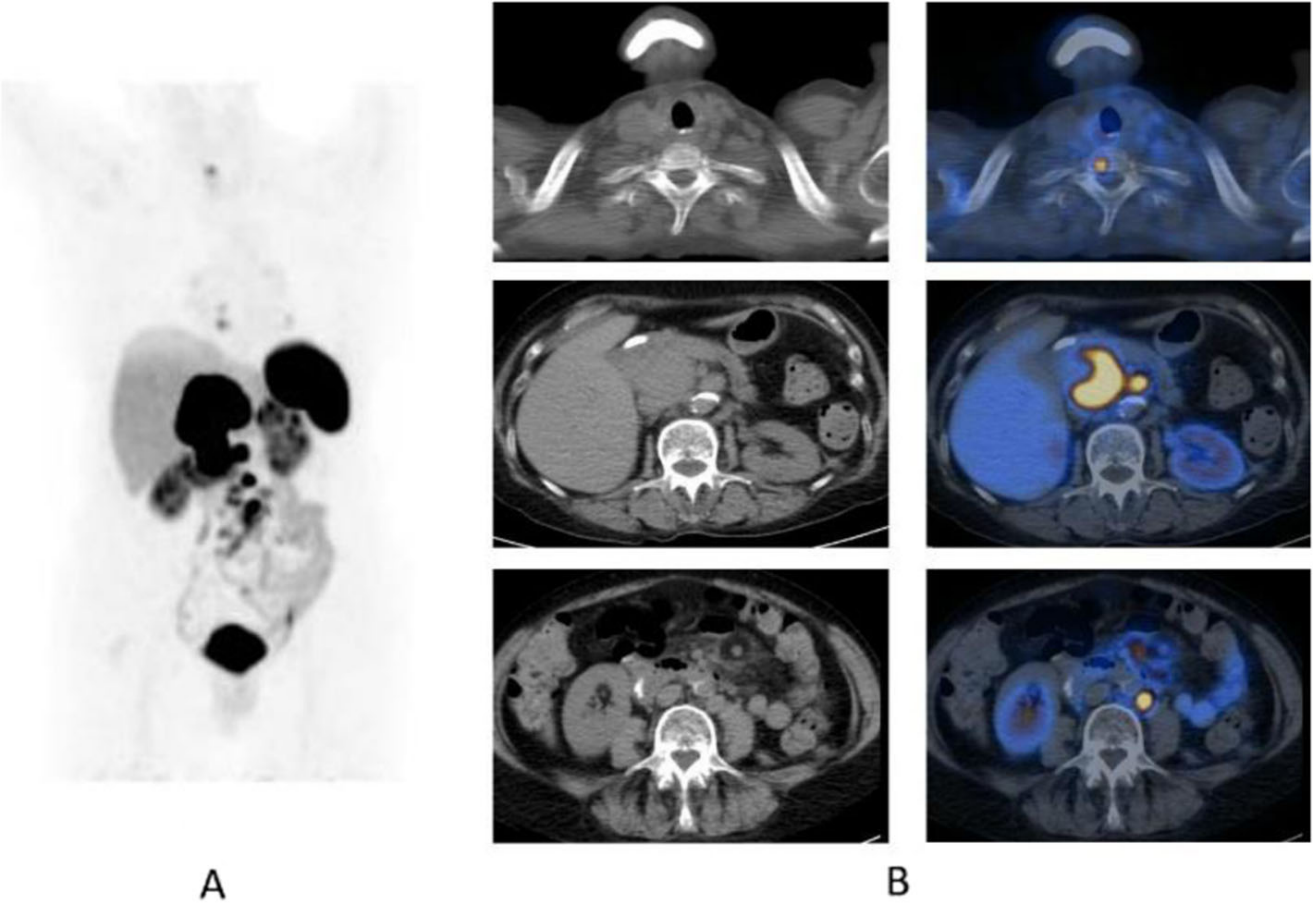
^68^Ga-DOTA peptide, staging NET of the pancreas, sensitivity. *Clinical history*: 54 y.o. man with known pancreatic NET, candidate for surgery. *PET/CT findings*: very high uptake in the known pancreatic lesion and as well as in previously unknown lymph nodes and bone lesions (**a** MIP, **b** CT and fused imaging)

**Fig. 60:**
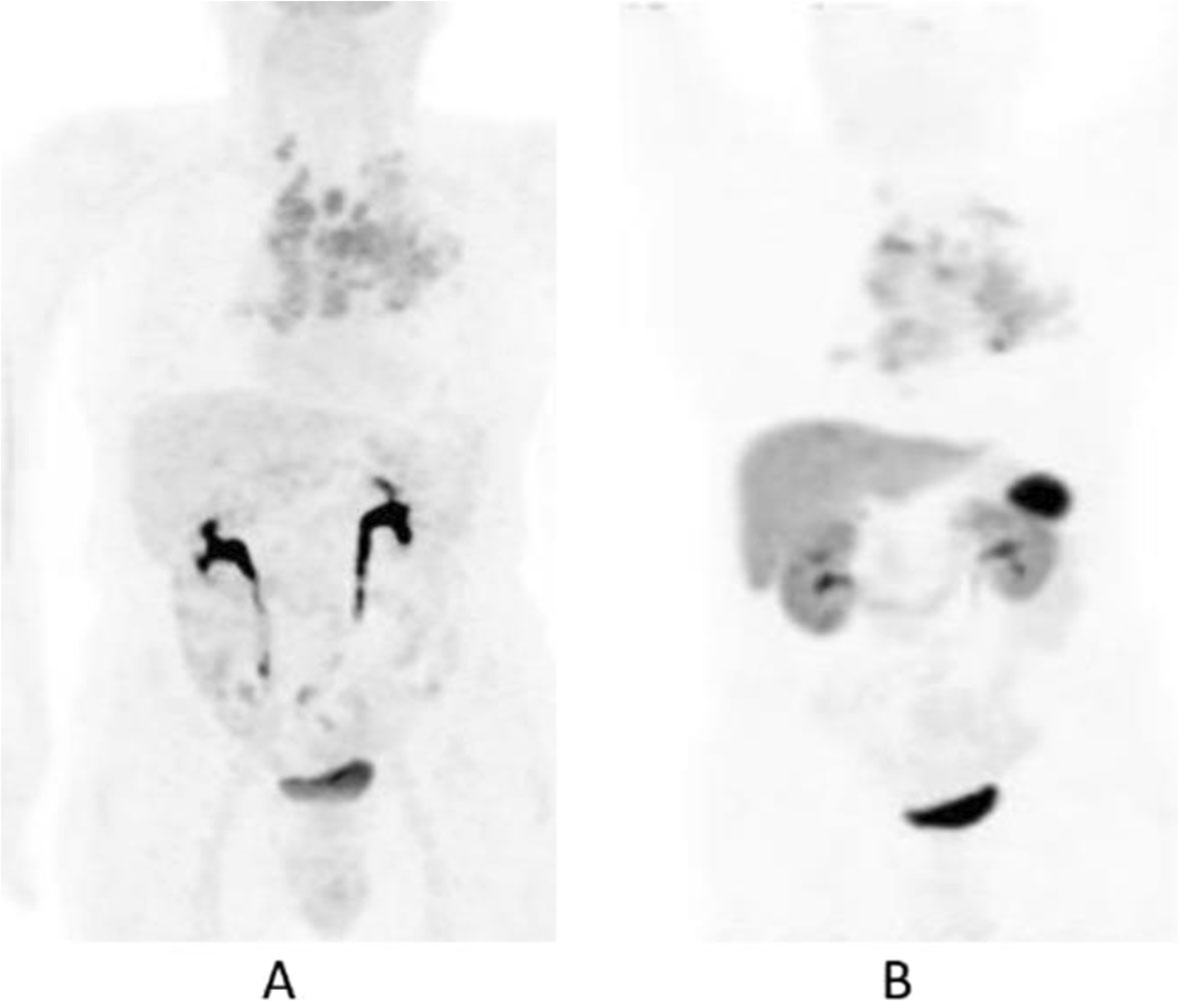
^68^Ga-DOTA peptide, suspected NET of the pancreas, false positive (inflammation), compared with FDG. *Clinical history*: 65 y.o. man with suspected NET of the pancreas. *PET/CT findings*: FDG: there is no significant uptake in the pancreas. Intense symmetric uptake in mediastinal lymph nodes (**a** MIP). ^68^Ga-DOTANOC: there is increased symmetrical uptake in mediastinal lymph nodes but no significant uptake in the pancreas (**b** MIP)

**Fig. 61:**
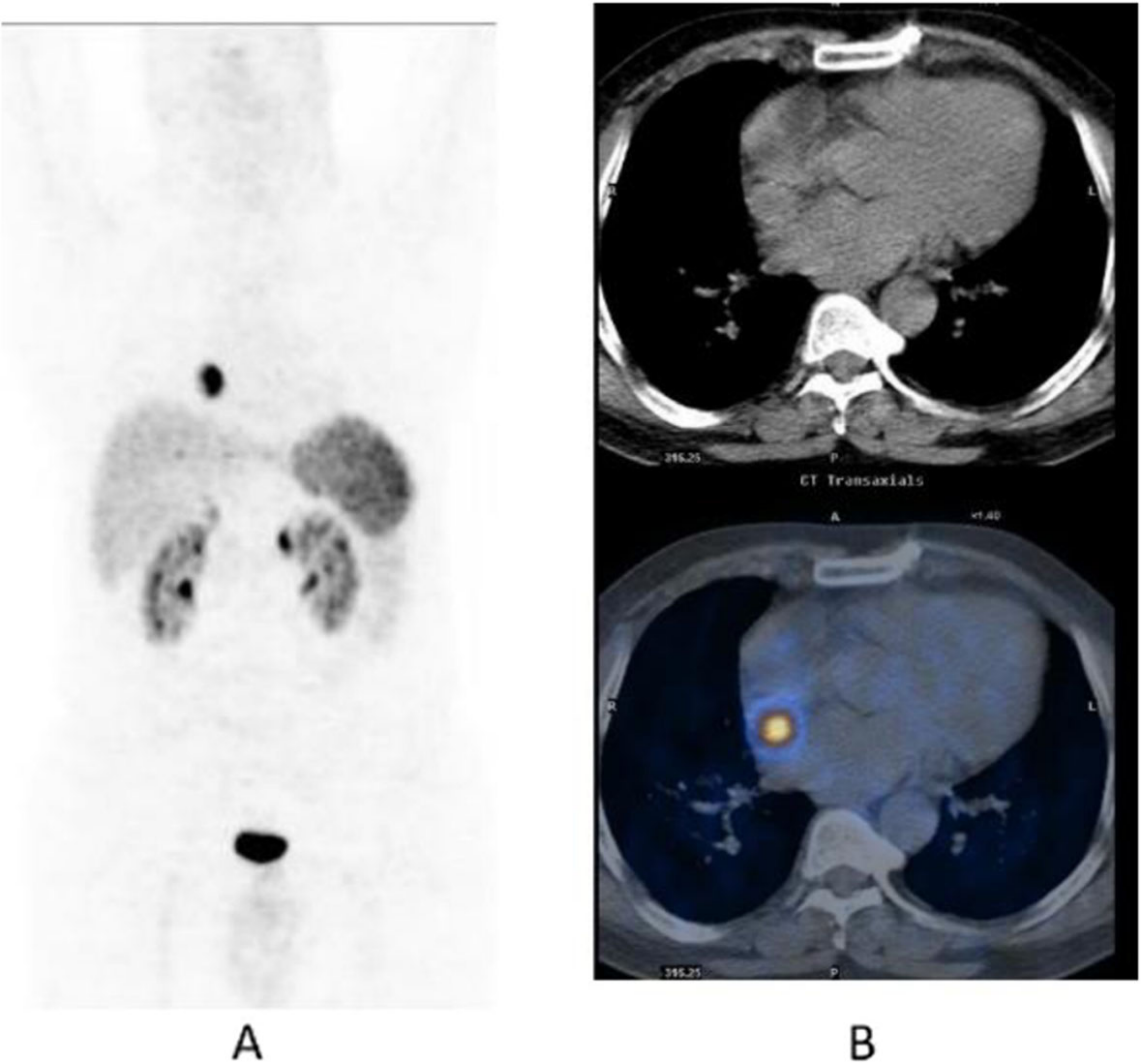
^68^Ga-DOTA peptide, suspected recurrence of paraganglioma. *Clinical history*: 39 y.o. man with a previous history of paragangliomas. During follow-up CT suspected a relapse in the thorax. *PET/CT findings*: intense focal uptake in a para-caval round shaped lesion (**a** MIP, **b** CT and fused PET/CT) consistent with a paraganglioma

**Fig. 62:**
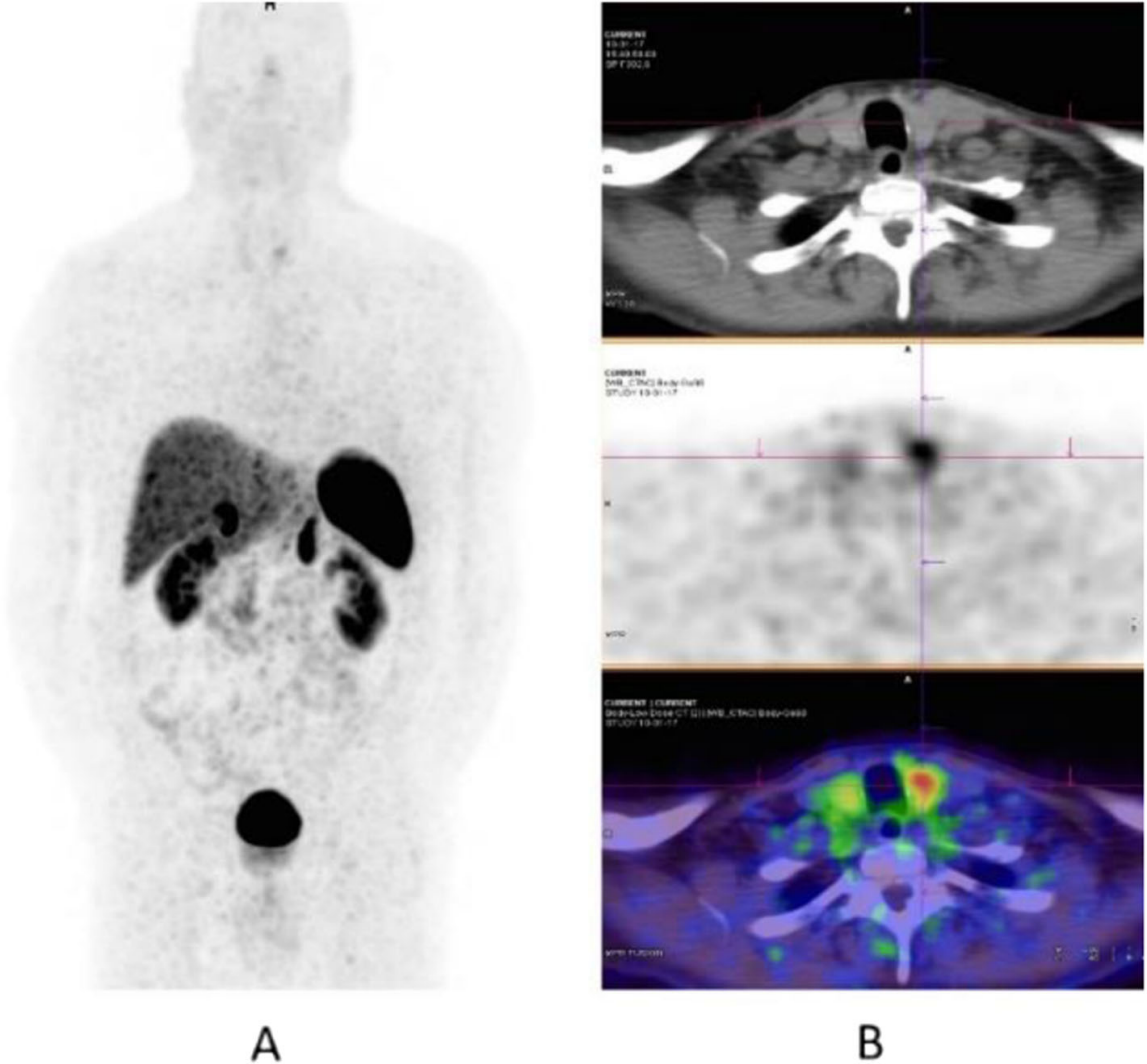
68Ga-DOTA peptide, staging medullary thyroid cancer (MTC). *Clinical history*: 70 y.o. man with newly diagnosed MTC. Suspicion of distant metastases on CT. *PET/CT findings*: moderately increased uptake in the known tumour (**a** MIP, **b** CT, PET, and fused imaging). No other suspicious lesions are seen

**Fig. 63:**
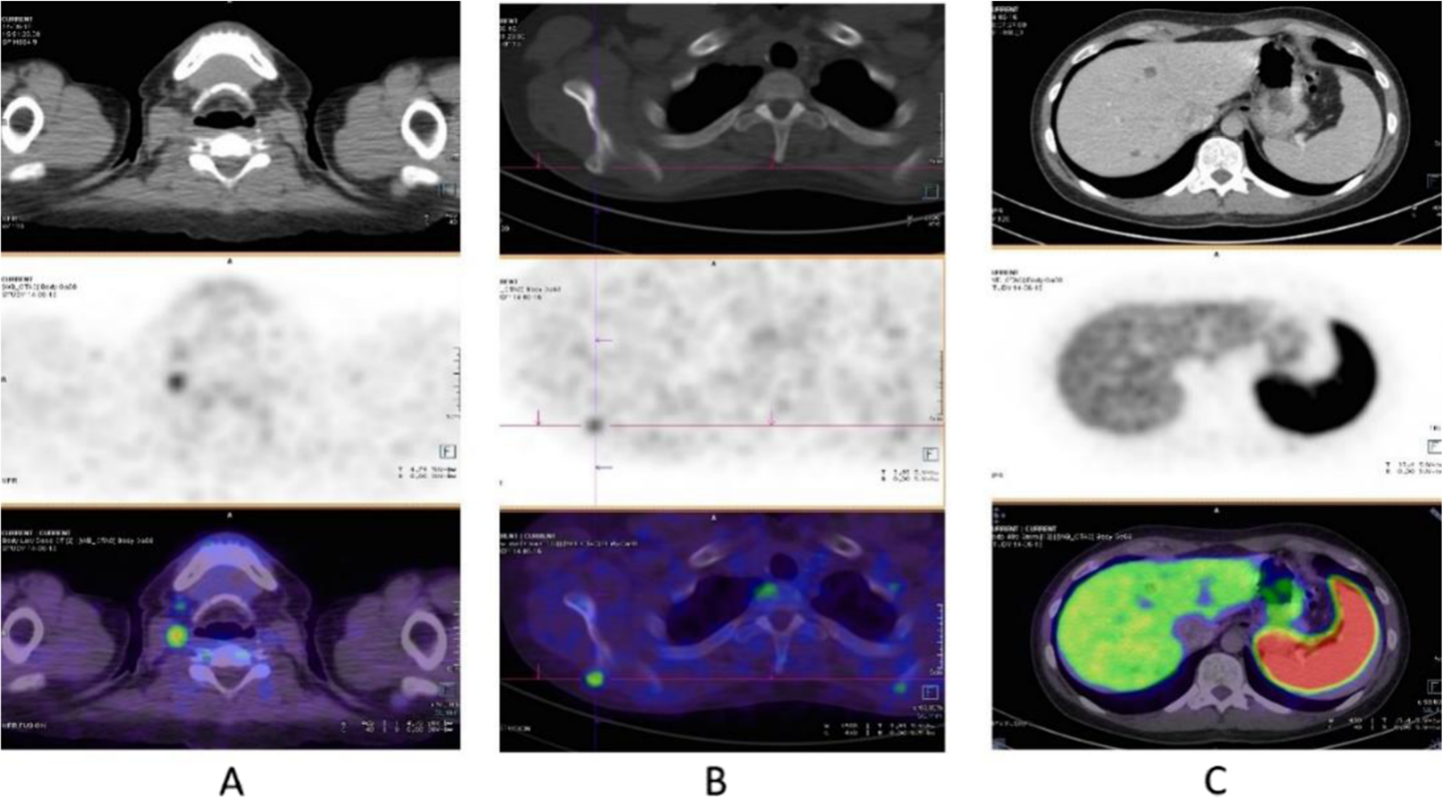
^68^Ga-DOTA peptide, recurrent medullary thyroid cancer (MTC). *Clinical history*: 56 y.o. man with previous history of MTC, treated by surgery. Progressive increase in calcitonin (TCT = 19200). *PET/CT findings*: increased uptake in cervical LN (**a**) and bone lesion (**b** right scapula). Additional hepatic metastases are seen on the diagnostic CT, but due to the high background they are not evident on PET images (**c**)

**Fig. 64:**
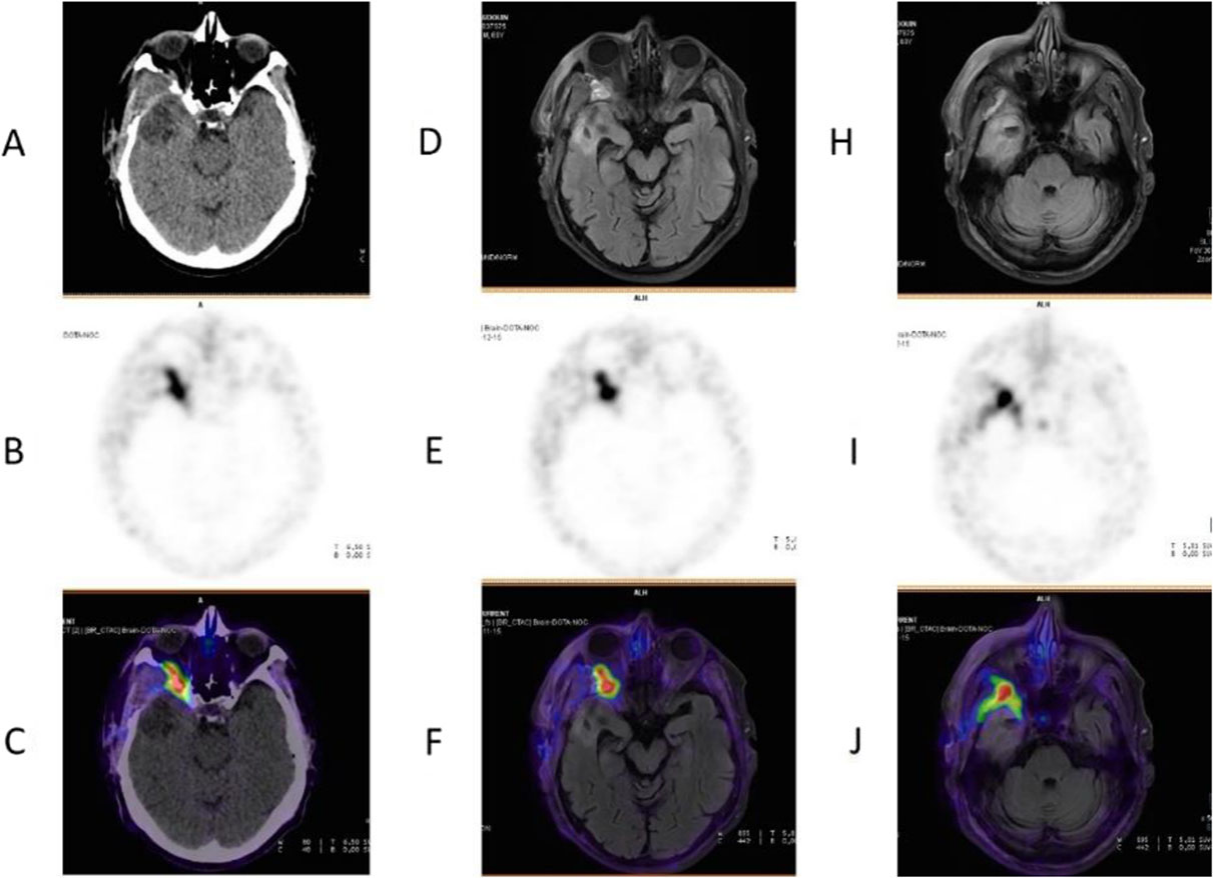
^68^Ga-DOTA peptide, meningioma. *Clinical history*: 70 y.o. man with meningioma of the skull basis. *PET/CT findings*: ^68^Ga-DOTATATE shows highly increased uptake in the right sphenoidal bone area with an extension to the orbital cavity, corresponding to the meningioma (**a** CT; **d**, **h** MRI; **d**, **e**, **i** PET; **c** fused PET/CT; **f**, **j** fused PET and MRI)

**Fig. 65:**
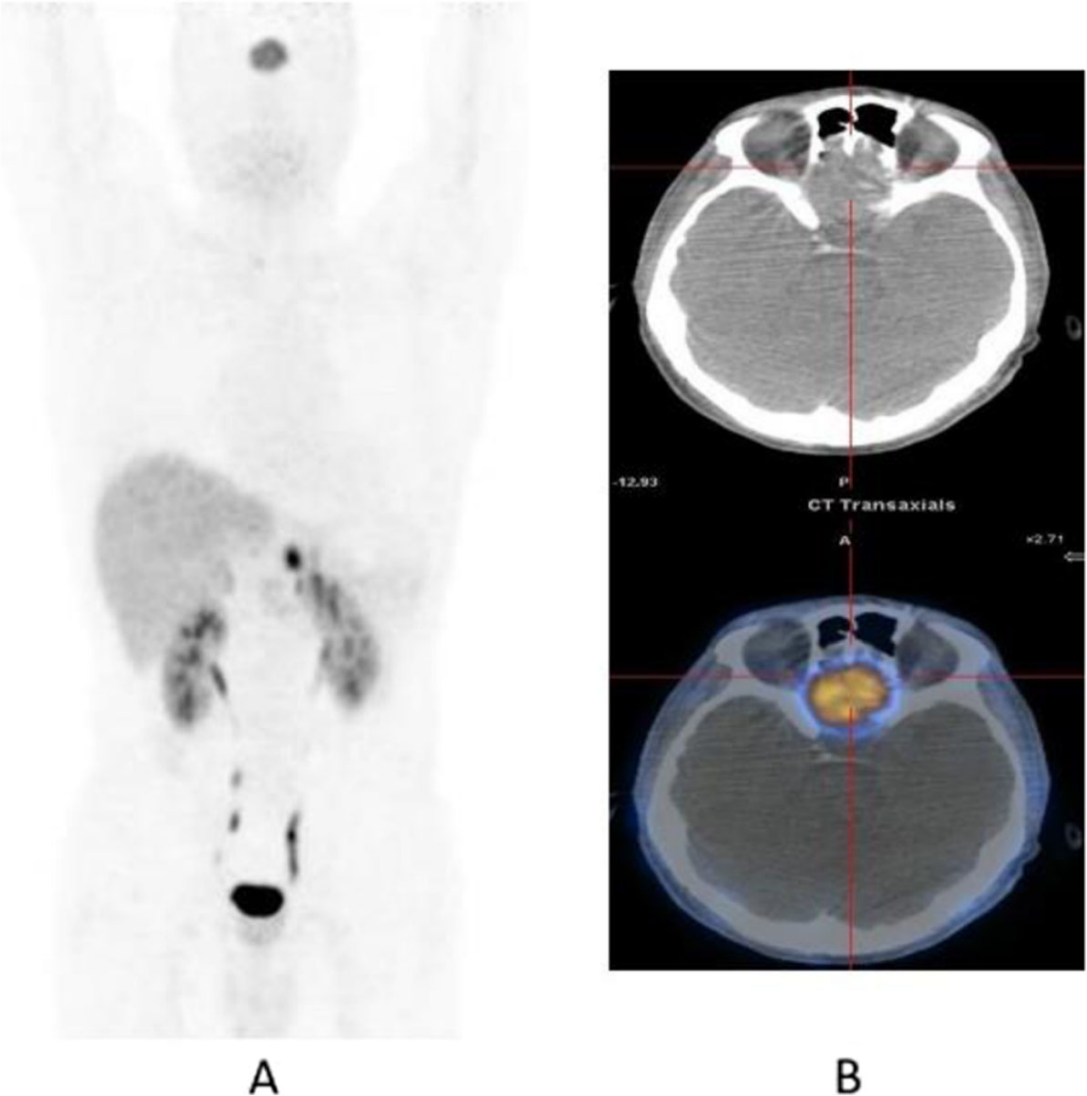
^68^Ga-DOTA peptide, meningioma. *Clinical history*: 57 y.o. woman with history of a NET of the tail of the pancreas (grade 1) treated with surgery. Suspect relapse in a peripancreatic lymph node. *PET/CT findings*: increased uptake in a peripancreatic lymph node consistent with relapse (**a** MIP). Intense uptake in a large lesion in the base of the cranium consistent with a meningioma (**b** CT and fused imaging)

In the management of NETs ^68^Ga-DOTA-conjugated peptide, PET/CT is used to localise primary tumours and detect sites of metastatic disease (staging); follow-up patients with known disease to detect residual, recurrent or progressive disease (restaging); determine somatostatin status; monitor response to therapy; and select patients with metastatic disease for peptide receptor radionuclide therapy (Skoura et al. 2016; Sundin 2018; Singh et al. 2018; Waseem et al. 2019).

### FMISO

Names: 1-(2-Nitro-imidazolyl)-3-[^18^F] fluoro-2-propanol; ^18^F-FMISO

**Fig. 66:**
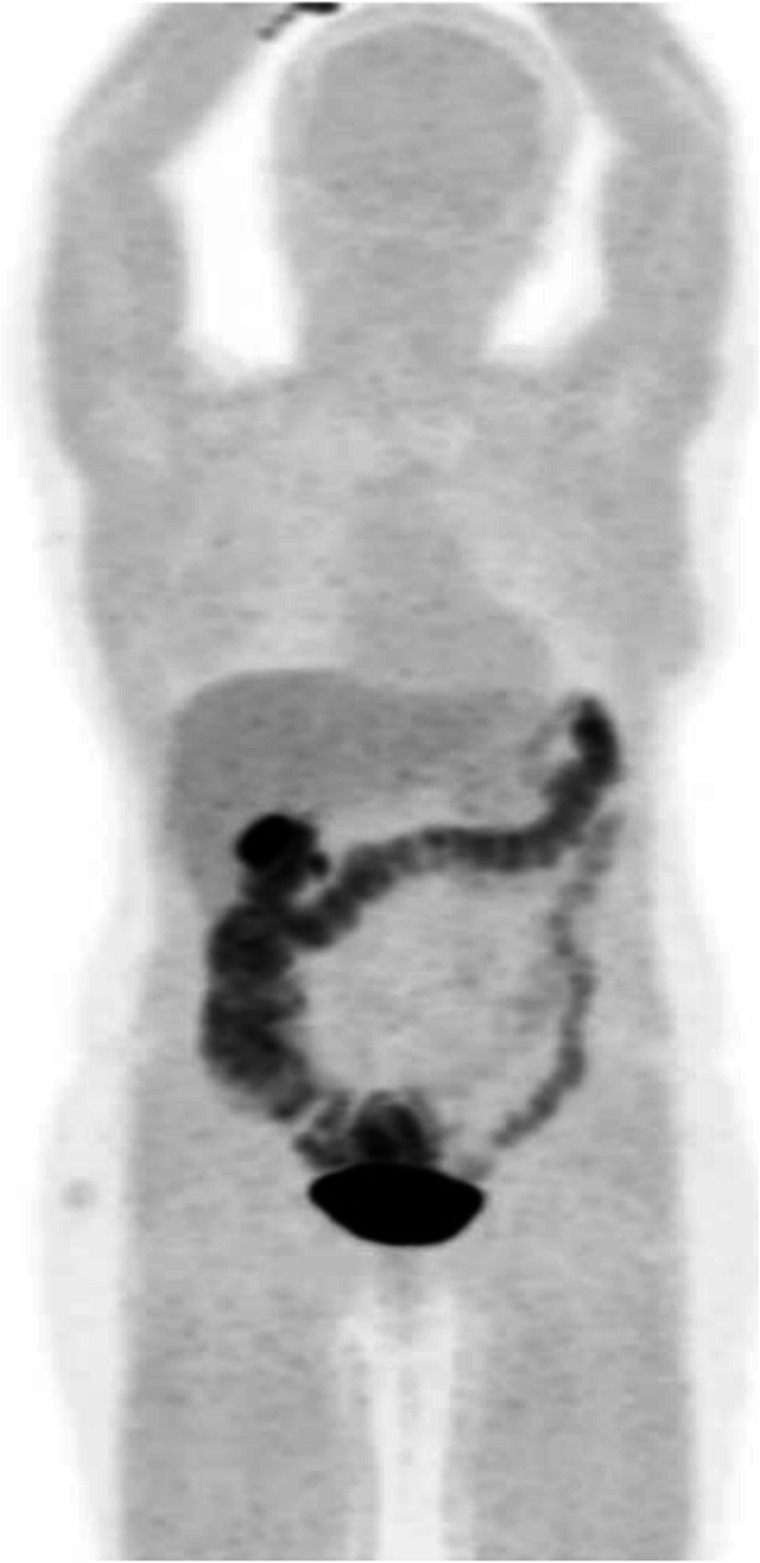
Physiological bio-distribution of 18F-FMISO (similar to the physiological biodistribution of ^18^F-FAZA)

Biodistribution and metabolism (Fig. 66)

Nitro-group are postulated to undergo reduction in hypoxic condition (pO2 ≤ 2–3 mmHg), forming highly reactive oxygen radicals that subsequently bind covalently to macromolecules inside the cells (Visser et al. 2014; Orlefors et al. 2005).

^18^F-FMISO is relatively hydrophilic and diffuses across cell membranes, showing a passive distribution in normal tissues, resulting in slow clearance kinetics and a high lipophilicity, resulting in substantially high background.

Scan acquisition

• Fasting for at least 2 h

• 6 MBq\Kg of ^18^F-FMISO iv

• Uptake time 3–4 h

**Fig. 67:**
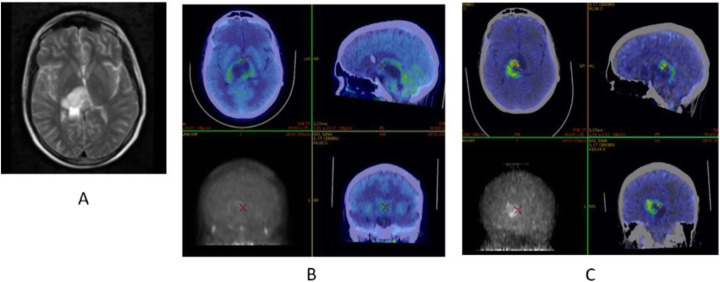
^18^F-FMISO, characterisation of a brain lesion and comparison to ^11^C-methionine. *Clinical history*: 15 y.o. boy. High-grade glioblastoma, sub-totally removed and treated with radiotherapy. MRI: persistence of expansive lesion in thalamic area (**a**). *PET/CT findings*: ^11^C-methionine shows peripheral uptake in the right thalamus tumour, probably related to residual viable tumour or relapse despite its low intensity (**b**). ^18^F-FMISO PET/CT showed hypoxic area in previously methionine uptake described lesion (**c**), suggesting a relapse

Clinical indications in oncology (Fig. 67)

PET-CT with ^18^F-FMISO is a non-invasive method for detecting and characterising hypoxia in several tumours. Ischemia in tumours is associated with a poor prognosis, increased invasion rate, metastasis, and resistance to chemo- and radiation therapy (Institute NC 2013; Nehmeh et al. 2008; Gagel et al. 2006; Hirata et al. 2012; Lin et al. 2008; Lopci et al. 2014; Reischl et al. 2007; Wack et al. 2015).

### FAZA

Names: 1-(5-[^18^F] Fluoro-5-deoxy-α-D-arabinofuranosyl)-2-Nitroimidazole; ^18^F-FAZA

Biodistribution and metabolism (Fig. 66)

F-18 FAZA is a 2-nitroimidazole compound (reduced in hypoxic cellular media) with a sugar addition moiety showing more water solubility and better pharmacokinetics compared to ^18^F-FMISO (Zips et al. 2012; Bollineni et al. 2013; Bollineni et al. 2014).

Scan acquisition

• No special diet is required

• 370 MBq of ^18^F-FAZA iv

• Uptake time 2 h

**Fig. 68:**
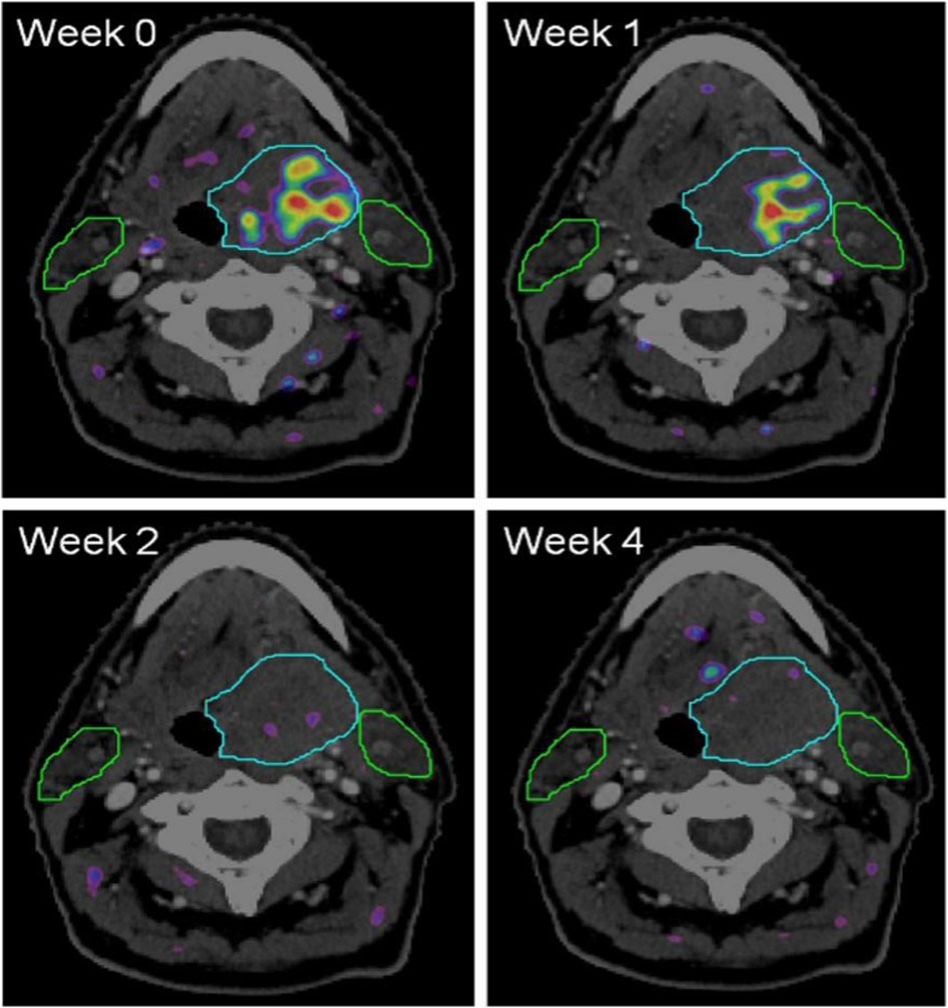
^18^F-FAZA, therapy evaluation of a brain lesion. *PET/CT findings*: four FAZA-PET-CT scans made at different weeks prior (week 0) and during (week 1, 2, and 4) the course of chemoradiation. The rainbow colours depict the amount of FAZA uptake. The light blue line depicts the extent of the primary tumour situated in the base of tongue. The green lines depict the extent of the parotid glands (left and right). Note that in week 2 and 4 no increased FAZA uptake is visible any more, demonstrating that the hypoxic area in week 0 and 1 disappeared

Clinical indications in oncology (Fig. 68)

The indications are similar to ^18^F-FMISO (Reischl et al. 2007; Wack et al. 2015; Zips et al. 2012).

### NAF

Names: [^18^F]-Sodium fluoride; ^18^F-NaF

**Fig. 69:**
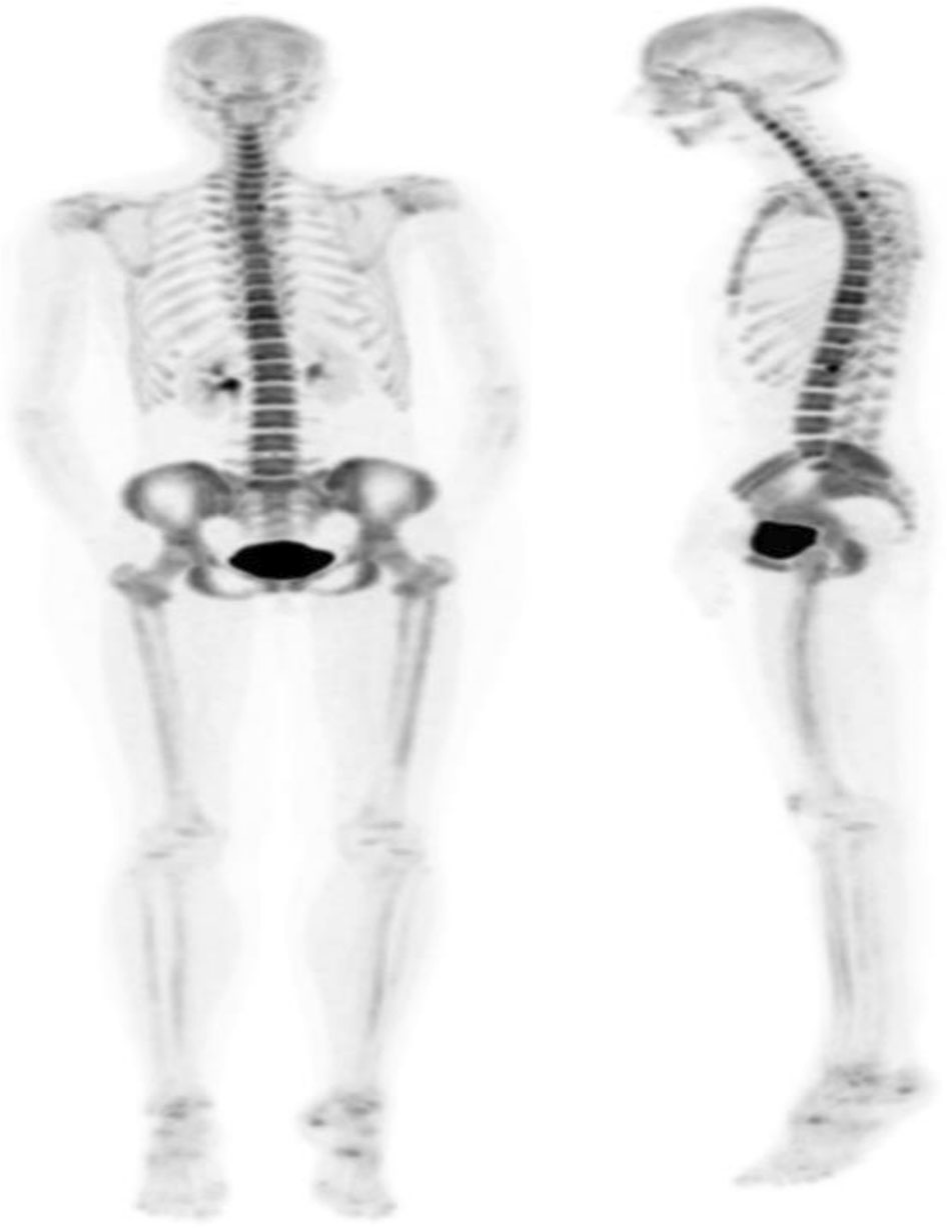
Physiological biodistribution of ^18^F-NaF

Biodistribution and metabolism (Fig. 69)

Fluoride ions are deposited in the bone matrix and reflect: bone remodelling and blood flow. The target organ is bone, but approximately 20% is excreted through the kidney in the urine in the first 1-2 h (Bruine de Bruin et al. 2015; Beheshti et al. 2015).

Scan acquisition

• No special diet is required but good hydration is important

• 50–200 MBq of ^18^F-NaF iv

• Uptake time 20–60 min

**Fig. 70:**
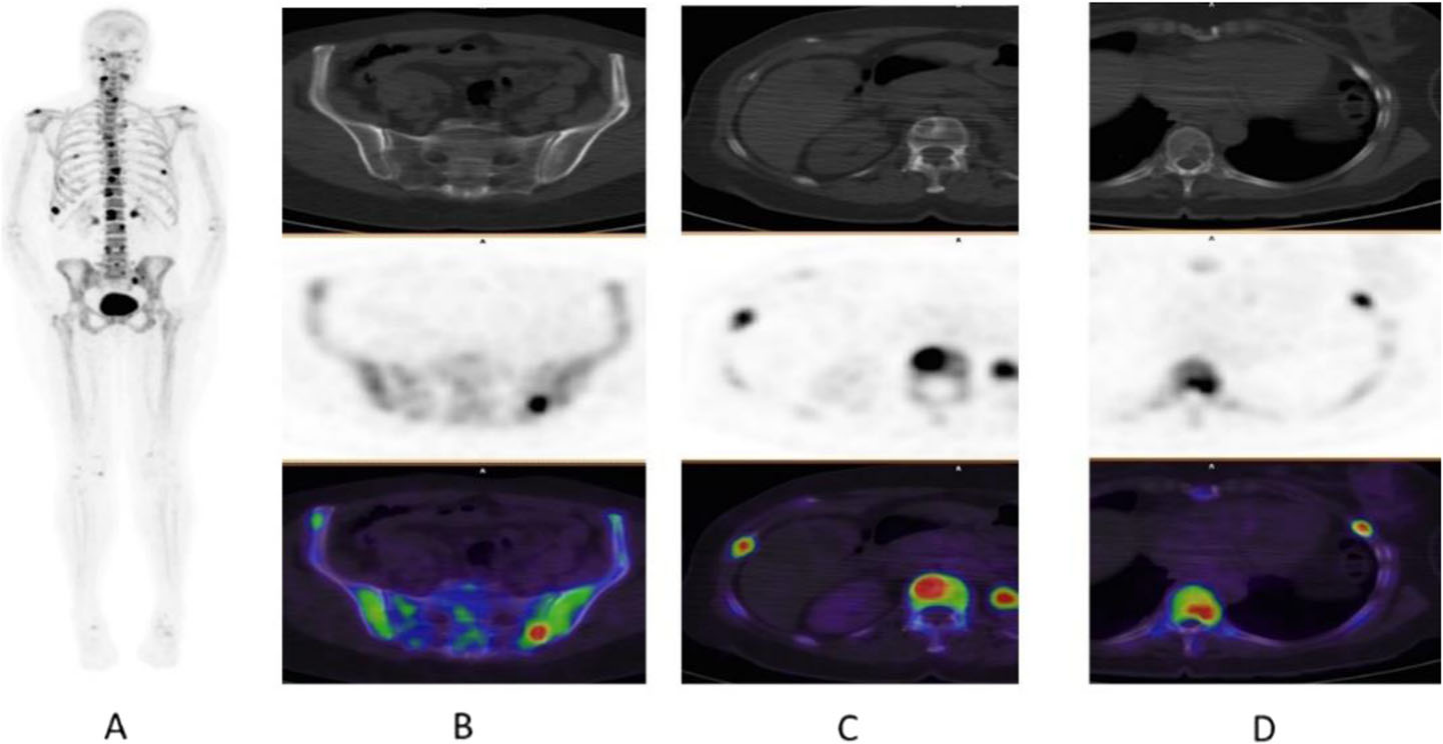
^18^F-NaF, breast cancer staging. *Clinical history*: 55 y.o. woman recently diagnosed with breast cancer (ductal adenocarcinoma, ER+, PR, and HER2 neg), asymptomatic. *PET/CT findings*: multiple foci of increased tracer uptake consistent with skeletal metastatic spread (**a** MIP). Note the heterogeneity of the CT appearance in this patient: some lesions are osteolytic (**b**), others are sclerotic (**c**) or do not show any CT anomaly (**d**)

**Fig. 71:**
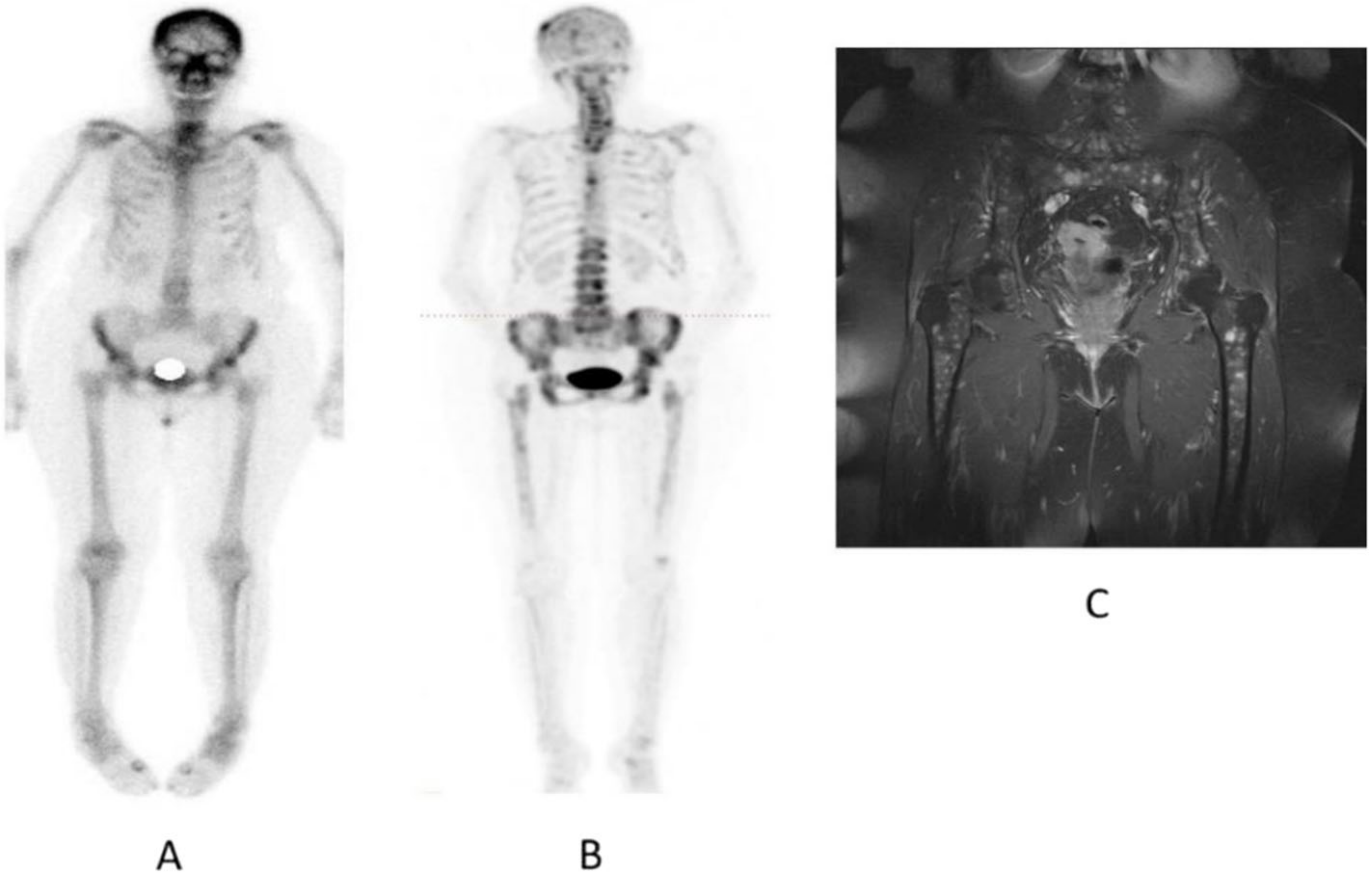
18F-NaF, breast cancer restaging. *Clinical history*: 72 y.o. woman with history of breast cancer (infiltrating ductal carcinoma, ER+, PR, and HER2 neg). Diffuse bone pain, no evidence of bone metastases on bone scintigraphy (**a** anterior view). *PET/CT findings*: highly heterogeneous uptake in the cranium, spine, pelvic grid and femurs, consistent with bone marrow involvement (**b** MIP). MRI confirms multiple small sized lesions (**c**)

Clinical indications in oncology (Figs. 70 and 71)

The indications are those of 99mTc-labelled diphosphonate bone scintigraphy. ^18^F-NaF PET/CT is more sensitive than bone scintigraphy, for most indications. The choice of PET or SPECT depends on the availability of the radiopharmaceuticals, PET/CT devices, and costs (Lofgren et al. n.d.).

## Conclusion

The constant growth of PET/CT including the increasing use of novel non-FDG PET/CT radiopharmaceuticals in cancer patients creates a need for training in the proper interpretation of complex imaging studies with compounds that have very different biodistribution, normal variants, and pitfalls. In addition, the use of several of these non-FDG PET radiopharmaceuticals, such as ^68^Ga-PSMA and ^68^Ga-DOTA peptides, constitutes an integral part of the evaluation of patients with cancer for theranostics. As this further increases the radiopharmaceuticals’ clinical relevance, there is also the need for accurate interpretation of non-FDG PET/CT studies.

## Data Availability

Yes (own data and materials)

## Abbreviations

5-HTTP: 5-Hydroxytryptophan
BCR: Biochemical recurrence
DOPA: Dihydroxyphenylalanine
DOTANOC: Tetraazacyclododecane-tetraacetic acid NaI3-octreotide
DOTATATE: Tetraazacyclododecane-tetraacetic acid (d)-Phe1-Thy3-octreotate
DOTATOC: Tetraazacyclododecane-tetraacetic acid (d)-Phe1-Thy3-octreotide
ER: Oestrogen receptors
FAZA: Fluorodeoxyarabinofuranosyl-nitroimidazole
FDG: Fluorodeoxyglucose
FES: Fluoroestradiol
FET: Fluoroethyltyrosine
FLAIR: Fluid attenuated inversion recovery
FLT: Fluorothymidine
FMISO: Fluoro-propanol-nitroimidazole
HCC: Hepatocellular carcinoma
HER2: Human epidermal growth factor receptor 2
MBq: Megabecquerel
MIBI: Methoxyisobutylisonitrile
MIP: Maximum intensity projection
MRI: Magnetic resonance imaging
MTC: Medullary thyroid cancer
NAF: Sodium fluoride
NET: Neuroendocrine tumours
NSCLC: Non-small cell lung cancer
PET/CT: Positron emission tomography/computed tomography
PR: Progesterone receptor
PSAdt: Prostate-specific antigen doubling time
PSMA: Prostate-specific antigen
PSMA: Prostate-specific membrane antigen
SPECT: Single-photon emission computed tomography
TTR: Time to relapse
y.o.: Years old

## References

[CR1] Abe K, Hayashi K, Sasaki M, Koga H, Kaneko K, Sawamoto H (2006). O-(2-[18F]fluoroethyl)-L-tyrosine (18F-FET) uptake in mouse thymoma cells, and its biodistribution in mice and human volunteers. Acta Radiol.

[CR2] Addeo P, Poncet G, Goichot B, Leclerc L, Brigand C, Mutter D (2018). The added diagnostic value of (18)F-Fluorodihydroxyphenylalanine PET/CT in the preoperative work-up of small bowel neuroendocrine tumors. J Gastrointest Surg.

[CR3] Afshar-Oromieh A, Hetzheim H, Kubler W, Kratochwil C, Giesel FL, Hope TA (2016). Radiation dosimetry of (68)Ga-PSMA-11 (HBED-CC) and preliminary evaluation of optimal imaging timing. Eur J Nucl Med Mol Imaging.

[CR4] Albert NL, Weller M, Suchorska B, Galldiks N, Soffietti R, Kim MM (2016). Response assessment in neuro-oncology working group and European association for neuro-oncology recommendations for the clinical use of PET imaging in gliomas. Neuro-Oncology.

[CR5] Amodru V, Guerin C, Delcourt S, Romanet P, Loundou A, Viana B (2018). Quantitative (18)F-DOPA PET/CT in pheochromocytoma: the relationship between tumor secretion and its biochemical phenotype. Eur J Nucl Med Mol Imaging.

[CR6] Beheshti M, Mottaghy FM, Paycha F, Behrendt FFF, Van den Wyngaert T, Fogelman I (2015). (18)F-NaF PET/CT: EANM procedure guidelines for bone imaging. Eur J Nucl Med Mol Imaging.

[CR7] Bergeret S, Charbit J, Ansquer C, Bera G, Chanson P, Lussey-Lepoutre C (2019). Novel PET tracers: added value for endocrine disorders. Endocrine..

[CR8] Bollineni VR, Kerner GS, Pruim J, Steenbakkers RJ, Wiegman EM, Koole MJ (2013). PET imaging of tumor hypoxia using 18F-fluoroazomycin arabinoside in stage III-IV non-small cell lung cancer patients. J Nucl Med.

[CR9] Bollineni VR, Koole MJ, Pruim J, Brouwer CL, Wiegman EM, Groen HJ (2014). Dynamics of tumor hypoxia assessed by 18F-FAZA PET/CT in head and neck and lung cancer patients during chemoradiation: possible implications for radiotherapy treatment planning strategies. Radiother Oncol.

[CR10] Bruine de Bruin L, Bollineni VR, Wachters JE, Schuuring E, van Hemel BM, van der Wal JE (2015). Assessment of hypoxic subvolumes in laryngeal cancer with (18)F-fluoroazomycinarabinoside ((18)F-FAZA)-PET/CT scanning and immunohistochemistry. Radiother Oncol.

[CR11] Calais J, Fendler WP, Eiber M, Gartmann J, Chu FI, Nickols NG (2018). Impact of (68)Ga-PSMA-11 PET/CT on the management of prostate cancer patients with biochemical recurrence. J Nucl Med.

[CR12] Chondrogiannis S, Marzola MC, Al-Nahhas A, Venkatanarayana TD, Mazza A, Opocher G (2013). Normal biodistribution pattern and physiologic variants of 18F-DOPA PET imaging. Nucl Med Commun.

[CR13] Davis J, Yano Y, Cahoon J, Budinger TF (1982). Preparation of 11C-methyl iodide and L-[S-methyl-11C]methionine by an automated continuous flow process. Int J Appl Radiat Isot.

[CR14] DeGrado TR, Coleman RE, Wang S, Baldwin SW, Orr MD, Robertson CN (2001). Synthesis and evaluation of 18F-labeled choline as an oncologic tracer for positron emission tomography: initial findings in prostate cancer. Cancer Res.

[CR15] DeGrado TR, Reiman RE, Price DT, Wang S, Coleman RE (2002). Pharmacokinetics and radiation dosimetry of 18F-fluorocholine. J Nucl Med.

[CR16] Deloar HM, Fujiwara T, Nakamura T, Itoh M, Imai D, Miyake M (1998). Estimation of internal absorbed dose of L-[methyl-11C]methionine using whole-body positron emission tomography. Eur J Nucl Med.

[CR17] Demirci E, Sahin OE, Ocak M, Akovali B, Nematyazar J, Kabasakal L (2016). Normal distribution pattern and physiological variants of 68Ga-PSMA-11 PET/CT imaging. Nucl Med Commun.

[CR18] Evangelista L, Guttilla A, Zattoni F, Muzzio PC, Zattoni F (2013). Utility of choline positron emission tomography/computed tomography for lymph node involvement identification in intermediate- to high-risk prostate cancer: a systematic literature review and meta-analysis. Eur Urol.

[CR19] Fendler WP, Eiber M, Beheshti M, Bomanji J, Ceci F, Cho S (2017). (68)Ga-PSMA PET/CT: Joint EANM and SNMMI procedure guideline for prostate cancer imaging: version 1.0. Eur J Nucl Med Mol Imaging.

[CR20] Gagel B, Reinartz P, Demirel C, Kaiser HJ, Zimny M, Piroth M (2006). [18F] fluoromisonidazole and [18F] fluorodeoxyglucose positron emission tomography in response evaluation after chemo-/radiotherapy of non-small-cell lung cancer: a feasibility study. BMC Cancer.

[CR21] Galldiks N, Stoffels G, Filss C, Rapp M, Blau T, Tscherpel C (2015). The use of dynamic O-(2-18F-fluoroethyl)-l-tyrosine PET in the diagnosis of patients with progressive and recurrent glioma. Neuro-Oncology.

[CR22] Grierson JR, Shields AF (2000). Radiosynthesis of 3'-deoxy-3'-[(18)F]fluorothymidine: [(18)F]FLT for imaging of cellular proliferation in vivo. Nucl Med Biol.

[CR23] Hain SF, Maisey MN (2003). Positron emission tomography for urological tumours. BJU Int.

[CR24] Harris SM, Davis JC, Snyder SE, Butch ER, Vavere AL, Kocak M (2013). Evaluation of the biodistribution of 11C-methionine in children and young adults. J Nucl Med.

[CR25] Heidenreich A, Bastian PJ, Bellmunt J, Bolla M, Joniau S, van der Kwast T (2014). EAU guidelines on prostate cancer. Part II: Treatment of advanced, relapsing, and castration-resistant prostate cancer. Eur Urol.

[CR26] Hirata K, Terasaka S, Shiga T, Hattori N, Magota K, Kobayashi H (2012). (1)(8)F-Fluoromisonidazole positron emission tomography may differentiate glioblastoma multiforme from less malignant gliomas. Eur J Nucl Med Mol Imaging.

[CR27] Ho CL, Yu SC, Yeung DW (2003). 11C-acetate PET imaging in hepatocellular carcinoma and other liver masses. J Nucl Med.

[CR28] Institute NC. Investigator’s Brochure for [18F] fluoromisonidazole, 1H-1-(3-[18F]-fluoro-2-hydroxy-propyl)-2-nitro-imidazole, [18F]FMISO. An investigational positron emission tomography (PET) radiopharmaceutical for injection and intended for use as an in vivo diagnostic for imaging hypoxia in tumors. NIH;5^th^ ed, 2013.

[CR29] Karanikas G, Beheshti M (2014). (1)(1)C-acetate PET/CT imaging: physiologic uptake, variants, and pitfalls. PET Clin.

[CR30] Kenny L, Coombes RC, Vigushin DM, Al-Nahhas A, Shousha S, Aboagye EO (2007). Imaging early changes in proliferation at 1 week post chemotherapy: a pilot study in breast cancer patients with 3'-deoxy-3'-[18F]fluorothymidine positron emission tomography. Eur J Nucl Med Mol Imaging.

[CR31] Kratochwil C, Bruchertseifer F, Rathke H, Bronzel M, Apostolidis C, Weichert W (2017). Targeted alpha-therapy of metastatic castration-resistant prostate cancer with (225)Ac-PSMA-617: dosimetry estimate and empiric dose finding. J Nucl Med.

[CR32] Kroiss A, Putzer D, Decristoforo C, Uprimny C, Warwitz B, Nilica B (2013). 68Ga-DOTA-TOC uptake in neuroendocrine tumour and healthy tissue: differentiation of physiological uptake and pathological processes in PET/CT. Eur J Nucl Med Mol Imaging.

[CR33] Kryza D, Tadino V, Filannino MA, Villeret G, Lemoucheux L (2008). Fully automated [18F]fluorocholine synthesis in the TracerLab MX FDG Coincidence synthesizer. Nucl Med Biol.

[CR34] Kunz M, Thon N, Eigenbrod S, Hartmann C, Egensperger R, Herms J (2011). Hot spots in dynamic (18)FET-PET delineate malignant tumor parts within suspected WHO grade II gliomas. Neuro-Oncology.

[CR35] Liao GJ, Clark AS, Schubert EK, Mankoff DA (2016). 18F-Fluoroestradiol PET: current status and potential future clinical applications. J Nucl Med.

[CR36] Lin Z, Mechalakos J, Nehmeh S, Schoder H, Lee N, Humm J (2008). The influence of changes in tumor hypoxia on dose-painting treatment plans based on 18F-FMISO positron emission tomography. Int J Radiat Oncol Biol Phys.

[CR37] Linden HM, Kurland BF, Peterson LM, Schubert EK, Gralow JR, Specht JM (2011). Fluoroestradiol positron emission tomography reveals differences in pharmacodynamics of aromatase inhibitors, tamoxifen, and fulvestrant in patients with metastatic breast cancer. Clin Cancer Res.

[CR38] Liu RS, Chang CP, Chu LS, Chu YK, Hsieh HJ, Chang CW (2006). PET imaging of brain astrocytoma with 1-11C-acetate. Eur J Nucl Med Mol Imaging.

[CR39] Löfgren Johan, Mortensen Jann, Rasmussen Sine H., Madsen Claus, Loft Annika, Hansen Adam E., Oturai Peter, Jensen Karl Erik, Mørk Mette Louise, Reichkendler Michala, Højgaard Liselotte, Fischer Barbara M. (2017). A Prospective Study Comparing99mTc-Hydroxyethylene-Diphosphonate Planar Bone Scintigraphy and Whole-Body SPECT/CT with18F-Fluoride PET/CT and18F-Fluoride PET/MRI for Diagnosing Bone Metastases. Journal of Nuclear Medicine.

[CR40] Lopci E, Grassi I, Chiti A, Nanni C, Cicoria G, Toschi L (2014). PET radiopharmaceuticals for imaging of tumor hypoxia: a review of the evidence. Am J Nucl Med Mol Imaging.

[CR41] Maurer T, Gschwend JE, Rauscher I, Souvatzoglou M, Haller B, Weirich G (2016). Diagnostic efficacy of (68)Gallium-PSMA positron emission tomography compared to conventional imaging for lymph node sStaging of 130 consecutive patients with intermediate to high risk prostate cancer. J Urol.

[CR42] Mitterhauser M, Wadsak W, Krcal A, Schmaljohann J, Eidherr H, Schmid A (2005). New aspects on the preparation of [11C]Methionine--a simple and fast online approach without preparative HPLC. Appl Radiat Isot.

[CR43] Mottet N, Bellmunt J, Bolla M, Joniau S, Mason M, Matveev V (2011). EAU guidelines on prostate cancer. Part II: Treatment of advanced, relapsing, and castration-resistant prostate cancer. Eur Urol.

[CR44] Neels OC, Jager PL, Koopmans KP, Eriks E, de Vries EG, Kema IP (2006). Development of a reliable remote-controlled synthesis of β-[11C]-5-hydroxy-L-tryptophan on a Zymark robotic system. J Lab Compounds Radiopharmaceuticals.

[CR45] Nehmeh SA, Lee NY, Schroder H, Squire O, Zanzonico PB, Erdi YE (2008). Reproducibility of intratumor distribution of (18)F-fluoromisonidazole in head and neck cancer. Int J Radiat Oncol Biol Phys.

[CR46] Oh SJ, Mosdzianowski C, Chi DY, Kim JY, Kang SH, Ryu JS (2004). Fully automated synthesis system of 3'-deoxy-3'-[18F]fluorothymidine. Nucl Med Biol.

[CR47] Orlefors H, Sundin A, Garske U, Juhlin C, Oberg K, Skogseid B (2005). Whole-body 11C-5-hydroxytryptophan positron emission tomography as a universal imaging technique for neuroendocrine tumors: comparison with somatostatin receptor scintigraphy and computed tomography. J Clin Endocrinol Metab.

[CR48] Park JW, Kim JH, Kim SK, Kang KW, Park KW, Choi JI (2008). A prospective evaluation of 18F-FDG and 11C-acetate PET/CT for detection of primary and metastatic hepatocellular carcinoma. J Nucl Med.

[CR49] Peterson LM, Kurland BF, Link JM, Schubert EK, Stekhova S, Linden HM (2011). Factors influencing the uptake of 18F-fluoroestradiol in patients with estrogen receptor positive breast cancer. Nucl Med Biol.

[CR50] Piccardo A, Lopci E, Conte M, Garaventa A, Foppiani L, Altrinetti V (2012). Comparison of 18F-dopa PET/CT and 123I-MIBG scintigraphy in stage 3 and 4 neuroblastoma: a pilot study. Eur J Nucl Med Mol Imaging.

[CR51] Poulsen SH, Urup T, Grunnet K, Christensen IJ, Larsen VA, Jensen ML (2017). The prognostic value of FET PET at radiotherapy planning in newly diagnosed glioblastoma. Eur J Nucl Med Mol Imaging.

[CR52] Rahbar K, Ahmadzadehfar H, Kratochwil C, Haberkorn U, Schafers M, Essler M (2017). German multicenter study investigating 177Lu-PSMA-617 radioligand therapy in advanced prostate cancer patients. J Nucl Med.

[CR53] Reischl G, Dorow DS, Cullinane C, Katsifis A, Roselt P, Binns D (2007). Imaging of tumor hypoxia with [124I]IAZA in comparison with [18F]FMISO and [18F]FAZA--first small animal PET results. J Pharm Pharm Sci.

[CR54] Sandblom G, Sorensen J, Lundin N, Haggman M, Malmstrom PU (2006). Positron emission tomography with C11-acetate for tumor detection and localization in patients with prostate-specific antigen relapse after radical prostatectomy. Urology..

[CR55] Schneider B, Zhu H, Ma X, Cheng Z, Iagaru A, Kopka K (2016). Preparation and chemical analysis of clinical-grade 68Ga-PSMA-HBED-CC, an emerging tracer for imaging of prostate cancers. J Nucl Med.

[CR56] Seltzer MA, Jahan SA, Sparks R, Stout DB, Satyamurthy N, Dahlbom M (2004). Radiation dose estimates in humans for (11)C-acetate whole-body PET. J Nucl Med.

[CR57] Shankar LK (2012). The clinical evaluation of novel imaging methods for cancer management. Nat Rev Clin Oncol.

[CR58] Singh S, Poon R, Wong R, Metser U (2018). 68Ga PET imaging in patients with neuroendocrine tumors: a systematic review and meta-analysis. Clin Nucl Med.

[CR59] Skoura E, Michopoulou S, Mohmaduvesh M, Panagiotidis E, Al Harbi M, Toumpanakis C (2016). The impact of 68Ga-DOTATATE PET/CT imaging on management of patients with neuroendocrine tumors: experience from a national referral center in the United Kingdom. J Nucl Med.

[CR60] Soussan M, Nataf V, Kerrou K, Grahek D, Pascal O, Talbot JN (2012). Added value of early 18F-FDOPA PET/CT acquisition time in medullary thyroid cancer. Nucl Med Commun.

[CR61] Sundin A (2018). Novel functional imaging of neuroendocrine tumors. Endocrinol Metab Clin N Am.

[CR62] Turcotte E, Wiens LW, Grierson JR, Peterson LM, Wener MH, Vesselle H (2007). Toxicology evaluation of radiotracer doses of 3'-deoxy-3'-[18F]fluorothymidine (18F-FLT) for human PET imaging: laboratory analysis of serial blood samples and comparison to previously investigated therapeutic FLT doses. BMC Nucl Med.

[CR63] Unterrainer M, Schweisthal F, Suchorska B, Wenter V, Schmid-Tannwald C, Fendler WP (2016). Serial 18F-FET PET imaging of primarily 18F-FET-negative glioma: does it make sense?. J Nucl Med.

[CR64] van Kruchten M, de Vries EGE, Brown M, de Vries EFJ, Glaudemans A, Dierckx R (2013). PET imaging of oestrogen receptors in patients with breast cancer. Lancet Oncol.

[CR65] van Kruchten M, Glaudemans AW, de Vries EF, Beets-Tan RG, Schroder CP, Dierckx RA (2012). PET imaging of estrogen receptors as a diagnostic tool for breast cancer patients presenting with a clinical dilemma. J Nucl Med.

[CR66] van Kruchten M, Hospers GA, Glaudemans AW, Hollema H, Arts HJ, Reyners AK (2013). Positron emission tomography imaging of oestrogen receptor-expression in endometrial stromal sarcoma supports oestrogen receptor-targeted therapy: case report and review of the literature. Eur J Cancer.

[CR67] Venema CM, Apollonio G, Hospers GA, Schroder CP, Dierckx RA, de Vries EF (2016). Recommendations and technical aspects of 16alpha-[18F]Fluoro-17beta-estradiol PET to image the estrogen receptor in vivo: the Groningen experience. Clin Nucl Med.

[CR68] Vesselle H, Grierson J, Peterson LM, Muzi M, Mankoff DA, Krohn KA (2003). 18F-Fluorothymidine radiation dosimetry in human PET imaging studies. J Nucl Med.

[CR69] Visser AK, Ramakrishnan NK, Willemsen AT, Di Gialleonardo V, de Vries EF, Kema IP (2014). [(11)C]5-HTP and microPET are not suitable for pharmacodynamic studies in the rodent brain. J Cereb Blood Flow Metab.

[CR70] Wack LJ, Monnich D, van Elmpt W, Zegers CM, Troost EG, Zips D (2015). Comparison of [18F]-FMISO, [18F]-FAZA and [18F]-HX4 for PET imaging of hypoxia--a simulation study. Acta Oncol.

[CR71] Waseem N, Aparici CM, Kunz PL (2019). Evaluating the role of theranostics in grade 3 neuroendocrine neoplasms. J Nucl Med.

[CR72] Zamboglou C, Wieser G, Hennies S, Rempel I, Kirste S, Soschynski M (2016). MRI versus (6)(8)Ga-PSMA PET/CT for gross tumour volume delineation in radiation treatment planning of primary prostate cancer. Eur J Nucl Med Mol Imaging.

[CR73] Zips D, Zophel K, Abolmaali N, Perrin R, Abramyuk A, Haase R (2012). Exploratory prospective trial of hypoxia-specific PET imaging during radiochemotherapy in patients with locally advanced head-and-neck cancer. Radiother Oncol.

